# Women with metabolic syndrome show similar health benefits from high-intensity interval training than men

**DOI:** 10.1371/journal.pone.0225893

**Published:** 2019-12-10

**Authors:** Valle Guio de Prada, Juan Fernando Ortega, Felix Morales-Palomo, Miguel Ramirez-Jimenez, Alfonso Moreno-Cabañas, Ricardo Mora-Rodriguez

**Affiliations:** 1 Sports Medicine Center, Diputacion de Toledo, Toledo, Spain; 2 Exercise Physiology Laboratory, University of Castilla-La Mancha, Toledo, Spain; University of Alabama at Birmingham, UNITED STATES

## Abstract

High-intensity interval training (HIIT), is effective to improve cardiorespiratory fitness (CRF) and metabolic syndrome (MetS) components in adults. However, it is unclear if CRF and MetS components respond similarly in men and women after HIIT. For 16 weeks, 63 women (53±7 years) and 56 men (55±8 years) with MetS underwent a three day/week HIIT program. Bodyweight and composition, VO_2MAX_, surrogate parameters of CRF (Ventilatory threshold (VT), oxygen uptake efficiency slope (OUES) and VE/VCO_2_ slope), maximal rate of fat oxidation (MFO), and MetS components were assessed before and after training. All reported variables were analyzed by split-plot ANOVA looking for time by sex interactions. Before training men had higher absolute values of VO_2MAX_ (58.6%), and MFO (24.6%), while lower body fat mass (10.5%) than women (all P<0.05). After normalization by fat-free mass (FFM), VO_2MAX_ remained 16.6% higher in men (P<0.05), whereas differences in MFO disappeared (P = 0.292). After intervention VO_2MAX_ (P<0.001), VO_2_ at VT (P<0.001), OUES (P<0.001), and VE/VCO2 slope (P<0.001) increased without differences by sex (P>0.05). After training MetS Z-score (P<0.001) improved without differences between men and women (P>0.05). From the MetS components, only blood pressure (P<0.001) and waist circumference (P<0.001) improved across time, without differences by sex. In both, women and men, changes in OUES (r = 0.685 and r = 0.445, respectively), and VO_2_ at VT (r = 0.378, and r = 0.445, respectively), correlated with VO_2MAX_. While only bodyweight changes correlated with MetS Z-score changes (r = 0.372, and = 0.300, respectively). Despite baseline differences, 16-weeks of HIIT similarly improved MetS, cardiorespiratory and metabolic fitness in women and men with MetS. This suggests that there are no restrictions due to sex on the benefits derived from an intense exercise program in the health of MetS participants.

Trial Registration: clinicaltrials.gov NCT03019796

## Introduction

According to the National Health and Nutritional Examination Survey (NHANES), in the United States of America, the prevalence of metabolic syndrome (MetS) is increasing, mainly in women [1]. Endurance exercise training and in particular high-intensity interval training (HIIT), is effective to improve MetS components in middle-aged adults [2]. However, it is unclear if MetS components respond similarly in men and women after endurance training. On the one hand, Morita et al. [3], studied the effects of moderate-intensity cycling training in adults with cardiovascular risk factors. The authors found that after adjustment for confounding factors (age, baseline value and changes in body weight), reductions in blood pressure after exercise training were greater in women than in men. On the other hand, 20 weeks of supervised continuous exercise training, in the HERITAGE Family Study, showed that exercise training was similarly effective against MetS in women than in men [4]. Thus, although aerobic training is a powerful tool to improve MetS, there are contradictory findings of the gender-related differences in the response of MetS to continuous training. Moreover, to our knowledge, there is scarce evidence about the gender-related differences in the responses after HIIT in patients diagnosed with MetS.

Low levels of cardiorespiratory fitness (CRF) have been associated with the clustering of metabolic abnormalities that compose the MetS [5][6]. Regarding CRF differences between women and men, it is well established that maximal oxygen consumption (i.e., VO_2MAX_), is lower in women than in men [7–12], due to women’s smaller muscle mass, hemoglobin content and heart size [13]. According to the American College of Sports Medicine [14], gender has little influence on the variability of CRF response to exercise training. However, Howden et al, [15] found that after 1 year of continuous moderate intensity training, women showed a significant less improvement of VO_2MAX_. Concerning HIIT, previous studies conducted both, in healthy and active young adults [16] and sedentary adult women and men [17], did not find a significant gender-related difference in the improvements of CRF, while data on middle-aged MetS subjects is scarce. Thus, it seems that in spite of the current information provided by exercise guidelines, there is conflicting evidence about the responses of women and men after exercise training. Particularly for HIIT, less is known, about the comparative progression of VO_2MAX_ in women and men with cardiometabolic disorders such as MetS.

VO_2MAX_ obtained from a graded exercise test (GTX) is the gold standard method to assess CRF. However, VO_2MAX_ determination could be difficult in deconditioned individuals, who have poor tolerance to vigorous intensities [18]. For these reasons, ventilatory threshold (VT) [19], and more recently two additional parameters obtained during the GXT, have emerged as reliable indicators of CRF [18]. *i*. Oxygen uptake efficiency slope (OUES), an index of cardiorespiratory functional reserve [20], that assess oxygen delivery and extraction efficiency. *ii*. The slope of the regression between minute ventilation and CO_2_ production (i.e., VE/VCO_2_ slope), that represents the matching of ventilation and perfusion within the pulmonary system, at least in patients with cardiovascular disease [21, 22]. To our knowledge, neither the overall nor the gender-discriminated response of these surrogate indices of CRF have been reported in patients with MetS after HIIT.

Together with the assessment of MetS components and CRF, the study of fat metabolism is of great relevance in patients diagnosed with MetS. The maximal rate of fat oxidation during exercise (MFO) has been associated with insulin sensitivity a linking factor in the development of MetS [23]. This link might be explained by mitochondrial activity, given the main processes involved in energy transformation from fat occur in the mitochondria [24–26]. Broadly, mitochondrial dysfunction leads to the accumulation of intracellular lipids, resulting in impaired insulin signaling [27]. Current evidence has shown that in people with obesity, MFO is generally greater in men compared to women. However, after normalizing for fat-free mass, differences tend to disappear [28], or even invert in favor of women [29]. Regarding MFO responses after HIIT, Astorino et al found in active participants that after 6 sessions of sprint interval training (i.e., repeated Wingate tests efforts), MFO change was similar between women and men [16]. More recently, the same group found that after 20 sessions of HIIT, a group of active and young men and women did not show a significant improvement in MFO [30]. Although responses after HITT has been addressed in active and healthy population, little is known about the differential response of MFO after training in women and men with MetS.

Most of the literature coincides on that gender does not differentially affect the gains in VO_2MAX_ elicited by continuous endurance training in young healthy people. However scarce evidence is available about the effects of HIIT between middle-aged women and men with increased cardiometabolic risk. Therefore, we aimed to study the effect of gender on the progression of cardiorespiratory fitness, maximal fat oxidation during exercise, and MetS components, in a group of MetS patients undergoing 16 weeks of high-intensity interval training. We hypothesized that women’s lower capacity to generate muscle power during exercise would reflect in lesser cardiometabolic benefit from HIIT. A comparison of the responses separated by gender after HIIT, could improve the individualization of training programs using this modality.

## Methods

### Study population

**Fig 1:**
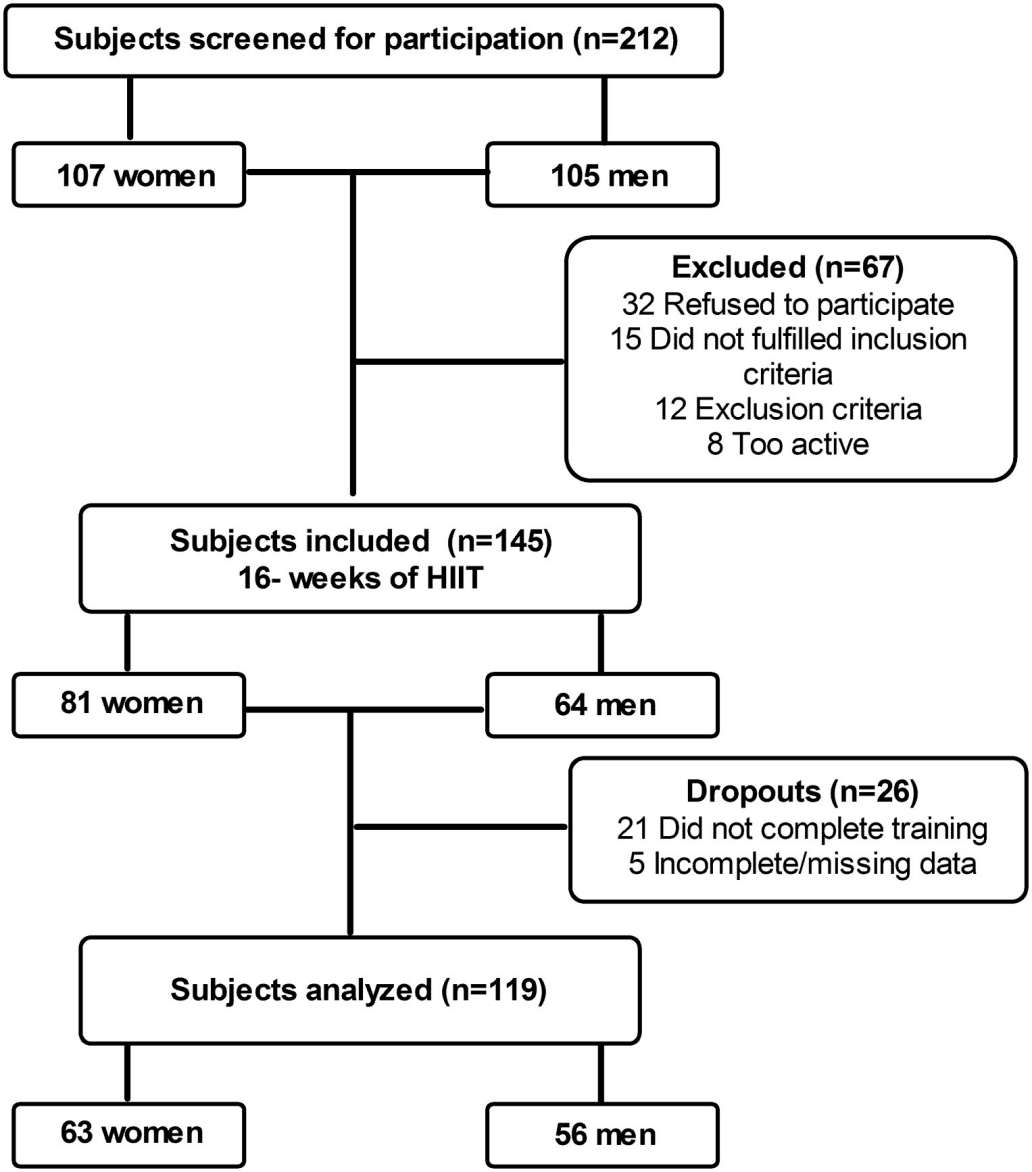
Flow chart of the Consolidated Standards of Reporting Trials (CONSORT) procedures followed in our study.

Fig 1 displays the flowchart of volunteers screened, tested and included in the study analysis. We analyzed 119 individuals fulfilling three or more MetS criteria using Europid population cut points for waist circumference [31], before and after a 4-month exercise training program. Volunteers were all Caucasians European descent between 31 and 72 years old (women were 52.5 ± 7.0 and men were 54.8 ± 7.6 years old, P = 0.103). Participants’ recruitment and follow-up were conducted between January 2012 and March 2017. Volunteers were recruited from the population of Toledo`s inhabitants via flyers, posters, and radio advertising to participate in a study designed to compare the effects of aerobic training versus a combination of aerobic and strength training. Volunteers included in this study were part of the branch devoted to aerobic training alone in the study: “Effects of 16 weeks of training in metabolic syndrome components, temporal evolution of improvements”. There were 63 women (22 pre-menopausal), and 56 men in the group. The required sample size was obtained using standard calculations, using VO_2_max as the main variable. To obtain a pre-post-training difference of 12% in VO_2_max (α value of 0.05 (two tails)), a sample size of n = 114 was required. Calculations were based on the standard deviation and improvement after training observed in a group of 48 subjects tested in the past. All procedures were performed in accordance with a protocol approved by the Albacete University Hospital’s ethics committee. (Albacete. Spain), and conformed to the latest revision of Declaration of Helsinki. Before experimental testing, participants underwent medical screening which included physical history and examination, and a resting 12-lead standard ECG. Exclusion criteria included non-treated cardiovascular, respiratory or metabolic disease, and any disease associated with exercise intolerance. Subjects were instructed to continue with their nutritional habits and current medication prescriptions throughout the study.

### Exercise training

Volunteers underwent, supervised stationary cycling HIIT with a frequency of 3 times per week, for 43-min for 16 weeks. Exercise sessions were conducted in a room inside the University of Castilla-La Mancha campus. Training consisted of a warm-up pedaling for 10-min at 70% of peak heart rate (HR_PEAK_), followed by 4x4-min intervals at 90% HR_PEAK_ interspersed with 3-min active recovery at 70% HR_PEAK_, and a cool-down period of 5-min. During all training sessions, individual heart rate was monitored and projected in a screen in real-time using ANT+ telemetric chest bands and associated software (Seego, Realtrack systems, Spain). Participants were required to attend at least 85% of all the exercise sessions, to be included in the post-training evaluation. All exercise training sessions were supervised by members of the research group. Aiming to improve intervention fidelity as described by Taylor et al [32], supervision included verbal encouragement during each interval to motive attainment of target HR at the second minute of each 4-min interval.

### Experimental design

Before and at least 3 days after the last training session of the 16-week HIIT program, participants reported to the exercise physiology laboratory of the University of Castilla-La Mancha in the morning after an overnight fast. Firstly, body weight (Hawk, Mettler Toledo, China), waist circumference (Rosscraft Anthrotape, USA), body composition and resting supine blood pressure were collected. Following, 10 cc blood samples were obtained for blood chemistry assessment. Following, a submaximal test was performed to assess the maximal rate of fat oxidation (MFO; [33]) which was used as an index of metabolic fitness. Then, cardiorespiratory fitness was assessed using a graded exercise testing (GXT) until volitional fatigue followed by a confirmation test after 10 minutes of rest. Pre-menopausal women (40%; n = 22) were tested in the same phase of their menstrual cycle before and after training (i.e., 18 in the follicular phase and 4 in the luteal phase).

### Cardiorespiratory fitness

Before and after training participants underwent a GXT with one-minute stages until volitional exhaustion on an electrically braked cycle-ergometer (Ergoselect 200, Ergoline, Germany). GXT started with a 3-minute warm-up period at 15 W for women or 20 W for men, followed by a workload increase of 15–20 W per minute for women and men, respectively, until exhaustion During testing, expiratory volume, O_2_, and CO_2_ concentrations were continuously monitored using an automated breath-by-breath system (Quark B^2^, Cosmed, Italy). Integrated standard 12-lead ECG (Quark T12, Cosmed, Italy), and blood pressure were monitored during each stage of the GXT to ensure that all subjects had a normal cardiovascular response to exercise. The maximal heart rate achieved (HR_PEAK_) during the test was recorded and used to prescribe training intensity during the HIIT program. Predicted values of VO_2MAX_ were calculated according to Hansen et al [20]. Using the data provided by the breath by breath gas analyzer, ventilatory threshold (VT) was identified using the V-slope method as described by Sue et al. [34] according to OUES was calculated from the linear relation of VO_2_ versus the logarithm of VE during exercise: VO_2_ (mL·min^-1^) = (*m* · log10 VE (L·min^-1^)) + *b*. The slope *m* in this formula represents the rate of increase in VO_2_ in response to VE and *b* is the intercept [18]. VE/VCO_2_ slope was defined as the slope of the relationship between ventilation and carbon dioxide production throughout the entire exercise test, using the equation VE (L·min^-1^) = (*m* · VCO_2_ (L·min^-1^)) + *b*, where *m* = VE/VCO_2_ slope [35]. Fifteen minutes after the GXT, a verification test at 110% of the peak power output during GTX was conducted to confirm VO_2MAX_ attainment [36]. Only volunteers who fulfilled a true VO_2MAX_ criteria according to Poole and Jones were included in the study [36].

### Maximal fat oxidation assessment

The submaximal test started at 10W in women and at 30W in men. Every 4 minutes workload increased by 10W in women and 15W in men until respiratory quotient reached 1.0. Averages of breath by breath VO_2_ and VCO_2_ from the last minute of each stage were used to calculate fat oxidation rate according to Jeukendrup and Wallis [37, 38].

### Body composition and blood chemistry

Waist circumference (WCirc) was measured in a horizontal plane, midway between the inferior margin of the ribs and the superior border of the iliac crest (Rosscraft Anthrotape, USA). Body fat mass (FM) and fat-free mass (FFM), were determined by dual-energy X-ray absorptiometry (DXA Hologic Series Discovery Wi QDR, Bedford, USA). Fasting plasma glucose (FG) was analyzed using the glucose oxidase-peroxidase method with intra/inter assay coefficient of variation (iCV) of 0.9–1.2%. High-density lipoprotein (HDL) using accelerator selective detergent method (iCV; 1.7–2.9%). Blood triglycerides (TG) with glycerol-3-phosphate oxidize method (iCV; 0.8–1.7%). All the above analyses were run in automated chemistry analyzer (Mindray BS 400, Mindray Medical Instrumentation, USA).

### Metabolic syndrome Z-scores

The MetS Z-score is a continuous score that comprises the five MetS variables. Z-scores were calculated to assess the evolution to the norm of MetS risk factors [39]. The sum of the Z-scores for each MetS components was divided by five to compile the MetS risk score with units of SD [40].

### Statistical analyses

Results are presented as means ±SD. Kolmogorov-Smirnov test revealed that data was normally distributed. Baseline differences between women and men were studied using the t-test for unrelated measurements. Split-plot ANOVA was run to analyze differences across time (repeated measures) and between experimental groups across time (time by gender) in all reported variables. Vertical multiple comparisons among pairwise group means were performed with a Bonferroni correction for type I error when the Time by Group interaction was significant. Effect size (ES) was assessed by η^2^ obtained from the ANOVA with small (η^2^ = 0.01), medium (η^2^ = 0.06), and large (η^2^ = 0.14) effects defined according to Cohen [41]. Correlation between submaximal parameters of CRF and VO_2MAX_ were obtained using Pearson’s correlation coefficient. SPSS, v22 (IBM Corporation) was used for statistical analysis and statistical significance set at P<0.05.

## Results

### VO2MAX

No adverse events were reported among the volunteers who participated in the study, neither during training nor during exercise testing. At the end of the training program, average attendance of participants included in the study was 94.6 ± 0.8%. Only participants who attend more than 85% of the training sessions were included on the final analysis. VO_2MAX_ at baseline, and after HIIT are displayed in Table 1. Estimation of the predicted VO_2PEAK_ adjusted by gender and bodyweight, showed that before intervention both, women and men were below the predicted values. However, after training, both women and men exceeded the predicted VO_2MAX_ values. Before intervention, VO_2MAX_, either expressed as absolute values (L·min^-1^) or normalized by body weight or fat-free mass (i.e., ml·kg^-1^·min^-1^) was higher in men (all P<0.05). VO_2MAX_, expressed in absolute values or normalized by body weight or fat-free mass increased after training (time ES = 0.553, ES = 0.585, and ES = 0.542, respectively), similarly between women and men (time by gender ES = 0.022, ES = 0.010, and ES = 0.006, respectively). After training 60.3% of women and 44,6% of men showed a VO_2MAX_ improvement superior to 10%, whereas 16.1% o men and 19.0% of women showed an improvement inferior to 1% or no improvement.

**Table 1:** Progression of the CRF and maximal fat oxidation after training.

	Women		Men		P	
	Pre	Post	Pre	Post	Time	Time by Gender
VO_2MAX_ (L·min^-1^)	1.48 ± 0.31	1.72 ± 0.34	2.34 ± 0.47*	2.66 ± 0.55	<0.001	0.110
VO_2MAX_ (mL·kg^-1^·min^-1^)	18.4 ± 3.7	21.5 ± 4.3	25.1 ± 4.8*	28.7 ± 5.1	<0.001	0.280
VO_2MAX_ (mL·kg^-1^ FFM·min^-1^)	31.68 ± 5.56	37.25 ± 6.37	36.95 ± 6.12*	41.77 ± 6.94	<0.001	0.394
Pct. of pred VO_2MAX_ (%)	95 ± 18	111± 19	98 ± 18	111± 19	<0.001	0.388
VO_2_ at VT (L·min_-1_)	0.84 ± 0.03	0.97 ± 0.03	1.29 ± 0.03	1.48 ± 0.04	<0.001	0.184
OUES	1590± 368	1712± 407	2464± 574*	2594± 519	<0.001	0.915
VE/VCO_2_ slope (ml·min^-1^)	32.6± 5.2	35.3± 5,2	32.8± 6.6	34.7± 6.2	<0.001	0.484
MFO (g/min)	0.19 ± 0.08	0.23 ± 0.09	0.24 ± 0.08*	0.31 ± 0.12	<0.001	0.095
MFO (mg·kg^-1^ FFM·min^-1^)	4.10 ± 1.54	5.01± 1.77	3.82 ± 1.35	4.82 ± 1.82	<0.001	0.713

*Different from women at baseline

### Surrogate parameters of CRF

Surrogate parameters of CRF at baseline, and after HIIT are displayed in Table 1. At baseline, VO_2_ at VT was higher in men (P<0.001). However, after training both groups improved without differences by gender (P = 0.184, ES = 0.094). At baseline, power output (PO) at VT was higher in men than in women (83 ± 23W and 51 ± 18 W, respectively). After training, PO at VT improved (P<0.001) without differences between in women and men (P = 0.121). PO at VT improved meaningfully(>10%) in 64,3% of men and 60.3% of women. Before training OUES values were higher in men than in women and this difference was maintained after intervention since OUES increased similarly in both groups (time ES = 0.087, and time by gender ES<0.001). At baseline, VE/VCO_2_ slope was similar in both groups (P = 0.861). After training, VE/VCO_2_ slope increased similarly in women and men (time ES = 0.152, time by gender ES = 0.004).

### Maximal fat oxidation during exercise

MFO at baseline, and after HIIT are displayed in Table 1. At baseline, absolute values of MFO were higher in men than in women, (P<0.05). However, when normalized by fat-free mass (FFM) differences disappeared, (P = 0.292). After training, absolute and normalized MFO values, increased significantly (time ES = 0.274 and ES = 0.263, respectively) in a similar fashion in both groups (time by gender ES = 0.024; and ES = 0.001, respectively).

### MetS components and Z-score

MetS components and MetS Z-score are displayed in Table 2. Before exercise training, HDL was lower in men (P<0.001) whereas WCirc was higher in men (P<0.001). FG, TG, systolic blood pressure (SBP) and diastolic blood pressure (DBP) were not different between groups and thus, the compiled MetS Z-score was similar between women and men (P = 0.447). After training, SBP (ES = 0.382), DBP (ES = 0.353), WCirc (ES = 0.392), and Mets Z-score (ES = 0.387) improved significantly without differences between women and men (all time by gender P>0.05). TG, HDL, and FG did not change significantly across time in any group (P>0.05; Table 2).

**Table 2:** Metabolic syndrome components after 16 weeks of high-intensity interval training. Data are means ± SD.

	Women		Men		P	
	Pre	Post	Pre	Post	Time	Time by Gender
TG (mmol·L^-1^)	1.39± 0.73	1.37± 0.79	1.62± 0.75	1.56± 0.76	0.439	0.708
HDL (mmol·L^-1^)	1.31± 0.37	1.30± 0.32	1.08± 0.28*	1.08± 0.23	0.845	0.756
FG (mmol·L^-1^)	6.22± 1.78	6.17± 1.78	6.40± 1.40	6.38± 1.76	0.698	0.868
SBP (mm Hg)	132± 17	123± 12	137± 17	127± 14	<0.001	0.371
DBP (mm Hg)	83± 10	78± 7	85± 11	78± 9	<0.001	0.107
WCirc (cm)	101.1± 10.7	98.8± 10.3	109.5± 9.5	107.1± 9.6	<0.001	0.795
MetS Z-score	0.36± 0.57	0.18± 0.56	0.44± 0.49	0.17± 0.49	<0.001	0.098

*Different from women at baseline

### Body weight and composition

Body weight and composition at baseline, and after HIIT are displayed in Table 3. At baseline, body mass index was similar between groups (P = 0.461). However, body weight and fat-free mass were 13.3 kg (P<0.001) and 16.8 kg (P<0.001) higher in men, whereas fat mass was 3.6 kg higher in women (P = 0.018). In overall, only body weight and fat mass improved after the intervention (time ES = 0.144, and ES = 0.042) without differences between groups (time by gender, ES = 0.027, and ES = 0.031).

**Table 3:** Body weight and composition progression after 16 weeks of high intensity interval training. Data are means ± SD.

	Women		Men		P	
	Pre	Post	Pre	Post	Time	Time by Gender
Body weight (kg)	81.0± 12.4	80.6± 12.5	94.3± 15.5*	93.2± 15.4	<0.001	0.075
BMI (kg·m^-2^)	32.8±4.9	32.6±4.9	32.1± 4.4	31.8± 4.4	<0.001	0.173
Fat free mass (kg)	46.7 ± 5.9	46.3 ± 5.9	63.5 ± 8.9*	63.8 ± 10.3	0.999	0.248
Fat mass (kg)	34.4± 7.8	34.3 ± 7.8	30.8 ± 8.5*	29.3± 9.6	0.025	0.054

* Different from women at baseline

### Correlations

Pearson’s coefficients of correlation at baseline are displayed in Table 4. In both, women and men, OUES (P<0.001 in both cases) and MFO (P<0.001, and P = 0.024, respectively), correlated with VO_2PEAK_. However, VE/VCO_2_ slope showed a significant correlation with VO_2MAX_ only in women (P = 0.017). MetS Z-score correlated in both, women and men with body weight (P<0.001, and P = 0.005, respectively), whereas, only in women with OUES (P = 0.010). Pearson’s coefficients of correlation from changes after exercise are displayed in Table 5. Changes in VO_2PEAK_ correlated with both, women and men only with OUES (P<0.001 and P = 0.012, respectively), whereas MFO changes correlated with VO_2MAX_ changes only in women (P = 0.013). MetS Z-score changes correlated in women and men, only with changes in body weight (P = 0.003, and P = 0.027).

**Table 4:** Pearson´s correlation coefficient of studied variables at baseline.

	VO_2PEAK_ (L·min^-1^)	VO2@VT1 (L·min-1)	OUES	VEVCO_2_ SLOPE	MFO (g·min^-1^)	BW (kg)	MetS Z-score
**WOMEN**							
VO_2PEAK_ (L·min^-1^)	1	0.569*	0.726*	-0.297*	0.456*	0.347*	0.046
VO2@VT1 (L·min^-1^)		1	0.458*	-0.206	0.397*	0.328*	0.109
OUES			1	-0.661*	0.426*	0.350*	0.323*
VEVCO_2_ slope				1	-0.096	-0.308*	-0.236
MFO (g·min^-1^)					1	,340**	0.160
BW (kg)						1	0.436*
MetS Z-score							1
**MEN**							
VO_2PEAK_ (L·min^-1^)	1	0.680*	0.658*	0.003	0.301*	0.390*	-0.070
VO2@VT1 (L·min^-1^)		1	0.460*	0.055	0.348*	0.487*	-0.059
OUES			1	0.482*	0.318*	0.466*	0.186
VEVCO_2_ slope				1	-0.089	-0.127	-0.209
MFO (g·min^-1^)					1	0.790	-0.028
BW (kg)						1	0.383*
MetS Z-score							1

* Significant correlation at P<0.05

**Table 5:** Pearson´s correlation coefficient of changes from studied variables.

	VO_2PEAK_ (L·min^-1^)	VO2@VT1 (L·min-1)	OUES	VEVCO_2_ SLOPE	MFO (g·min^-1^)	BW (kg)	MetS Z-score
**WOMEN**							
VO_2PEAK_ (L·min^-1^)	1	0.378*	0.685*	0.101	0.309*	0.080	-0.134
VO2@VT1 (L·min^-1^)		1	0.341*	0.123	0.232	-0.012	0.070
OUES			1	-0.525*	0.346*	0.006	-0.023
VEVCO_2_ slope				1	-0.105	-0.003	-0.038
MFO (g·min^-1^)					1	-0.178	-0.029
BW (kg)						1	0.372*
MetS Z-score							1
**MEN**							
VO_2PEAK_ (L·min^-1^)	1	0.445*	0.330*	-0.028	0.124	0.010	-0.060
VO2@VT1 (L·min^-1^)		1	0.022	0.207	0.404*	-0.148	-0.083
OUES			1	-0.420*	0.211	0.031	-0.248
VEVCO_2_ slope				1	-0.058	0.075	0.065
MFO (g·min^-1^)					1	-0.174	-0.198
BW (kg)						1	0.300*
MetS Z-score							1

* Significant correlation at P<0.05

## Discussion

We recruited 56 men and 63women with the same initial state of metabolic syndrome (i.e., MetS Z-score) to participate in a supervised exercise program of 16 weeks, aiming to compare heath-related training adaptations differenced by gender. We hypothesized that women’s lower capacity to generate muscle power during exercise would reflect in lesser cardiometabolic benefit from HIIT. Our main finding was that despite baseline differences in some variables, none of the measured variables showed gender-related differential progression. There were no gender differences in the improvement of MetS Z-score which was driven by lowering in blood pressure and waist circumference (Table 2). Likewise, there was a similar increase in CRF (i.e., VO_2MAX_, OUES, VE/VCO_2_ slope) and the ability to oxidize fat during exercise (i.e., MFO) between gender.

CRF is associated with the prevalence and cardiovascular complications of MetS. Assessment of CRF provides a complete evaluation of cardiovascular, respiratory and metabolic status and its adaptations after exercise training. VO_2MAX_ values below predicted normative values are associated with adverse cardiovascular events in apparently healthy individuals [21], and patients with cardiovascular disease [42, 43]. At baseline, our participants showed values of VO_2MAX_ slightly under the age and gender predicted normative values (i.e., 97 ± 18%) which increase after training to 111 ± 19%, along with improved MetS Z-score. This suggests that the cardiovascular and metabolic risk factors associated with low CRF in patients with MetS can be reverted with 4 months of HIIT in both, women and men.

In agreement with previous publications [7–12], we found that at baseline, VO_2MAX_ expressed either in absolute or relative values, was higher in men than in women. VO_2MAX_ improvements with training expressed in absolute or relative terms (normalized by body weight or fat-free mass) showed no gender-related differences in agreement with previous studies conducted after HIIT in active and young men and women [16], as well as in sedentary healthy middle-aged adults [17] and older healthy but sedentary adults [44]. Thus, as it was described in a healthy population, in patients diagnosed with metabolic syndrome, men have a higher pre-training CRF than women, but the progression of VO_2MAX_ with training is not different between women and men.

Attainment of a true VO_2MAX_ is particularly difficult in deconditioned individuals [45]. Since unfit individuals fatigue at lower work rates than physically active people, surrogate methods to assess CRF which do not depend on vigorous intensity tolerance, have been developed. Ventilatory threshold, which has been used to track adaptations to endurance training in both, healthy individuals and patients with cardiovascular and metabolic disease [19], showed a similar progression between women and men, despite that absolute values of VO2 at VT were higher in men. Our findings agree with a previous study conducted in aged and healthy volunteers, which found after 9 weeks of HIIT a significant improvement in PO at VT without differences between women and men [44]. Astorino et al, [46] reported that 35.7% of participants showed a meanignful improvement of PO at VT after just 9 sessions of HIIT. Presently, PO at VT improved in 60.3% of women and 64.3% of men after 48 sessions of HIIT. Nevertheless our studies coincide in that PO at VT similarly improves with HIIT in women and men.

Oxygen uptake efficiency slope (OUES) and the slope of the relationship between minute ventilation and carbon dioxide production (VE/VCO_2_ slope), had been used to evaluate cardiopulmonary functional reserve without requiring subjects to reach maximal efforts [18]. In addition, MFO has been associated with cardiorespiratory fitness and it has been considered a surrogate of mitochondrial function [47], linked with the ability to avoid impairment on insulin signaling related with incomplete oxidation of free fatty acids [23].

OUES was higher in men suggesting a better O_2_ delivery and extraction of oxygen during exercise. Baseline OUES correlated positively with VO_2MAX_ [18, 48] in women and men. Our data showed that baseline OUES correlated significantly with VO_2MAX_ being the correlation coefficient higher in women. Likewise, after training, the correlation between improvements in VO_2MAX_ and OUES was better in women (Table 5). We are not the first showing changes in OUES after training. Patients with cardiac heart failure showed OUES improvement after exercise training [49–51]. However, a comparison of OUES changes after HIIT, and their relationship with other CRF parameters is a novel contribution. We observe that this submaximal parameter is highly correlated with VO_2MAX_ improvement at least in women and could be a good candidate to track CRF progression in individuals unable to endure maximal fatiguing exercise.

According to previous studies, VE/VCO_2_ slope increases in relation with age, being mildly lower in men than in women [52]. We found that either at baseline or after training, VE/VCO_2_ was not different between men and women, possibly indicating that the mechanisms of CO_2_ diffusion work similarly in men and women independently of the ability to deliver oxygen. As it was described for VO_2MAX_ and OUES, VE/VCO_2_ slope increased after training. However, increases in the VE/VCO_2_ slope values have been associated with unfavorable prognosis in patients with cardiovascular disease, particularly heart failure [53]. On the other hand, VE/VCO_2_ slope was not useful to discriminate higher vs. lower values of VO_2MAX_ in healthy women with overweight [54]. In addition, Kemps et al. [51], did not find post-intervention differences in VE/VCO_2_ slope between a group of sedentary patients with stable CHF who remained sedentary and a group of individuals with similar characteristics trained with a combination of interval intensity cycling and muscle resistance training. VE/VCO_2_ slope increases after the respiratory compensation point and thus, the longer a subject tolerates exercise intensities above RCP the higher would be the VE/VCO_2_ slope. HIIT training improves tolerance to vigorous intensities and time to exhaustion above the respiratory compensation point [55] where VE/VCO_2_ slope peaks. Further research is warranted to answer this hypothesis.

CRF is a good index of individuals health status and in epidemiological studies it is highly associated with mortality for cardiovascular and overall causes [21]. Independently of the parameter used to track CRF in patients with metabolic syndrome (i.e., VO2max, VT, OUES), we found that women and men similarly improved CRF. It seems that baseline differences in CRF are mainly due to anthropometric characteristics which do not affect the progression of CRF when training is based on target HR. Our data show that despite exercising at different absolute work rates, women and men endured similar cardiovascular and metabolic stress during each exercise session of HIIT which explain the similar CRF adaptations. Thus, when workloads are set as percentages of maximal values of HR, women and men respond similarly since training workloads are similar in relative values. Furthermore, our data suggest that metabolic syndrome *per se* does not impede CRF improvement [2]. Thus, standardized HIIT based on target HR grants similar CRF adaptations in women and men with metabolic syndrome.

MFO rate measured during a submaximal test, a surrogate of mitochondrial activity, is blunted in detrained individuals [56, 57] and patients with insulin resistance and MetS [47, 58]. When MFO is compared between men and women it is crucial to normalize data by fat-free mass (FFM), since in average, women have higher percentage of fat mass and still less size than men. In consequence, absolute values of MFO could mislead the interpretation of findings. For example, according to our data and previous findings [26], MFO in absolute values was greater in men. However, after normalization by FFM we found that MFO was 7% higher in women at baseline, and 4% higher after training, without a significant difference. In fact, most of the authors who have addressed differences in fat oxidation between women and men have found that after normalization, women have higher rates of MFO [29, 59]. At baseline, MFO correlated with VO_2MAX_ and OUES in both, women and men, reflecting the relation between oxidative capacity and cardiorespiratory functional capacity. MFO increased after training similarly in women and men, with independence of baseline values. Correlation of MFO changes with VO_2MAX_ or OUES changes was significant only in women suggesting that the mechanisms behind the improvement of CRF are not exactly the same between women and men. Spina et al [60], found that VO_2MAX_ improvements in men were more related to central adaptations (i.e., cardiac output) whereas in women were led mainly by peripheral adaptations (i.e., oxygen extraction). Therefore, at least in patients with MetS, MFO seems to provide useful information about the baseline oxidative capacity and development of adaptations after training, which according to our data occur similarly in men and women.

Gender-related responses of MetS components after training have been previously studied. Morita et al [3] found in elder men and women with 2 or more CVD risk factors that after 6 months of moderate-intensity continuous exercise training, the reductions in SBP and DBP were higher in women. Likewise, waist circumference reductions tended to be higher in women. On the other hand, Katzmarzyk et al. [4] in the HERITAGE study did not find gender differences in the efficacy of a supervised 22-weeks aerobic exercise program in treating the MetS. Presently, we studied the effects of a 16-week supervised intense exercise training program in well-matched men and women with MetS. Using a robust statistical analysis (split-plot ANOVA) and a large sample (119 individuals), we found improvements in blood pressure and waist circumference that reflected in an overall reduction in MetS after training (i.e., Z score; Table 2). More importantly, we found that the improvements were similar between women and men.

In our study we did not include a control group of women and men assessed with 16 weeks of difference but without a training intervention. Although the lack of control group is an important limitation of studies which study the efficacy of a particular intervention, presently we are testing the progression of different health parameters between men and women with metabolic syndrome, and the relevant comparison is the progression of each variable across time in women and men. Volunteers trained during 4 months, 3 times per week, under the supervision of a research staff member. As a limitation of our study, we do not have records of HR from all participants in every exercise sessions. Although supervision included verbal encouragement to reach and maintain the target HR, it is possible that in some particular intervals some volunteers did not attain the target HR. Since, our main interest was the comparison between men and women, we believe that potential lack of attainment of target HR during training would be symmetrically distributed between women and men, and this would not be a factor to change the overall findings and conclusions. Data from our lab [39], and Okura et al. [61] have shown that adding aerobic exercise training to dietary weight reduction is a more effective treatment for improving MetS than diet alone or exercise alone. In our study, body weight (BW) and waist circumference (WCir) were higher in men but when body weight was normalized by height (i.e., BMI) then the differences between genders disappeared. HIIT elicited a similar reduction in BW, WCir and percent fat in both genders. Thus, it seems that exercise has similar effects in men and women with MetS on improving their body composition. More important, only baseline body weight correlated in both, women and men with MetS Z-score, as well as changes in body weight correlated only with changes in MetS Z-score after training in both women and men. Thus, in agreement with previous publications [62], body weight reductions are more important than CRF improvements to treat MetS.

In our study, participants were advised to maintain their habitual nutritional and physical activity behaviors. Even though, it is possible that some of the participants modified the amount or composition of habitual meals and/or their daily physical activity during the period of training. However, these variations would be distributed between both experimental groups, and in consequence, it would not seem to interfere with gender-related changes in body composition. In addition, we did not conduct direct measurements of cardiac, pulmonary or mitochondrial function to support the finding reported in the variables obtained during the GXT. However, validation of these measurements against direct methods has been previously studied by other groups.

## Conclusions

A 16-week high-intensity interval training program improves cardiorespiratory fitness and MetS components similarly in men and women despite baseline differences. When it is not possible to attain a true VO_2MAX_, VO2 at VT and OUES seems to track correctly VO_2MAX_ baseline values and changes in both women and men. Our data suggest that women with MetS are not at disadvantage to benefit from an exercise program during a HIIT training program, despite that absolute training workloads, and in consequence energy expenditure of each exercise session were higher in men. Finally, it is worth to mention that similarly in women and men, bodyweight reduction showed a stronger association with improvements in MetS *Z*-score than any of the CRF parameters presently analyzed, indicating that bodyweight reduction should be the main focus of lifestyle interventions against metabolic syndrome.

## Supporting information

S1 File TREND statement checklist. (PDF)

S2 File Report for the ethical clinical research committee of Albacete`s Universitary Hospital. (PDF)

S3 File Informe para el Comité Ético de Investigación Clínica del Complejo Hospitalario Universitario de Albacete (Original version). (PDF)

## References

[1] Mozumdar A, Liguori G. Persistent increase of prevalence of metabolic syndrome among U.S. adults: NHANES III to NHANES 1999–2006. Diabetes Care. 2011;34(1):216–9. 10.2337/dc10-0879 . 20889854 PMC3005489

[2] Mora-Rodriguez R, Ortega JF, Hamouti N, Fernandez-Elias VE, Canete Garcia-Prieto J, Guadalupe-Grau A, et al Time-course effects of aerobic interval training and detraining in patients with metabolic syndrome. Nutr Metab Cardiovasc Dis. 2014;24(7):792–8. 10.1016/j.numecd.2014.01.011 . 24656853

[3] Morita N, Okita K. Is gender a factor in the reduction of cardiovascular risks with exercise training? Circ J. 2013;77(3):646–51. 10.1253/circj.cj-12-0607 . 23220798

[4] Katzmarzyk PT, Leon AS, Wilmore JH, Skinner JS, Rao DC, Rankinen T, et al Targeting the metabolic syndrome with exercise: evidence from the HERITAGE Family Study. Med Sci Sports Exerc. 2003;35(10):1703–9. 10.1249/01.MSS.0000089337.73244.9B . 14523308

[5] Lakka TA, Laaksonen DE, Lakka HM, Mannikko N, Niskanen LK, Rauramaa R, et al Sedentary lifestyle, poor cardiorespiratory fitness, and the metabolic syndrome. Med Sci Sports Exerc. 2003;35(8):1279–86. 10.1249/01.MSS.0000079076.74931.9A . 12900679

[6] Hassinen M, Lakka TA, Savonen K, Litmanen H, Kiviaho L, Laaksonen DE, et al Cardiorespiratory fitness as a feature of metabolic syndrome in older men and women: the Dose-Responses to Exercise Training study (DR’s EXTRA). Diabetes care. 2008;31(6):1242–7. 10.2337/dc07-2298 . 18332159

[7] Hossack KF, Bruce RA. Maximal cardiac function in sedentary normal men and women: comparison of age-related changes. J Appl Physiol Respir Environ Exerc Physiol. 1982;53(4):799–804. 10.1152/jappl.1982.53.4.799 . 7153117

[8] Astrand I. Aerobic work capacity in men and women with special reference to age. Acta Physiol Scand Suppl. 1960;49(169):1–92. . 13794892

[9] Martin WH 3rd, Ogawa T, Kohrt WM, Malley MT, Korte E, Kieffer PS, et al Effects of aging, gender, and physical training on peripheral vascular function. Circulation. 1991;84(2):654–64. 10.1161/01.cir.84.2.654 . 1860209

[10] Skinner JS, Jaskolski A, Jaskolska A, Krasnoff J, Gagnon J, Leon AS, et al Age, sex, race, initial fitness, and response to training: the HERITAGE Family Study. Journal of applied physiology. 2001;90(5):1770–6. 10.1152/jappl.2001.90.5.1770 . 11299267

[11] Ogawa T, Spina RJ, Martin WH 3rd, Kohrt WM, Schechtman KB, Holloszy JO, et al Effects of aging, sex, and physical training on cardiovascular responses to exercise. Circulation. 1992;86(2):494–503. 10.1161/01.cir.86.2.494 . 1638717

[12] Franks PW, Ekelund U, Brage S, Wong MY, Wareham NJ. Does the association of habitual physical activity with the metabolic syndrome differ by level of cardiorespiratory fitness? Diabetes care. 2004;27(5):1187–93. 10.2337/diacare.27.5.1187 . 15111543

[13] Woo JS, Derleth C, Stratton JR, Levy WC. The influence of age, gender, and training on exercise efficiency. J Am Coll Cardiol. 2006;47(5):1049–57. 10.1016/j.jacc.2005.09.066 . 16516092

[14] Garber CE, Blissmer B, Deschenes MR, Franklin BA, Lamonte MJ, Lee IM, et al American College of Sports Medicine position stand. Quantity and quality of exercise for developing and maintaining cardiorespiratory, musculoskeletal, and neuromotor fitness in apparently healthy adults: guidance for prescribing exercise. Med Sci Sports Exerc. 2011;43(7):1334–59. 10.1249/MSS.0b013e318213fefb . 21694556

[15] Howden EJ, Perhonen M, Peshock RM, Zhang R, Arbab-Zadeh A, Adams-Huet B, et al Females have a blunted cardiovascular response to one year of intensive supervised endurance training. Journal of applied physiology. 2015;119(1):37–46. 10.1152/japplphysiol.00092.2015 . 25930024 PMC6345209

[16] Astorino TA, Allen RP, Roberson DW, Jurancich M, Lewis R, McCarthy K, et al Adaptations to high-intensity training are independent of gender. European journal of applied physiology. 2011;111(7):1279–86. 10.1007/s00421-010-1741-y . 21132441

[17] Metcalfe RS, Tardif N, Thompson D, Vollaard NB. Changes in aerobic capacity and glycaemic control in response to reduced-exertion high-intensity interval training (REHIT) are not different between sedentary men and women. Applied physiology, nutrition, and metabolism = Physiologie appliquee, nutrition et metabolisme. 2016;41(11):1117–23. 10.1139/apnm-2016-0253 . 27753506

[18] Baba R, Nagashima M, Goto M, Nagano Y, Yokota M, Tauchi N, et al Oxygen uptake efficiency slope: a new index of cardiorespiratory functional reserve derived from the relation between oxygen uptake and minute ventilation during incremental exercise. J Am Coll Cardiol. 1996;28(6):1567–72. 10.1016/s0735-1097(96)00412-3 . 8917273

[19] Meyer T, Lucia A, Earnest CP, Kindermann W. A conceptual framework for performance diagnosis and training prescription from submaximal gas exchange parameters—theory and application. International journal of sports medicine. 2005;26 Suppl 1:S38–48. 10.1055/s-2004-830514 . 15702455

[20] Hansen JE, Sue DY, Wasserman K. Predicted values for clinical exercise testing. The American review of respiratory disease. 1984;129(2 Pt 2):S49–55. 10.1164/arrd.1984.129.2P2.S49 . 6421218

[21] Guazzi M, Arena R, Halle M, Piepoli MF, Myers J, Lavie CJ. 2016 Focused Update: Clinical Recommendations for Cardiopulmonary Exercise Testing Data Assessment in Specific Patient Populations. Circulation. 2016;133(24):e694–711. 10.1161/CIR.0000000000000406 . 27143685

[22] Arena R, Humphrey R. Relationship between ventilatory expired gas and cardiac parameters during symptom-limited exercise testing in patients with heart failure. J Cardiopulm Rehabil. 2001;21(3):130–4. 10.1097/00008483-200105000-00002 . 11409221

[23] Maunder E, Plews DJ, Kilding AE. Contextualising Maximal Fat Oxidation During Exercise: Determinants and Normative Values. Front Physiol. 2018;9:599 10.3389/fphys.2018.00599 . 29875697 PMC5974542

[24] McBride HM, Neuspiel M, Wasiak S. Mitochondria: more than just a powerhouse. Current biology: CB. 2006;16(14):R551–60. 10.1016/j.cub.2006.06.054 . 16860735

[25] Holloszy JO. Regulation of mitochondrial biogenesis and GLUT4 expression by exercise. Comprehensive Physiology. 2011;1(2):921–40. 10.1002/cphy.c100052 . 23737207

[26] Wu YT, Wu SB, Wei YH. Metabolic reprogramming of human cells in response to oxidative stress: implications in the pathophysiology and therapy of mitochondrial diseases. Current pharmaceutical design. 2014;20(35):5510–26. 10.2174/1381612820666140306103401 . 24606797

[27] Wang CH, Wang CC, Wei YH. Mitochondrial dysfunction in insulin insensitivity: implication of mitochondrial role in type 2 diabetes. Ann N Y Acad Sci. 2010;1201:157–65. 10.1111/j.1749-6632.2010.05625.x . 20649552

[28] Haufe S, Engeli S, Budziarek P, Utz W, Schulz-Menger J, Hermsdorf M, et al Determinants of exercise-induced fat oxidation in obese women and men. Hormone and metabolic research = Hormon- und Stoffwechselforschung = Hormones et metabolisme. 2010;42(3):215–21. 10.1055/s-0029-1242745 . 19937568

[29] Venables MC, Achten J, Jeukendrup AE. Determinants of fat oxidation during exercise in healthy men and women: a cross-sectional study. Journal of applied physiology. 2005;98(1):160–7. 10.1152/japplphysiol.00662.2003 . 15333616

[30] Astorino TA, Edmunds RM, Clark A, Gallant R, King L, Ordille GM, et al Change in maximal fat oxidation in response to different regimes of periodized high-intensity interval training (HIIT). European journal of applied physiology. 2017;117(4):745–55. 10.1007/s00421-017-3535-y . 28251399

[31] Alberti KG, Eckel RH, Grundy SM, Zimmet PZ, Cleeman JI, Donato KA, et al Harmonizing the metabolic syndrome: a joint interim statement of the International Diabetes Federation Task Force on Epidemiology and Prevention; National Heart, Lung, and Blood Institute; American Heart Association; World Heart Federation; International Atherosclerosis Society; and International Association for the Study of Obesity. Circulation. 2009;120(16):1640–5. 10.1161/CIRCULATIONAHA.109.192644 . 19805654

[32] Taylor KL, Weston M, Batterham AM. Evaluating intervention fidelity: an example from a high-intensity interval training study. PloS one. 2015;10(4):e0125166 10.1371/journal.pone.0125166 . 25902066 PMC4406743

[33] Dandanell S, Praest CB, Sondergard SD, Skovborg C, Dela F, Larsen S, et al Determination of the exercise intensity that elicits maximal fat oxidation in individuals with obesity. Applied physiology, nutrition, and metabolism = Physiologie appliquee, nutrition et metabolisme. 2017;42(4):405–12. 10.1139/apnm-2016-0518 . 28177732

[34] Sue DY, Wasserman K, Moricca RB, Casaburi R. Metabolic acidosis during exercise in patients with chronic obstructive pulmonary disease. Use of the V-slope method for anaerobic threshold determination. Chest. 1988;94(5):931–8. 10.1378/chest.94.5.931 . 3180897

[35] Anaya SA, Church TS, Blair SN, Myers JN, Earnest CP. Exercise dose-response of the V(E)/VCO(2) slope in postmenopausal women in the DREW study. Med Sci Sports Exerc. 2009;41(5):971–6. 10.1249/MSS.0b013e3181930009 . 19346992

[36] Poole DC, Jones AM. Measurement of the maximum oxygen uptake Vo2max: Vo2peak is no longer acceptable. J Appl Physiol (1985). 2017;122(4):997–1002. 10.1152/japplphysiol.01063.2016 . 28153947

[37] Frayn KN. Calculation of substrate oxidation rates in vivo from gaseous exchange. Journal of applied physiology: respiratory, environmental and exercise physiology. 1983;55(2):628–34. 10.1152/jappl.1983.55.2.628 . 6618956

[38] Jeukendrup AE, Wallis GA. Measurement of substrate oxidation during exercise by means of gas exchange measurements. Int J Sports Med. 2005;26 Suppl 1:S28–37. Epub 2005/02/11. 10.1055/s-2004-830512 . 15702454

[39] Mora-Rodriguez R, Ortega JF, Guio de Prada V, Fernandez-Elias VE, Hamouti N, Morales-Palomo F, et al Effects of Simultaneous or Sequential Weight Loss Diet and Aerobic Interval Training on Metabolic Syndrome. Int J Sports Med. 2015 Epub 2015/12/17. 10.1055/s-0035-1564259 . 26667921

[40] Brage S, Wedderkopp N, Ekelund U, Franks PW, Wareham NJ, Andersen LB, et al Features of the metabolic syndrome are associated with objectively measured physical activity and fitness in Danish children: the European Youth Heart Study (EYHS). Diabetes care. 2004;27(9):2141–8. 10.2337/diacare.27.9.2141 . 15333475

[41] Lakens D. Calculating and reporting effect sizes to facilitate cumulative science: a practical primer for t-tests and ANOVAs. Front Psychol. 2013;4:863 10.3389/fpsyg.2013.00863 . 24324449 PMC3840331

[42] Arena R, Myers J, Abella J, Pinkstaff S, Brubaker P, Moore B, et al Determining the preferred percent-predicted equation for peak oxygen consumption in patients with heart failure. Circ Heart Fail. 2009;2(2):113–20. 10.1161/CIRCHEARTFAILURE.108.834168 . 19808326 PMC2747756

[43] Guazzi M, Adams V, Conraads V, Halle M, Mezzani A, Vanhees L, et al EACPR/AHA Scientific Statement. Clinical recommendations for cardiopulmonary exercise testing data assessment in specific patient populations. Circulation. 2012;126(18):2261–74. 10.1161/CIR.0b013e31826fb946 . 22952317 PMC4777325

[44] Lepretre PM, Vogel T, Brechat PH, Dufour S, Richard R, Kaltenbach G, et al Impact of short-term aerobic interval training on maximal exercise in sedentary aged subjects. International journal of clinical practice. 2009;63(10):1472–8. 10.1111/j.1742-1241.2009.02120.x . 19769704

[45] Dalleck LC, Astorino TA, Erickson RM, McCarthy CM, Beadell AA, Botten BH. Suitability of verification testing to confirm attainment of VO(2)max in middle-aged and older adults. Research in sports medicine. 2012;20(2):118–28. 10.1080/15438627.2012.660825 . 22458828

[46] Astorino TA, deRevere J, Anderson T, Kellogg E, Holstrom P, Ring S, et al Change in VO2max and time trial performance in response to high-intensity interval training prescribed using ventilatory threshold. European journal of applied physiology. 2018;118(9):1811–20. 10.1007/s00421-018-3910-3 . 29923111

[47] San-Millan I, Brooks GA. Assessment of Metabolic Flexibility by Means of Measuring Blood Lactate, Fat, and Carbohydrate Oxidation Responses to Exercise in Professional Endurance Athletes and Less-Fit Individuals. Sports medicine. 2017 10.1007/s40279-017-0751-x . 28623613

[48] Onofre T, Oliver N, Carlos R, Felismino A, Corte RC, Silva E, et al Oxygen uptake efficiency slope as a useful measure of cardiorespiratory fitness in morbidly obese women. PLoS One. 2017;12(4):e0172894 10.1371/journal.pone.0172894 . 28384329 PMC5383027

[49] Gademan MG, Swenne CA, Verwey HF, van de Vooren H, Haest JC, van Exel HJ, et al Exercise training increases oxygen uptake efficiency slope in chronic heart failure. Eur J Cardiovasc Prev Rehabil. 2008;15(2):140–4. 10.1097/HJR.0b013e3282ef19986 . 18391638

[50] Van Laethem C, Van De Veire N, De Backer G, Bihija S, Seghers T, Cambier D, et al Response of the oxygen uptake efficiency slope to exercise training in patients with chronic heart failure. Eur J Heart Fail. 2007;9(6–7):625–9. 10.1016/j.ejheart.2007.01.007 . 17347033

[51] Kemps HM, de Vries WR, Schmikli SL, Zonderland ML, Hoogeveen AR, Thijssen EJ, et al Assessment of the effects of physical training in patients with chronic heart failure: the utility of effort-independent exercise variables. Eur J Appl Physiol. 2010;108(3):469–76. 10.1007/s00421-009-1230-3 . 19834732

[52] Sun XG, Hansen JE, Garatachea N, Storer TW, Wasserman K. Ventilatory efficiency during exercise in healthy subjects. Am J Respir Crit Care Med. 2002;166(11):1443–8. 10.1164/rccm.2202033 . 12450934

[53] Poggio R, Arazi HC, Giorgi M, Miriuka SG. Prediction of severe cardiovascular events by VE/VCO2 slope versus peak VO2 in systolic heart failure: a meta-analysis of the published literature. Am Heart J. 2010;160(6):1004–14. 10.1016/j.ahj.2010.08.037 . 21146651

[54] Keller-Ross ML, Chantigian DP, Evanoff N, Bantle AE, Dengel DR, Chow LS. VE/VCO2 slope in lean and overweight women and its relationship to lean leg mass. International journal of cardiology Heart & vasculature. 2018;21:107–10. 10.1016/j.ijcha.2018.10.009 . 30426069 PMC6222036

[55] Guio de Prada V, Ortega JF, Ramirez-Jimenez M, Morales-Palomo F, Pallares JG, Mora-Rodriguez R. Training intensity relative to ventilatory thresholds determines cardiorespiratory fitness improvements in sedentary adults with obesity. European journal of sport science. 2019;19(4):549–56. 10.1080/17461391.2018.1540659 . 30381027

[56] Holloszy JO, Coyle EF. Adaptations of skeletal muscle to endurance exercise and their metabolic consequences. J Appl Physiol Respir Environ Exerc Physiol. 1984;56(4):831–8. . 6373687 10.1152/jappl.1984.56.4.831

[57] Martin WH 3rd, Dalsky GP, Hurley BF, Matthews DE, Bier DM, Hagberg JM, et al Effect of endurance training on plasma free fatty acid turnover and oxidation during exercise. The American journal of physiology. 1993;265(5 Pt 1):E708–14. . 8238496 10.1152/ajpendo.1993.265.5.E708

[58] Kelley DE, Goodpaster B, Wing RR, Simoneau JA. Skeletal muscle fatty acid metabolism in association with insulin resistance, obesity, and weight loss. The American journal of physiology. 1999;277(6 Pt 1):E1130–41. . 10600804 10.1152/ajpendo.1999.277.6.E1130

[59] Tarnopolsky MA. Gender differences in substrate metabolism during endurance exercise. Canadian journal of applied physiology = Revue canadienne de physiologie appliquee. 2000;25(4):312–27. . 10953068 10.1139/h00-024

[60] Spina RJ, Ogawa T, Martin WH 3rd, Coggan AR, Holloszy JO, Ehsani AA. Exercise training prevents decline in stroke volume during exercise in young healthy subjects. J Appl Physiol (1985). 1992;72(6):2458–62. 10.1152/jappl.1992.72.6.2458 . 1385806

[61] Okura T, Nakata Y, Ohkawara K, Numao S, Katayama Y, Matsuo T, et al Effects of aerobic exercise on metabolic syndrome improvement in response to weight reduction. Obesity (Silver Spring). 2007;15(10):2478–84. 10.1038/oby.2007.294 . 17925474

[62] Mora-Rodriguez R, Ortega JF, Ramirez-Jimenez M, Moreno-Cabanas A, Morales-Palomo F. Insulin sensitivity improvement with exercise training is mediated by body weight loss in subjects with metabolic syndrome. Diabetes & metabolism. 2019 10.1016/j.diabet.2019.05.004 . 31158474

