# Supplementary material for: Women with metabolic syndrome show similar health benefits from high-intensity interval training than men
Source: PLoS One. 2019 Dec 10;14(12):e0225893. doi: 10.1371/journal.pone.0225893 (PMC6903716; doi:10.1371/journal.pone.0225893)
Supplement: S2 File — (PDF) [file pone.0225893.s002.pdf]

REPORT FOR THE ETHICAL CLINICAL RESEARCH COMMITTEE OF ALBACETE`S UNIVERSITARY HOSPITAL

Title of the study “Effects of 16 weeks of combined aerobic-strength training on the individual components of the Metabolic Syndrome; temporary evolution of the improvements ”

Research team:

• Dr. Ricardo Mora Rodríguez (Project Manager). University Professor, School of Nursing and Physiotherapy; Faculty of Sports Sciences. University of Castilla-La Mancha (UCLM)

• Juan Fernando Ortega Fonseca. Graduate in medicine and surgery. Specialty in Sports Medicine. El Bosque University (Colombia).

• Ignacio Ara Royo. University holder. Faculty of Sports Sciences. University of Castilla-La Mancha (UCLM).

• Vicente Martínez Vizcaino. University School Professor. Nursing school. University of Castilla-La Mancha (UCLM).

• Jorn Helge. Senior Professor-Researcher, Dept. Biomedical Sciences. University of Copenhagen

Background

Metabolic syndrome

The term "metabolic syndrome" identifies multiple risk factors for the development of cardiovascular disease and includes the coexistence of central adiposity, dyslipidemia, arterial hypertension and carbohydrate metabolism disorders (Reaven 1988). The metabolic syndrome is also called insulin resistance syndrome, since insulin resistance and compensatory hyperinsulinemia are considered as its main components (Reaven 1995). Waist circumference has been established as the main inclusion criterion according to the definition established by the International Diabetes Federation (Alberti et al. 2006). Metabolic syndrome prevalence in western societies is greater than 20% in the adult population (Hildrum et al. 2007). In reference to the Spanish population, it has been estimated that the metabolic syndrome is suffered by 10% of the population of working age (Sánchez-Chaparro et al. 2008). The prevalence of metabolic syndrome increases with age and with weight gain. Given that the Spanish population is aging (INE 2006), and that there is a tendency among people between 30-50 years to gain body fat, it is expected that metabolic syndrome prevalence will increase in the near future.

Metabolic syndrome increases the risk for cardiovascular disease (particularly coronary heart disease), type 2 diabetes and mortality related to these conditions (Gami et al. 2007; Wilson et al. 2005). In the United States of America, it has been estimated that metabolic syndrome will surpass tobacco consumption as the main risk factor for the development of cardiovascular disease (Deen 2004). The physiological adaptations that derive from physical training have the

potential to decrease the spread of this syndrome among the population. Therefore, we consider that from the social point of view, the chronology of exercise-related adaptations is of great importance. This information could increase the effectiveness of the prescription of exercise programs in the clinical setting (supervised training) and extra-clinical (fitness centers).

Etiology of metabolic syndrome and training effects

In 1992, the American Heart Association included physical inactivity as one of the main factors for coronary heart disease development, with an importance similar to that of other risk factors such as hypercholesterolemia, arterial hypertension and smoking. Unlike the other risk factors, physical inactivity is especially relevant, taking into account that there is a significant percentage of the population that does not perform physical activity compared to those who consume tobacco (27%) or have hypercholesterolemia (29%). Among the Spanish population over 16 years old, 80% work while sitting or standing (physical inactivity) and 61% declare not to participate in physical activity during their free time (INE 2006). On the other hand, pathophysiology of the metabolic syndrome suggests that regular physical exercise may be a potent stimulus to reduce the prevalence of metabolic syndrome. Some of the most relevant adaptations produced by training improve the components of the metabolic syndrome.

Physical training of adequate intensity and duration produces metabolic adaptations that cause increased lipid oxidation and result in body fat reduction, improvement in lipid profile, glucose metabolism and cardiovascular health.

To our knowledge, it is currently not completely known if the metabolic syndrome produces a disorder in the molecular system responsible for the adaptations derived from training. The hypothesis we propose regarding the temporal process in which the events generated by the metabolic syndrome occur is set out below. Abdominal obesity is related with increase blood lipids concentration, through not fully understood mechanisms, generates insulin resistance (Lillioja et al. 1985). Likewise, insulin resistance generates hyperglycemia, which in combination with an unfavorable lipid profile (increased levels of triglycerides and low density lipoproteins-LDL) leads to cardiovascular disease. Cardiovascular disease probably has its origin at the endothelial level as a result of cellular dysfunction (i.e. oxidative stress) that results in vascular damage and atheroma (Lopez-Candales 2001).

Effects of exercise on insulin resistance

Underlying metabolic syndrome, insulin resistance prevails, usually linked to increased abdominal adiposity (Reaven 2006). The molecular basis of the mechanisms behind insulin resistance are not fully understood. Abnormalities in the receptors and / or post-receptor processes have been previously identified and analyzed, as well as the different responses among the various body tissues depending on their loss of sensitivity to the action of insulin (Kashyap and De Fronzo 2007). Although almost 50% of the etiology of insulin resistance can be attributed to genetics, the remaining 50% is related to the level of adiposity and circulating fatty acids in the blood (Lillioja et al. 1985). A strong relationship between training level and insulin sensitivity has been identified.

Acute exercise increases insulin sensitivity to induce glucose uptake by the muscle. Acute effects of physical exercise seem to be related to changes in insulin signaling in response to muscle contraction, such as increased translocation of glucose transporters (GLUT4) to the surface of the cell (Ren et al. 1994).

However, the effects of a single exercise session (60 minutes of continuous pedaling) on insulin action are short-lived (up to 48 hours (Mikines et al. 1988b)) which explains the recommendation to exercise frequently (i.e., every two days). In the long term, training modifies the use of energy substrates by increasing lipid oxidation that can lead to the loss of fat mass. Reduction of body fat is clearly a mechanism through which exercise improves insulin sensitivity over time. Training also increases GLUT4 content, glycogen synthetase activity, mitochondrial enzyme activity and muscle density, improves endothelial function and alters muscle fibers type (Venables and Jeukendrup 2008).

Effects of exercise on hypertension

Obesity is an independent risk factor for the development of high blood pressure. Aerobic training can reduce systolic and diastolic blood pressure (i.e. 14%), especially in patients with moderate or borderline hypertension. The mechanisms proposed as responsible for this effect include reduced sympathetic nerve activity, reduced peripheral resistance to blood flow, and modifications on renal function, particularly increased sodium excretion (McArdle et al. 1996). However, reductions in blood pressure in relation to training do not always occur in the long term. Even so, even if exercise is not effective for blood pressure normalization, it generates health benefits that overcome the lack of a hypotensive effect. As proof of this, the increase in mortality associated with hypertension is eliminated when physical fitness is increased (McArdle et al. 1996).

Recent studies have reported that training at an intensity between 40-70% of VO2max has the same or even better effects than high intensity training, in relation to the reduction in blood pressure. It seems that moderate physical training can cause the same reductions in blood pressure as high intensity exercise but without the possible risks associated with the practice of high intensity exercise (i.e. fatigue, acute myocardial infarction, musculoskeletal injuries). Regarding the clinical course of the relationship between exercise and blood pressure, most studies have shown that blood pressure is reduced early (between 3 weeks and 3 months) after training begins. It has also been found that the systolic in diastolic blood pressure is related to the duration of training while the reduction in diastolic blood pressure does not. Therefore, it seems that blood pressure can be reduced early at the beginning of aerobic training; prolonging such training more than 3 months could result in greater declines (Position stand 1993).

Effects of exercise on hyperlipidemia

Cholesterol and triglycerides are two common types of lipids associated with coronary heart disease, which are transported in the bloodstream combined with proteins forming lipoproteins.

It is believed that during transport of low density lipoproteins (LDL; composed of 45% cholesterol and 10% triglycerides) and very low density lipoproteins (VLDL; 20% cholesterol and 70% triglycerides), cholesterol molecules are released into the vessels. It has been proposed as a mechanism behind the genesis of atherosclerosis. On the other hand, high density lipoproteins (HDL; 18% cholesterol and 2% triglycerides) promote the uptake of cholesterol from peripheral tissues (including arterial walls) to the liver for bile synthesis. There is evidence showing a causal association between the reduction of HDL and the increased risk of coronary heart disease (Caspersen 1987). Periodic physical exercise has a slight effect on LDL levels compared to that generated by other interventions that result in changes in body fat, such as reduction in total dietary fat or cholesterol

However, HDL levels increase in sedentary women and men of all ages when they start aerobic exercise of moderate and / or vigorous intensity (Durstine and Haskell 1994). Favorable changes in lipid profile associated with aerobic exercise occur regardless of changes in body mass. This is one of the bases for the recent recommendations of exercise programs for overweight people, beneficial even if they do not generate weight loss (i.e. fit but fat(Blair and Brodney 1999)). It seems that these favorable changes in the lipid profile with regular physical exercise are related to the greater capacity to reduce the level of blood triglycerides (Sady et al. 1988). Absolute values of HDL appear to be partly genetically determined. Finally, programs that include strength training seem to have little or no effect on blood lipoproteins. It is worth to mention that the exercise increases the plasma volume which produces a reduction in the concentration of HDL and other lipoproteins, for this reason the ratio of total cholesterol / HDL-c seems to be a better index to evaluate the benefits of training on lipid profile.

hypocaloric diet and exercise are the two most used strategies to reduce weight. It is not fully established (Wu et al. 2009) if exercise plus calorie restrictions are more effective than the achieving the same caloric balance by diet alone. A meta-analysis that included only randomized clinical studies concluded that the addition of physical exercise to other interventions designed to reduce weight, improved various health-related variables such as lipid profile, blood pressure, insulin sensitivity and psychic well-being , regardless of the effect on body fat (Kiernan et al. 2001). Less is known about the effects of training in reducing the components of the metabolic syndrome. In one of the studies that included an intervention in patients with metabolic syndrome (Katzmarzyk et al. 2003) it was shown that despite no significant weight loss, 31% of participants ceased to be classified with metabolic syndrome after 22 weeks of training.

Maximum fat oxidation as a method to evaluate the effects of exercise on metabolic syndrome

The term "metabolic fitness" is currently recognized in the scientific literature. Despite not being well defined yet, it is associated with positive changes in lipid profile, blood pressure and insulin sensitivity (Tremblay et al. 1999). The closer to the normal physiological values f a healthy individual, the better the metabolic fitness. A new variable that is being measured for the study of metabolic fitness is the ability to oxidize fat during exercise (Helge et al. 2008). This variable has arisen when observing that during a period of training at moderate intensity (exercise for health) sometimes there are no increases in maximum oxygen consumption (Bouchard et al. 1992). This is the case of the study by Helge et al. (Helge et al. 2008) where the maximum oxygen consumption

did not change after 33 days training cross-country skiing while its maximum capacity to oxidize fat increased significantly.

Therefore, the maximum oxidation of fat during exercise and how it moves towards lower intensities with training gives an idea of the metabolic flexibility (i.e. adaptability) of the system due to the influence of physical training. This index has been studied successfully in works that included obese and type 2 diabetics (Ara et al. 2011; Larsen et al. 2009). It is not yet clear whether subjects with any metabolic abnormality (i.e., obese and / or patients with metabolic syndrome) have a reduced ability to oxidize fats during exercise (Ara et al. 2011; Larsen et al. 2009) and if the training could normalize the ability to oxidize fats during exercise in this population. In this project, we will use for the first time the maximum oxidation of fats during exercise in patients with metabolic syndrome as an index of metabolic adaptations derived from an exercise program.

Duration and type of exercise for the treatment of metabolic syndrome

It is recognized that aerobic exercise may be beneficial for treating metabolic syndrome in men and women. Some studies report up to 35% of therapeutic success (Katzmarzyk et al. 2003), using a 20-week training. However, the optimal duration of an exercise program to achieve benefits in each of the components of the metabolic syndrome has not yet been established and this is one of the central objectives of the present study. According to Durstine et al. (Durstine et al. 2002) there is practically no change in the levels of HDL-c related to exercise in a program shorter than 12 weeks but when said program extends beyond this time, the Increases in the HDL are more likely.

From a meta-analysis where the effects of exercise in relation to weight loss were analyzed (Boule et al. 2001), it can be concluded that exercise programs designed to achieve significant weight loss should have at least 12 weeks. One of the included studies (Dunstan et al. 1998) in the mentioned meta-analysis reported changes in the area under the insulin curve after 8 weeks of circuit weight training, without changes in blood glucose and insulin levels in fasting or glycosylated hemoglobin (ie, HbA1c). In 2010, a study reported that a 12-week training (120 minutes of exercise per week), in patients with metabolic syndrome did not produce a reduction in body weight and as a result, patients did not improve their glucose metabolism (Stensvold et al. 2010). However, participants reduced their waist circumference and improved their endothelial function measured by dilation related to flow. On the other hand, other studies have not been able to document effects on endothelial function within 24 weeks of a change in lifestyle that included exercise in patients with metabolic syndrome (Aizawa et al. 2009).

In summary, it is not entirely clear what type of exercise (aerobic training or strength training) and what should be the duration of a training program aimed to improve the components of metabolic syndrome . It seems that a regimen lasting more than 12 weeks and with a frequency greater than 120 minutes per week is necessary for some of the components of the metabolic syndrome to be modified. Moreover, it seems that the combination of a combined training program (aerobic and strength) is as effective as only aerobic training (Stensvold et al. 2010). Taking into account the previously cited information, the design of this study arises, which will be detailed in the methods section.

References

Aizawa K, Shoemaker JK, Overend TJ, Petrella RJ (2009) Metabolic syndrome, endothelial function and lifestyle modification. Diab Vasc Dis Res 6: 181-189

Alberti KG, Zimmet P, Shaw J (2006) Metabolic syndrome-a new world-wide definition. A consensus statement from the International Diabetes Federation. Diabet Med 23: 469-480

Ara I, Larsen S, Stallknecht B, Guerra B, Morales-Alamo D, Andersen JL, Ponce-Gonzalez JG, Guadalupe-Grau A, Galbo H, Calbet JAL, Helge JW (2011) Normal mitochondrial function and increased fat oxidation capacity in leg and arm muscles in obese humans. International Journal of Obesity , 35: 99-108

Ballor DL, Katch VL, Becque MD, Marks CR (1988) Resistance weight training during caloric restriction enhances lean body weight maintenance. Am J Clin Nutr 47: 19-25

Bergstrom J (1962) Muscle electrolytes in man. Scand J Clin Lab Invest [Suppl] 14: 1-110

Blair SN, Brodney S (1999) Effects of physical inactivity and obesity on morbidity and mortality: current evidence and research issues. Med Sci Sports Exerc 31: S646-662

Bouchard C, Dionne FT, Simoneau JA, Boulay MR (1992) Genetics of aerobic and anaerobic performances. Exerc Sport Sci Rev 20: 27-58

Boule NG, Haddad E, Kenny GP, Wells GA, Sigal RJ (2001) Effects of exercise on glycemic control and body mass in type 2 diabetes mellitus: a meta-analysis of controlled clinical trials. JAMA 286: 1218-1227

Caspersen CJ (1987) Physical inactivity and coronary heart disease. Physician and Sportsmedicine 15: 43-44

Deen D (2004) Metabolic Syndrome: Time for Action. Am Fam Physician 69: 2887-2888

Dunstan DW, Puddey IB, Beilin LJ, Burke V, Morton AR, Stanton KG (1998) Effects of a short-term circuit weight training program on glycaemic control in NIDDM. Diabetes Res Clin Pract 40: 53-61

Durstine JL, Grandjean PW, Cox CA, Thompson PD (2002) Lipids, lipoproteins, exercise. J Cardiopulm Rehabil 22: 385-398

Durstine JL, Haskell WL (1994) Effects of exercise training on plasma lipids and lipoproteins. Exer Sport Sci Rev 22: 447

Frayn KN (1983) Calculation of substrate oxidation rates in vivo from gas exchange. J Appl Physiol 55: 628-634

Frayn KN, Maycock PF (1980) Skeletal muscle triaglycerol in the rat: methods for sampling and measurement, and studies of biological variability. J Lipid Res 21: 139-144

Gami AS, Witt BJ, Howard DE, Erwin PJ, Gami LA, Somers VK, Montori VM (2007) Metabolic syndrome and risk of incident cardiovascular events and death: a systematic review and meta-analysis of longitudinal studies. J Am Coll Cardiol 49: 403-414

Helge JW, Damsgaard R, Overgaard K, Andersen JL, Donsmark M, Dyrskog SE, Hermansen K, Saltin B, Daugaard JR (2008) Low-intensity training dissociates metabolic from aerobic fitness. Scand J Med Sci Sports 18: 86-94

Hildrum B, Mykletun A, Hole T, Midthjell K, Dahl A (2007) Age-specific prevalence of the metabolic syndrome defined by the International Diabetes Federation and the National Cholesterol Education Program: the Norwegian HUNT 2 study. BMC Public Health 7: 220

Horowitz JF, Mora-Rodriguez R, Byerley LO, Coyle EF (1997) Lipolytic suppression following carbohydrate ingestion limits fat oxidation during exercise. Am J Physiol 273: E768-775

Horowitz JF, Mora-Rodriguez R, Byerley LO, Coyle EF (1999) Substrate metabolism when subjects are fed carbohydrate during exercise. Am J Physiol 276: E828-835.

Hwang LC, Bai CH, Chen CJ, Chien KL (2007) Gender difference on the development of metabolic syndrome: a population-based study in Taiwan. Eur J Epidemiol 22: 899-906

INE (2006) Encuesta Nacional de Salud; Instituto Nacional de Estadística.

Jackson AS, Pollock ML (1978) Generalized equations for predicting body density of men. Br J Nutr 40: 497-504

Jackson AS, Pollock ML, Ward A (1980) Generalized equations for predicting body density of women. Med Sci Sports Exerc 12: 175-181

Kashyap SR, De Fronzo RA (2007) The insulin resistance syndrome: physiological considerations. Diab Vasc Dis Res 4: 13-19

Katzmarzyk PT, Leon AS, Wilmore JH, Skinner JS, Rao DC, Rankinen T, Bouchard C (2003) Targeting the metabolic syndrome with exercise: evidence from the HERITAGE Family Study. Med Sci Sports Exerc 35: 1703-1709

Kiernan M, King AC, Stefanick ML, Killen JD (2001) Men gain additional psychological benefits by adding exercise to a weightloss program. Obes Res 9: 770-777

Larsen S, Ara I, Rabøl R, Andersen JL, Boushel R, Dela F, Helge JW (2009) Are substrate use during exercise and mitocondrial respiratory capacity decreased in arm and leg muscle in type 2 diabetes? Diabetologia 52: 1400-1408

Lillioja S, Bogardus C, Mott DM, Kennedy AL, Knowler WC, Howard BV (1985) Relationship between insulin-mediated glucose disposal and lipid metabolism in man. J Clin Invest 75: 1106-1115

Lopez-Candales A (2001) Metabolic syndrome X: a comprehensive review of the pathophysiology and recommended therapy. J Med 32: 283-300

McArdle WD, Katch FI, Katch VL (1996) Physical activity, health and aging. In: Balado D (ed) Exercise Physiology; energy, nutrition and human performance. Williams and Wilkins, Baltimore, pp. 635-655

Mikines K, Sonne B, Farrell P, Tronier B, Galbo H (1988a) Effect of physical exercise on sensitivity and responsiveness to insulin in humans. Am J Physiol 254: E248-E259

Mikines KJ, Sonne B, Farrell PA, Tronier B, Galbo H (1988b) Effect of physical exercise on sensitivity and responsiveness to insulin in humans. Am J Physiol 254: E248-259

Milesis CA, Pollock ML, Bah MD, Ayres JJ, Ward A, Linnerud AC (1976) Effects of different durations of physical training on cardiorespiratory function, body composition, and serum lipids. Res Q 47: 716-725

Paniagua JA, de la Sacristana AG, Romero I, Vidal-Puig A, Latre JM, Sanchez E, Perez-Martinez P, Lopez-Miranda J, Perez-Jimenez F (2007) Monounsaturated Fat-Rich Diet Prevents Central Body Fat Distribution and Decreases Postprandial Adiponectin Expression Induced by a Carbohydrate-Rich Diet in Insulin-Resistant Subjects. Diabetes Care %R 102337/dc06-2220 30: 1717-1723

Passonneau JV, Lauderdale VR (1974) A comparison of three methods of glycogen measurement in tissues. Anal Biochem 60: 405-412

Perseghin G, Price TB, Petersen KF, Roden M, Cline GW, Gerow K, Rothman DL, Shulman GI (1996) Increased Glucose Transport-Phosphorylation and Muscle Glycogen Synthesis after Exercise Training in Insulin-Resistant Subjects. N Engl J Med 335: 1357-1362

Position stand (1993) Physical activity, physical fitness, and hypertension. Med Sci Sports Exerc 25: i-x

Reaven G (2006) The metabolic syndrome: is this diagnosis necessary? Am J Clin Nutr 83: 1237-1247

Reaven GM (1988) Role of insulin resistance in human disease. Diabetes 1595-1607

Reaven GM (1995) Characteristics of metabolic syndrome. Endocrinol Metab 2: 37-42

Ren JM, Semenkovic CF, Gulve EA, Gao J, Holloszy JO (1994) Exercise induces rapid increases in GLUT4 expression, glucose transport capacity, and insulin-stimulated glycogen storage in muscle J Biol Chem 269: 14396-14401

Sady SP, Cullinane EM, Saritelli A, Bernier D, Thompson PD (1988) Elevated high-density lipoprotein cholesterol in endurance athletes is related to enhanced plasma triglyceride clearance. Metabolism 37: 568-572

Sánchez-Chaparro M, Calvo-Bonacho E, González-Quintela A, Fernández-Labandera C, Cabrera M, Sáinz J, Fernández-Meseguer A, Banegas J, Ruilope L, Valdivielso P, Román-García J, Group ICRAIS (2008) Occupation-related differences in the prevalence of metabolic syndrome. Diabetes Care 31: 1884-1885

Stensvold D, Tjønna AE, Skaug EA, Aspenes S, Stølren T, Wisløff U, Slørdahl SA (2010) Strength training versus aerobic interval training to modify risk factors of metabolic syndrome. J Appl Physiol 108: 804-810

Tremblay A, Doucet E, Imbeault P, Mauriège P, Després JP, Richard D (1999) Metabolic fitness in active reduced-obese individuals. Obes Res 7: 556-563

Tremblay A, Fontaine E, Poehlman ET, Mitchell D, Perron L, Bouchard C (1986) The effect of exercise-training on resting metabolic rate in lean and moderately obese individuals. Int J Obes 10: 511-517

Tura A, Sbrignadello S, Succurro E, Groop L, Sesti G, Pacini G An empirical index of insulin sensitivity from short IVGTT: validation against the minimal model and glucose clamp indices in patients with different clinical characteristics. Diabetologia 53: 144-152

Venables MC, Jeukendrup AE (2008) Endurance training and obesity: effect on substrate metabolism and insulin sensitivity. Med Sci Sports Exerc 40: 495-502

Wilson PW, D'Agostino RB, Parise H, Sullivan L, Meigs J (2005) Metabolic syndrome as a precursor of cardiovascular disease and type 2 diabetes mellitus. Circulation 112: 3066-3072

Wu T, Gao X, Chen M, van Dam RM (2009) Long-term effectiveness of diet-plus-exercise interventions vs. diet-only interventions for weight loss: a meta-analysis. Obes Rev 10: 313-323

Zuti WB, Holding LA (1976) Comparing diet and exercise as weight reduction tools. Physician and Sportsmedicine 4: 49-53

PURPOSE OF THE PROJECT, JUSTIFICATION.

We intend that the results of this study would be applicable to regional and national health policies to prevent metabolic syndrome, clearly associated with morbidity and mortality due to cardiovascular disease and diabetes mellitus. The results can be applied to prevent or mitigate the development of insulin resistance in overweight adults through behavioral changes such as incorporating physical exercise into lifestyle.

Objectives

The main objective is to determine the effect of continued and structured exercise (physical training) on the four components (abdominal obesity, dyslipidemia, insulin resistance and hypertension) of the metabolic syndrome.

Specific objetctives

1. Identify on a temporary scale for 4 months, the effects of physical training on the reversal of each of the components of the metabolic syndrome in both, women and men, looking for sex-related differences in the response of: i) central (abdominal) obesity, ii) atherogenic dyslipidemia, iii) disorder in the metabolism of carbohydrates and iv) vascular dysfunction.

2. Evaluate the recurrence rate (recurrence) of each of the components of the metabolic syndrome up to 6 months after having suspended the supervised exercise program.

3. Evaluate whether the metabolic syndrome is constituted as a factor that limits or delays some of the adaptations to exercise training observed in a group of people matched by age, gender, physical activity, body composition and cardiorespiratory fitness. (i.e., evaluated according to the maximum oxygen consumption VO2max).

4. Determine if the weight loss achieved through 4 months through supervised exercise is more effective in the treatment of metabolic syndrome, than that obtained by caloric restriction without exercise.

5. Determine the adaptations that cause 4 months of exercise-based intervention in metabolic fitness (i.e. maximum fat oxidation)

Hypothesis

• Our main hypothesis is that considering that in most people the different components of the metabolic syndrome (abdominal obesity, dyslipidemia, insulin resistance and hypertension) appear in an orderly manner (Hwang et al. 2007), of the in the same way they will disappear neatly in relation to the adaptations derived from physical training.

• Likewise, the progression of the reappearance of the components will be ordered and this will give us clues about which are the most modifiable components to be able to influence them.

• Additionally we hypothesize that for a similar weight loss, achieved by a caloric restriction or secondary to an increase in energy expenditure through exercise, the latter will be more effective in the treatment of metabolic syndrome.

METHODS

Experimental study (randomized)

Recruitment strategy

Participants of this research will be recruited through advertisements in local media (newspapers and radio), through which overweight volunteers will be requested according to body mass index (BMI <25). From this large group of potential participants who respond to the announcements, 60 sedentary men and women between the ages of 25 and 65 who have criteria for Metabolic Syndrome (SinMet) will be recruited. Additionally, 60 sedentary people will be recruited but they do not meet criteria for SinMet (Controls) who will be matched with the participants of the SinMet group by gender, age, waist circumference, body composition and cardiorespiratory physical fitness (assessed by an incremental exercise test and VO2 peak peak oxygen consumption estimate)

Inclusion and exclusion criteria

Metabolic syndrome will be defined according to the criteria published by the International Diabetes Federation: waist circumference> 94 cm for men and> 80 for women of Caucasian ethnicity, plus two of the following 3 factors: high blood pressure figures (> 130 mmHg for systolic and> 85 for diastolic), high fasting blood glucose (> 100 mg / dl), dyslipidemia: (triglycerides> 150 mg / dl, HDL <40 mg / dl) (Alberti, Zimmet et al. 2006). Participants who are being treated for hypertension or who receive hypoglycemic or lipid lowering medications will be considered as having the respective inclusion factor. All subjects must be physically inactive, at least during the previous year. Additionally, they should not have a diagnosis of any endocrine pathology other than carbohydrate intolerance and their hormonal levels (except insulin) should be in ranges of clinical normality. Other exclusion criteria will be: recent surgery, cardiovascular disease (especially coronary heart disease, heart valve disease, heart failure, complex ventricular arrhythmias, diseases with renal, hepatic, respiratory or neuromuscular involvement. Participants will be informed verbally and in writing of the risks and benefits related to participation in the study and will provide their written consent. Participants will maintain supervision by their family doctors who will adjust their treatments according to the particular needs of each individual; such modifications will be informed to the research group.

Reasons not to exclude women in the study

There are not enough studies that have evaluated the gender-related differences in relation to the development and treatment of metabolic syndrome. Despite the similar prevalence between genders when studying individuals of the same age, men usually show a higher percentage of hyperglycemia, hypertension and hypertriglyceridemia. In contrast, women usually show a higher prevalence of central obesity and low concentrations of HDL-c (Bo, Gentile et al. 2005). Moreover, it seems that women tend to isolate the first component before and present with the metabolic

syndrome later than men (Hwang, Bai et al. 2007). In the same way that there are differences between men and women in the development of metabolic syndrome, the time scale in which exercise can correct the defects associated with the components of the metabolic syndrome can also differ between men and women. Data from the Fels study (Remsberg, Rogers et al. 2007) show that the total increase in sports and physical activity reduced the components of the metabolic syndrome in men but not so clearly in women. However, Katzmarzyk et al. argue the opposite, (Katzmarzyk, Leon et al. 2003). We believe that there is insufficient data to conclude that women with metabolic syndrome will respond differently to training than their male peers. For this reason we have decided to include participants of both genders. This fact will allow us to analyze later if there are gender differences as a result of the training programs.

Allocation of participants and calculation of sample size.

Participants will be stratified by age and medication received. According to the inclusion criteria previously described, they will be assigned to the metabolic syndrome group or control group. Each of the two groups will be randomly assigned to one of two interventions: Physical training or Caloric restriction with sedentary lifestyle. As a result of the above there will be 4 groups identified with their respective acronyms (i.e., MetSyn-Ex; MetSyn-Cr; Con-Ex; Con-Cr (table 1.)). We have included caloric restriction groups in an attempt to achieve weight loss achieved by the subjects of the exercise groups in the group of participants who will remain sedentary, since weight loss is a confounding factor in the analysis. .

Each of the groups (MetSyn-Ex; MetSyn-Cr; Con-Ex; Con-Cr) will consist of 30 subjects taking into account that the power analysis of a study in which similar exercise programs were used (3 months duration) in similar subjects (Stensvold, Tjønna et al. 2010) indicates that in order to observe significant effects on the components of the metabolic syndrome, 27 participants in each group were necessary, with which we propose to recruit 30 to have a 10% lack of adherence to the exercise or diet program (Vincent 1999).

Table 1. Experimental groups

CONTROL GROUP (n=60)		METSYN GROUP (n=60)	
EXERCISE TRAINING	DIET	EXERCISE TRAINING	DIET
**Con-Ex** (n=30)	**Con-Cr** (n=30)	**MetSyn-Ex** (n=30)	**MetSyn-Cr** (n=30)

Intervention with physical training

The training program will consist of 3 weekly sessions of interval aerobic exercise (EIA) and 1 weekly session of strength training (EF). EIA will consist of a work on cycle-ergometer (or race on a treadmill) that includes a period of 10 min of heating at 70% of the FCpico followed by 5 intervals of 4 minutes at 90-95% of your FCpico interspersed with periods of 3 minutes of active recovery at 70% of the FCpico, with a cooling of 10 minutes, reaching a total of 55 min of duration by training. Taking into account the effects of the circadian rhythm on heart rate, workouts will be carried out at the same time of day and the load will be adjusted daily to cause the indicated HR. As a consequence of the adaptations produced by the exercise, the training load must be adjusted to reach similar heart rates over time (Lucía, Hoyos et al. 2000) and thus comply with the principle of physical training overload .

The EF sessions will consist of weekly strengthening sessions focused on upper limbs, trunk and lower limbs. Participants will perform a brief warm-up by carrying out exercises to strengthen the abdominal and back area for 10 min. During the first week each exercise will be executed at 60% of the maximum individual repetition (i.e. 1-RM) and three sets of 8 to 12 repetitions will be scheduled in each session. From the second week until the end of the study the volume will be maintained but the intensity will be increased up to 80% of 1-RM. Each month, 1-RM will be evaluated and loads re-adjusted. The circuit will include 3 stations for the upper train (bench press, military shoulder press and rowing in the sitting position) and 3 exercises for the lower train (squat, leg extension and leg curl sitting). The number of repetitions multiplied by the weight will be collected and stored for the evaluation of training adaptations. The total time of the training sessions outside will be approximately 50 min. The total training time per week will be 4 exercise sessions (3 of EIA and 1 of EF) that will add approximately 210 min of moderate-vigorous physical activity.

Nutritional control

During the week prior to the start of the interventions, all participants will keep a written record of their morning weight (completely naked) and of all the food consumed with their respective weights. The total energy and intake of macronutrients will be evaluated through the individual registration of each participant using software adapted to the eating habits of the Spaniards (CESNID Nutritional Calculation Program V 1.0 - Center for Higher Nutrition I Dietetics assigned to the University of Barcelona). With these data the usual caloric intake will be established that:

a) It will be maintained during the 4 months of intervention in the exercise groups and

b) It will be reduced by an amount similar to the energy expenditure induced by the intervention with exercise in the participants of the caloric restriction and sedentary lifestyle group.

It is intended that the caloric expenditure of each exercise session be between 525-875 kcal or 2100-3500 kcal per week and generate a weight loss between 0.25-0.35 kg per week, for a total of 4-5.6 kg throughout the entire intervention. To adjust the weight loss in the caloric restriction

group, participants will reduce their energy intake by 300-500 kcal / day (2100-3500 kcal / week). In order to achieve the goals of diet and body weight, participants will be supervised weekly. All study participants will keep a record of their intake and body weight for two days a week (one week and one weekend) and submit the data to the research group to be analyzed and make the necessary adjustments.

Evaluation Procedure

Subjects must be evaluated for two consecutive days in the laboratory on eight different occasions:

a) before the start of diet training (day 1),

b) after the first week of training (+ 1 week)

c) after 1 month of intervention (+ 1 m)

d) after 2 months of intervention (+ 2 m)

e) after 3 months of the intervention (+ 3 m)

f) after 4 months of intervention (+ 4 m)

g) 1 month after the end of the intervention (to assess relapse)

h) 6 months after the end of the intervention (to assess relapse)

During the first day of the evaluation, the participants will carry out a test battery that will be administered in the morning, fasting and it is necessary that the 24 hours prior to the evaluation have not performed any type of physical training or ingested alcohol and / or coffee. During the second day and also on an empty stomach, the metabolic fitness of each subject will be evaluated.

DAY 1 of TEST

Dual-X-ray emission absorptiometry (DEXA)

The body composition of the body will be carried out by DEXA (Hologic Discovery-W, Hologic Corp., Waltham, MA, USA) in the manner described in other publications (Calbet, Moysi et al. 1998). Lean mass (g), fat mass (g) and bone mineral content (g) will be calculated from a full body test. The full body analysis will be complemented by a regional assessment of body composition in limbs and trunk. Through this analysis, fat and regional lean mass can be obtained with a coefficient of variation of less than 5%

Anthropometric measurements and resting blood pressure

Weight and height will be evaluated using a standard scale and a stadiometer that allows measuring with an accuracy of 0.1 cm and kg respectively (Seca, Vogel & Halke Hamburg, Germany). The body mass index (BMI; in kg / m2) will be calculated using the size and weight of the subject. The waist circumference (placing the midpoint between the costal flange and the iliac crests in the horizontal plane) will be measured using a 0.1 cm precision plastic measuring tape. For blood pressure measurement, participants will be accommodated supine for 15 min. Next, systolic (PAS) and diastolic (PAD) blood pressure will be determined with the help of an aneoroid sphygmomanometer (Heine Gamma 4. Germany). The average of the last 2-3 measurements will be used as the value of the PAS and the PAD.

Basal resting metabolism (RMR)

The consumption of pulmonary oxygen (VO_2) and carbon dioxide (VCO_2) will be measured during exercise and at rest (baseline resting metabolism) using an automatic online system (Oxycon Pro; Jaeger, Würzburg, Germany). Before each test a volume calibration and a gas calibration will be carried out using gases of known concentration. Basal resting metabolism (RMR) will be assessed by indirect calorimetry. Participants must be fasting (12h) and be resting supine in a comfortable bed with their head in a Plexiglas tent-type system for 35 min. The RMR will be carried out in a computerized way and using an open circuit with a ventilated shop type system using the data of the last 15 minutes to allow acclimatization. The room should be calm and with the light slightly off. Women who are not amenorrheic will always be evaluated at the same time of the menstrual cycle.

Intravenous glucose tolerance test

To evaluate the improvements in insulin sensitivity we will use a 60-minute intravenous glucose tolerance test (Tura, Sbrignadello et al. 2010). Participants will arrive at the laboratory after 12 hours of fasting and with 48 hours of exercise restriction ensuring they have taken a diet rich in hydrates the day before. After at least 10 minutes of supine rest, an intravenous catheter (Becton Dickinson S.A. Madrid, Spain) will be inserted into the antecubital vein. Next, a baseline sample will be extracted, followed by a 3 min manual infusion of a dose of 0.5g per kilogram of a 30% glucose solution (Grifols, Barcelona, Spain). In subjects with weights above 70 kg, 35 g of glucose will be infused at most according to the recommendations of the ICARUS Group (McCulloch, Bingley et al. 1993). After finishing the infusion, five milliliters of blood will be drawn at times 0, 2, 4, 6, 8, 10, 20, 30, 40, 50 and 60-min. For the calculation of the acute insulin response, the area under the curve of the first six samples will be taken into account. The insulin sensitivity index (CSI) will be calculated following the indications of Tura et al.

$$CSI = \alpha * [KG / (\Delta AUCINS / T)]$$

Where α is a scalar factor (0.276), KG is the rate of disappearance of glucose using a logarithmic slope of glucose, ΔAUCINS is the area under the curve of insulin concentration over baseline concentration and T is the time interval between samples of minute 10 and 50.

Blood chemistry analysis

After a supine position of 15 minutes and prior to the execution of the IVGTT, 10 ml of venous blood will be extracted for metabolite analysis. Plasma treated with Citrate and EDTA will be centrifuged at 3000 rpm for 10 min at 4 ° C. Plasma aliquots will be stored at -80 ° C for later analysis. Serum concentrations of Triglycerides, glucose, HDL-C, total cholesterol, glycated hemoglobin (HbA1c), TNF-alpha, IL-6, leptin, C-reactive protein and adiponectin will be measured using standard procedures. Serum levels of adiponectin, leptin, IL-6, IL-8, HbA1c, C-reactive protein and TNF-alpha will be measured by high-sensitivity ELISA specific to humans. For the measurement of triglycerides, total cholesterol, HDL-C, glucose, colorimetric analyzes will be used in a plate reader (Versamax, Molecular Devices, USA) using duly validated assays. Triglycerides: will be measured using a glycerol phosphate oxidase / peroxidase method (BioSystems, Spain) with a coefficient of variation (CV) of 1.7%. Total Cholesterol will be measured using a cholesterol oxidase / peroxidase reagent (BioSystems, Spain) with a CV of 1.0%. The HDL-C fraction: will be measured by the Phosphotungstate / Mg-Cholesterol oxidase / peroxidase method (BioSystems, Spain) with a CV of 3.2%. Glucose will be measured using glucose oxidase as a reagent (Thermo scientific, USA) with a CV of 3.0%. Insulin will be measured using a carbonyl metallo-immunoassay (CMIA; Architect i2000SR Abbott Lab). Free fatty acids will be measured with the reagent kit (HR series NEFA, WAKO, USA) with a CV of 4.1%. For immunoassays, the following high sensitivity tests specific to humans will be used: Adiponectin (Linco Research, USA) with a CV of 3.9%. Leptin (R&D Systems, USA) with a CV of 3.2%. IL-6 (Quantikine HS600; R&D Systems Europe) with a CV of 5.5%. TNF-alpha (Quantikine HSTA00C; R&D Systems Europe) with a CV of 3.5%. Reactive Protein C and HbA1c will be measured using properly validated high sensitivity kits (Gentaur Molecular Products, Belgium and Kalon Biological Limited, United Kingdom; respectively).

Day 2 of tests.

Economy of movement, maximum consumption of O2 and maximum fat oxidation

The peak in O2 consumption (VO2 peak) will be measured during a ramp exercise protocol, which will be individualized and executed in a cycle ergometer for which an indirect indirect calorimetry open system will be used (Oxycon Pro; Jaeger, Würzburg, Germany) . After a heating of 10-min at 50 watts, the load will be adjusted to another constant sub-maximum level for another 10 minutes. O2 uptake in ml · kg-1 · min-1 and Heart Rate (HR) in beats per minute (bpm) during submaximal loading will be used to calculate the economy of movement and cardiovascular adaptations to training. Participants will run 3-4 additional 3-minute loads to determine fat oxidation and intensity (% of VO2peak) at which the maximum amount of fat is oxidized (FatMax). Upon completion of this procedure, the load will increase 25 watts per minute until fatigue. The average of the three highest measurements will be used to establish the peak VO2. The FC will be measured during the test (Polar electroRS400, Finland), and the maximum FC reached will be recorded as (FCpico).

1 RM strength test

The maximum strength will be determined by 1-RM in the 6 exercises in which participants will train. 1-RM will be defined as the maximum load that the participant is able to lift by executing a full range of motion. Taking into account that this test is fatigued and due to the maximum effort increases the risk of injury, we will use an extrapolation method for the calculation of 1-RM using submaximal loads and measuring the mobilization speeds using a signal transducer (Sanchez-Medina, Perez et al. 2010). We believe that the evaluation of maximum strength is important, since a recent study by one of the researchers in this group suggests that muscle strength is more associated with an adequate cardio-metabolic condition than physical fitness per se (Solera Martínez, López Martínez et al. 2011).

Quality of life

Health-related quality of life (HRQL) will be measured using validated versions of the Spanish translation of the SF-36 health questionnaire (Martínez-González, López-Fontana et al. 2005). The questionnaire will be administered one week before the intervention and during the last week of it. With this tool, 8 dimensions of health are explored using scales of nine elements. Only the dimensions that explore physical health (physical functioning, role limitations, general health and health transitions) will be used in this study.

Table 2. Time-course of experimental testing.

**Before intervention**		**Exercise related adaptations**				**Detraining testing**	
24-48 h	1 week	1 month	2 months	3 months	4 months	1 month post-training	6 months post-training

On the eight occasions indicated, for two consecutive days, we will measure in all participants both the 5 components of the metabolic syndrome (central obesity measured by the waist circumference, high blood pressure figures, high fasting glucose, hyper-triglyceridemia, low HDL) such as physiological adaptations to training (fat-free mass, resting energy expenditure, VO2max, movement economy, maximum fat oxidation and strength gains). Additional measures will be added as described in Table 4 in order to expand the conclusions of the study.

Table 3: Main Variables.

Main criteria	Measurements
1) Central obesity	Waist circumference
2) Atherogenic dyslipidemia	Raised TG and reduced HDL
3) Glucose metabolism	Increased fasting glucose
4) Vascular disorder	High blood pressure

Table 4. Additional variables

Main criterio	Additional criterio	Measurement
1) Central obesity	Abnormal fat distribution	- Quantity and distribution of body fat (DEXA)<br><br>-BMI and anthropometric measurements (skinfolds)<br><br>- Adipokines (leptin and adiponectin)
2) Atherogenic dyslipidemia		- ApoB<br>- Free fatty acids
3) Glucose metabolism	Hiperglycemia/hyperinsulinemia	- Intravenous glucose tolerance test (IVGTT)<br>- HOMA-IR
4) Vascular disorder	Pro-inflammatory state<br>Exercise hemodynamics.	C reactiv protein<br>- Pro-inflammatory cytoquines (eg TNF-alpha, IL-6)<br>- Adiponectin<br>- O2 pulse (VO_2/FC)<br>- Peripheral resistances (PA/Q)
5) Metabolic flexibility		- Maximal fat oxidation<br>- Resting fat oxidation<br>- Anaerobic threshold

6) Fitness		- Cardiorespiratory fitness (VO_2peak)<br>- Faf free mass (DEXA)<br>- Maximal strength in upper and lower legs
7) Chronic stress-related hormonal changes (Bjorntorp 2001)		- Cortisol

Measurements performed on muscle biopsies

Muscle tissue specimens (i.e., 20-50 mg) from the vastus lateral femoral quadriceps will be obtained, using the needle biopsy technique (Bergstrom 1962). Taking into account that this is the most invasive procedure of the study and that it can limit the interest of the participants, we will only try to collect samples in 8 participants of each group (32 in total). Samples will be obtained on the day before the start of the intervention and the day after the end of the intervention (4 months apart). In the extracted tissue, the concentration of intramuscular triglycerides will be measured after fat extraction with a 2: 1 solution of chloroform and methanol and 4% KOH in a fluorometer (Frayn and Maycock 1980). The composition of intramuscular triglycerides will be analyzed by identifying the fatty acid profile: (C17: 0 (heptadecanoic-CoA), C16: 0 (palmityl-CoA), C16: 1 (palmitoleoyl-CoA), C18: 0 (steariol-CoA), C18: 1 (oleoiol-CoA), and C18: 2 (linoleiol-CoA)). Additionally, we will measure glycogen concentrations after obtaining tissue homogenization and achieving glycogen hydrolysis using a conventional assay (Passonneau and Lauderdale 1974).

The research group is also interested in measuring the effects of exercise in signaling the effects of insulin in patients with metabolic syndrome. We hypothesize that tissue accumulation of intermediaries of incomplete fat metabolism (ie, diacylglycerol and acyl co-enzyme A) activates pro-inflammatory factors (JNK, PKC, IkK / NFbk) that alter signals interacting with the receptor (IRS-1 , Akt) (Schenk and Horowitz 2007). Some of the elements mentioned above will be measured using the "Western blott" technique.

In summary, whole cell lysates will be prepared as previously described (Schenk, Harber et al. 2009). Samples (25 µg) will be separated using 8% SDS-PAGE and transferred to an ice bath at 200 mA. After blocking the membranes, tests for phosphorylated JNK (p-JNK; no. 9251, Cell Signaling, USA), JNK-1 (sc-474; Santa Cruz Biotechnology, USA) and IκB-β will be performed (nos 9242 and 9248; Cell Signaling USA). The membranes will be subsequently incubated in the appropriate antibody for 60 minutes and analyzed using enhanced chemo-luminescence (Amersham Biosciences, Piscataway, USA). Bands will be quantified by densitometry (Fluor Chem SP, Alpha Innotech, USA). The densities of the bands for each subject will be analyzed and expressed in relation to a standard (human muscle) that will be run in duplicate in all gels.

Statistical analysis

The results will be presented as means ± SD. The change in the mean of each group will be presented as an estimated error of the mean (SEM) and will be supported by 95% confidence intervals (95% CI). Differences between groups will be considered significant when the 95% CI does not include the value of zero. (Gardner and Altman 1986). To assess differences between groups, a covariance analysis (ANCOVA) will be used, where the time and treatment assigned will be the factors and initial values of the analyzed variables, covariates (Vickers and Altman 2001). P values <0.05 will be considered significant. All statistical analysis will be performed using the SPSS software (version 19.0).

References

Aizawa, K., J. K. Shoemaker, et al. (2009). "Metabolic syndrome, endothelial function and lifestyle modification." Diab Vasc Dis Res. 6(3): 181-9.

Alberti, K. G., P. Zimmet, et al. (2006). "Metabolic syndrome-a new world-wide definition. A consensus statement from the International Diabetes Federation." Diabet Med 23: 469-480.

Ara, I., S. Larsen, et al. (2011). "Normal mitochondrial function and increased fat oxidation capacity in leg and arm muscles in obese humans." International Journal of Obesity , 35: 99-108.

Ballor, D. L., V. L. Katch, et al. (1988). "Resistance weight training during caloric restriction enhances lean body weight maintenance." Am J Clin Nutr 47(1): 19-25.

Bergstrom, J. (1962). " Muscle electrolytes in man." Scand J. Clin. Lab Invest. 14: 1-110.

Bjorntorp, P. (2001). "Heart and soul: stress and the metabolic syndrome." Scand Cardiovasc J 35: 172-7.

Blair, S. N. and S. Brodney (1999). "Effects of physical inactivity and obesity on morbidity and mortality: current evidence and research issues." Med Sci Sports Exerc. 31(11): S646-62.

Bo, S., L. Gentile, et al. (2005). "The metabolic syndrome and high C-reactive protein: prevalence and differences by sex in a southern-European population-based cohort." Diabetes Metab Res Rev 21(6): 515-24.

Bouchard, C., F. T. Dionne, et al. (1992). "Genetics of aerobic and anaerobic performances." Exerc Sport Sci Rev 20: 27-58.

Boule, N. G., E. Haddad, et al. (2001). "Effects of exercise on glycemic control and body mass in type 2 diabetes mellitus: a meta-analysis of controlled clinical trials." JAMA 286: 1218-1227.

Calbet, J. A., J. S. Moysi, et al. (1998). "Bone mineral content and density in professional tennis players." Calcif Tissue Int 62: 491-496.

Caspersen, C. J. (1987). "Physical inactivity and coronary heart disease." Physician and Sportsmedicine 15(11): 43-44.

Deen, D. (2004). "Metabolic Syndrome: Time for Action." Am Fam Physician 69(2875-82): 2887-8.

Del Coso, J., N. Hamouti, et al. (2010). "Aerobic fitness determines whole-body fat oxidation rate during exercise in the heat." Appl Physiol Nutr Metab. 35(6): 741-8.

Dunstan, D. W., I. B. Puddey, et al. (1998). "Effects of a short-term circuit weight training program on glycaemic control in NIDDM." Diabetes Res Clin Pract 40: 53-61.

Durstine, J. L., P. W. Grandjean, et al. (2002). "Lipids, lipoproteins, exercise." J Cardiopulm Rehabil 22: 385-398.

Durstine, J. L. and W. L. Haskell (1994). "Effects of exercise training on plasma lipids and lipoproteins." Exer Sport Sci Rev 22: 447.

Frayn, K. N. and P. F. Maycock (1980). "Skeletal muscle triaglycerol in the rat: methods for sampling and measurement, and studies of biological variability." J Lipid Res 21(1): 139-144.

Gami, A. S., B. J. Witt, et al. (2007). "Metabolic syndrome and risk of incident cardiovascular events and death: a systematic review and meta-analysis of longitudinal studies." J Am Coll Cardiol 49: 403-414.

Gardner, M. J. and D. G. Altman (1986). "Confidence intervals rather than P values: estimation rather than hypothesis testing." Br Med J 292: 746-750.

Helge, J. W., R. Damsgaard, et al. (2008). "Low-intensity training dissociates metabolic from aerobic fitness." Scand J Med Sci Sports 18(1): 86-94.

Hildrum, B., A. Mykletun, et al. (2007). "Age-specific prevalence of the metabolic syndrome defined by the International Diabetes Federation and the National Cholesterol Education Program: the Norwegian HUNT 2 study." BMC Public Health 7: 220.

Hwang, L. C., C. H. Bai, et al. (2007). "Gender difference on the development of metabolic syndrome: a population-based study in Taiwan." Eur J Epidemiol 22(12): 899-906.

INE. (2006). "Encuesta Nacional de Salud; Instituto Nacional de Estadística." from http://www.ine.es/jaxi/tabla.do.

Kashyap, S. R. and R. A. De Fronzo (2007). "The insulin resistance syndrome: physiological considerations." Diab Vasc Dis Res 4: 13-19.

Katzmarzyk, P. T., A. S. Leon, et al. (2003). "Targeting the metabolic syndrome with exercise: evidence from the HERITAGE Family Study." Med Sci Sports Exerc 35: 1703-1709.

Kiernan, M., A. C. King, et al. (2001). "Men gain additional psychological benefits by adding exercise to a weightloss program." Obes Res 9: 770-777.

Larsen, S., I. Ara, et al. (2009). "Are substrate use during exercise and mitocondrial respiratory capacity decreased in arm and leg muscle in type 2 diabetes?" Diabetologia 52(7): 1400-8.

Lillioja, S., C. Bogardus, et al. (1985). "Relationship between insulin-mediated glucose disposal and lipid metabolism in man." J Clin Invest 75(4): 1106-15.

Lopez-Candales, A. (2001). "Metabolic syndrome X: a comprehensive review of the pathophysiology and recommended therapy." J Med 32: 283-300.

Lucía, A., J. Hoyos, et al. (2000). "Heart rate and performance parameters in elite cyclists: a longitudinal study." Med Sci Sports Exerc. 32(10): 1777-82.

Martínez-González, M. A., C. López-Fontana, et al. (2005). "Validation of the Spanish version of the physical activity questionnaire used in the Nurses' Health Study and the Health Professionals' Follow-up Study." Public Health Nutr 8: 920-7.

Martínez-Vizcaíno, V., M. S. Martínez, et al. (2010). "Validity of a single-factor model underlying the metabolic syndrome in children: a confirmatory factor analysis." Diabetes Care 33(6): 1370-2.

McArdle, W. D., F. I. Katch, et al. (1996). Physical activity, health and aging. Exercise Physiology; energy, nutrition and human performance. D. Balado. Baltimore, Williams and Wilkins: 635-655.

McCulloch, D. K., P. Bingley, et al. (1993). "Comparison of bolus and infusion protocols for determining acute insulin response to intravenous glucose in normal humans." Diabetes care. 16: 911-15.

Mikines, K. J., B. Sonne, et al. (1988). "Effect of physical exercise on sensitivity and responsiveness to insulin in humans." Am J Physiol. 254: E248-59.

Milesis, C. A., M. L. Pollock, et al. (1976). "Effects of different durations of physical training on cardiorespiratory function, body composition, and serum lipids." Res Q. 47(4): 716-25.

Mora-Rodriguez, R., J. Coso, et al. (2010). Changes in Markers for Cardiovascular and Metabolic Disease Risk Evident after Only 1-2 weeks of a High Saturated Fat Diet in Overweight adults. Diabetes.

Passonneau, J. V. and V. R. Lauderdale (1974). "A comparison of three methods of glycogen measurement in tissues." Anal. Biochem. 60: 405-412.

Position stand (1993). "Physical activity, physical fitness, and hypertension." Med Sci Sports Exerc. 25(10): i-x.

Reaven, G. (2006). "The metabolic syndrome: is this diagnosis necessary?" Am J Clin Nutr 83: 1237-1247.

Reaven, G. M. (1988). "Role of insulin resistance in human disease." Diabetes 1595-1607(37).

Reaven, G. M. (1995). "Characteristics of metabolic syndrome." Endocrinol Metab 2(Suppl B): 37-42.

Remsberg, K. E., N. L. Rogers, et al. (2007). "Sex differences in young adulthood metabolic syndrome and physical activity: the Fels longitudinal study." Am J Hum Biol 19(4): 544-50.

Ren, J. M., C. F. Semenkovic, et al. (1994). "Exercise induces rapid increases in GLUT4 expression, glucose transport capacity, and insulin-stimulated glycogen storage in muscle " J Biol Chem 269: 14396-14401.

Sady, S. P., E. M. Cullinane, et al. (1988). "Elevated high-density lipoprotein cholesterol in endurance athletes is related to enhanced plasma triglyceride clearance." Metabolism 37(6): 568-72.

Sánchez-Chaparro, M., E. Calvo-Bonacho, et al. (2008). "Occupation-related differences in the prevalence of metabolic syndrome." Diabetes Care 31(9): 1884-5.

Sanchez-Medina, L., C. E. Perez, et al. (2010). "Importance of the propulsive phase in strength assessment. ." Int J Sports Med 31: 123-129.

Schenk, S., M. P. Harber, et al. (2009). "Improved insulin sensitivity after weight loss and exercise training is mediated by a reduction in plasma fatty acid mobilization, not enhanced oxidative capacity." J Physiol 587(20): 4949-4961.

Schenk, S. and J. F. Horowitz (2007). "Acute exercise increases triglyceride synthesis in skeletal muscle and prevents fatty acid-induced insulin resistance." J Clin Invest 117(6): 1690-8.

Solera Martínez, M., S. López Martínez, et al. (2011). "Validez de un modelo de único factor en el síndrom metabólico en adultos." Rev Española de Cardiologia.

Stensvold, D., A. E. Tjønna, et al. (2010). "Strength training versus aerobic interval training to modify risk factors of metabolic syndrome." J Appl Physiol 108: 804-810.

Tremblay, A., E. Doucet, et al. (1999). "Metabolic fitness in active reduced-obese individuals." Obes Res. 7(6): 556-63.

Tremblay, A., E. Fontaine, et al. (1986). "The effect of exercise-training on resting metabolic rate in lean and moderately obese individuals." Int J Obes 10(6): 511-7.

Tura, A., S. Sbrignadello, et al. (2010). "An empirical index of insulin sensitivity from short IVGTT: validation against the minimal model and glucose clamp indices in patients with different clinical characteristics." Diabetologia 53: 144-152.

Venables, M. C. and A. E. Jeukendrup (2008). "Endurance training and obesity: effect on substrate metabolism and insulin sensitivity." Med Sci Sports Exerc 40: 495-502.

Vickers, A. J. and D. G. Altman (2001). "Statistics notes: analysing controlled trials with baseline and follow up measurements." Br Med J 323: 1123-1124.

Vincent, W. J. (1999). Statistics in kinesiology. Champaign, IL, Human Kinetics.

Wilson, P. W., R. B. D'Agostino, et al. (2005). "Metabolic syndrome as a precursor of cardiovascular disease and type 2 diabetes mellitus." Circulation 112(20): 3066-72.

Wu, T., X. Gao, et al. (2009). "Long-term effectiveness of diet-plus-exercise interventions vs. diet-only interventions for weight loss: a meta-analysis." Obes Rev. 10(3): 313-23.

Zuti, W. B. and L. A. Holding (1976). "Comparing diet and exercise as weight reduction tools." Physician and Sportsmedicine 4: 49-53.

INFORMATION SHEET AND INFORMED CONSENT OF THE PARTICIPANT

Research team:

- Dr. Ricardo Mora Rodríguez (Project Manager). University Professor, School of Nursing and Physiotherapy; Faculty of Sports Sciences. University of Castilla-La Mancha (UCLM)
- Juan Fernando Ortega Fonseca. Graduate in medicine and surgery. Specialty in Sports Medicine. El Bosque University (Colombia).
- Ignacio Ara Royo. University holder. Faculty of Sports Sciences. University of Castilla-La Mancha (UCLM).
- Vicente Martínez Vizcaino. University School Professor. Nursing school. University of Castilla-La Mancha (UCLM).
- Jorn Helge. Senior Professor-Researcher, Dept. Biomedical Sciences. University of Copenhagen

Objective. Overweight leads over the years to abdominal obesity and is suffering from metabolic disorders (hypercholesterolemia, diabetes) that increase the risk of cardiovascular disease. The objective of this study is to counteract this clinical picture (metabolic syndrome) through the realization of a 4-month exercise program that we will administer to you completely free of charge. We want to know the consequences of physical exercise on the factors associated with overweight and obesity (high blood lipids, hypertension and pre-diabetes) and compare it with a diet where calories are reduced.

Requirements to participate. You and another 119 adults between 25 and 65 will participate in this study. You will all be overweight and not participate in sports activities. Half of the participants in addition to being overweight will have at least 3 of the following 5 factors; i) high blood pressure, ii) fasting high blood glucose, iii) high blood triglycerides, iv) low blood HDL cholesterol, v) abdominal obesity. If you have 3 of these 5 factors, you will be in the group called "metabolic syndrome". Participants who are overweight will only be the "control group". This group should not have any pathology or be taking medication for the duration of the study (except oral contraceptives). If you are a woman, you should not be pregnant.

Participants in the "metabolic syndrome" group should not have any other endocrine pathology other than carbohydrate intolerance or hypelipidemia. Your hormonal levels (except insulin) should be in normal clinical ranges. People who are being treated for hypertension or who receive hypoglycemic or hypolipidemic medications may also participate. In these participants, the opinion of their family doctor will be requested before enrolling them in the study. During the study your doctor can adjust the treatment according to the particular needs of each individual and their degree of evolution during the study. The research team will ask the doctor for information about the change in the dose of his medication in order to interpret the results.

Other exclusion criteria will be: recent surgery, cardiovascular disease (especially coronary heart disease, heart valve disease, heart failure, complex ventricular arrhythmias), kidney, liver, respiratory or neuromuscular diseases.

Groups in the study. Within each group ("control" and "metabolic syndrome") you will be randomly assigned one of the following 2 treatments: Physical training for 4 months or, reduction of calories in the diet for 4 months. Therefore, you will belong to one of the 4 experimental groups that are summarized in the table below:

CONTROL GROUP		METSYN GROU	
EXERCISE TRAINING	DIET	EXERCISE TRAINING	DIET

Medical examination and exercise test before the study. Before beginning the study, a medical history with examination will be performed, and you will be asked to undergo a stress test by pedaling in an ergometer cycle. At rest and during exercise we measure your heart's response using an electrocardiograph and blood pressure to rule out cardiac or cardiovascular system abnormalities. In addition, during the test part of the exhaling air will be collected to calculate the oxygen consumption and its maximum aerobic capacity. All these procedures will be performed by the specialist in sports medicine of the research team. You may feel fatigued at the end of the test. On rare occasions it can be experienced after exertion, dizziness, gastrointestinal disorders and even (in people with an undiagnosed cardiac pathology) sudden death. If you experience dizziness, shortness of breath, or sudden pain during this test or in the others in this study, you should notify us immediately.

Diet Groups: If you consent to participate in this study, and you have to be part of the diet group, you will be subjected to a diet where you will reduce the amount of calories you eat by about 300-500 per day. This reduction is not drastic, but the difficulty entails your commitment to maintain it for 4 months. After an analysis of your usual diet by an expert in dietetics and nutrition you will be instructed to modify your diet without loss of the necessary nutrients. You will be asked to keep a record of the food you eat and your body weight as soon as you get up. The team of researchers will supply scales for weighing food and your body weight as well as a computer program for caloric counting. Every 4 days will bring us the data collected (calories ingested and body weights) to be analyzed and make the necessary adjustments. On these days, body fat (bioimpedance) and blood pressure will be measured.

Physical Training Group: The exercise will be carried out under our supervision on an exercise bike, treadmill or similar machines. The training program will consist of 3 weekly sessions of

interval aerobic exercise and 1 weekly session of strength training. The aerobic exercise will consist of a continuous work of 55 min of duration by training. Strength training sessions will consist of weekly strengthening sessions focused on upper limbs, trunk and lower limbs. The workload will increase as training adaptations occur. In each exercise session, your heart rate and your subjective sensation of effort will be measured. Every 2 weeks your blood pressure will be measured and by analyzing your breathing we will calculate how much fat you oxidize during exercise in the aerobic workout.

Measurements during the experiment

You will be asked to go to the laboratory in the morning (7-9 am), without having breakfast or training the day before on the following occasions:

a) before the start of diet training (day 1)

b) after the first week of training (+ 1 week)

c) after 1 month of intervention (+ 1 m)

d) after 2 months of intervention (+ 2 m)

e) after 3 months of the intervention (+ 3 m)

f) after 4 months of intervention (+ 4 m)

g) 1 month after the end of the intervention (to assess relapse)

h) 6 months after the end of the intervention (to assess relapse)

Estimation of body composition. He will weigh himself naked and will be carved. Next, the thickness of the folds of certain parts of your skin (back of the arm, thigh, etc.) will be measured to estimate the percentage of body fat. You will also calculate the amount of body fat with an apparatus that circulates through your body a weak electrical current that you will not feel (electrical bioimpedance).

Cardiovascular variables and resting metabolism. After these measurements, you will lie on a stretcher, an elastic band will be placed around your chest to measure your heart rate and a mask around your mouth and nose to collect exhaled air. This mask allows you to breathe normally. This air will be analyzed for 15 minutes to calculate your basal metabolism (calories spent at rest).

Intravenous glucose tolerance test. After these resting measurements, a flexible line will be placed in a vein in the forearm through which a blood sample (about 5 milliliters) will be taken. You will then be injected in about 4 minutes 35 grams of sterilized dissolved glucose. Thereafter, a blood sample will be collected at 1, 2, 4, 6, 8, 10, 12, 14, 20, 30, 40 and 60 minutes. In these samples we will analyze the concentration of glucose and insulin in your blood.

Lipidic profile. In the blood sample we take before injecting glucose, the concentration of triglycerides, total cholesterol and its fractions and the type of fatty acids in your blood (saturated

vs. mono-polyunsaturated) will be measured. Also, we will analyze in your blood liver damage parameters such as transaminases and C-reactive protein.

Muscle biopsy. Following, a small sample of the thigh muscle will be removed. After cleaning and disinfecting the skin, a local-acting anesthetic similar to that used by dentists will be applied and a small portion of muscle tissue (approximately the size of 2-3 grains of rice) will be removed through an incision in the thigh skin After the extraction, the wound will be closed with some approach strips (without stitches) and a compressive bandage that should be worn for at least 48 hours.

Risks and inconveniences. There is a risk of developing a small bruise (effusion) in the place where the path was in the arm. This risk is reduced if pressure is applied for 10 minutes after removing the track. In both the puncture of the arm and those of the skin of the thigh there is a risk of infection, which is minimized with the sterile procedures that we follow. Some people get dizzy during punctures in the skin of the arm or thigh but the risks of dizziness accident are reduced by performing such a puncture with you lying on the couch. Finally, some people comment on a certain loss of sensation in the area near the muscle biopsy (2-3 cm), but this loss of sensation in the skin is temporary and remits in 2-3 weeks.

INFORMED CONSENT SHEET

NAME AND SURNAME ____

Birthdate _____________ ID number ____________________________

Address ____

Phone number ______________________________

MAIN RESEARCHER *RICARDO MORA RODRÍGUEZ*

Exercise physiology laboratory. University of Castilla La Mancha. Toledo

1. I have read the project information sheet and have had the possibility to discuss the details with the principal investigator and ask him any questions. The project manager has explained to me the purpose of the tests that are going to be done and I have fully understood everything that has been explained to me.

2. I agree to take part in this study and understand that I am completely free to abandon it at any time I wish or refuse to perform any of the measurement procedures.

3. I understand that the tests performed are part of a research project that will not give me any personal gain but that participation is voluntary. The results of this study aim to promote knowledge in Biomedical Sciences and I understand that the procedures described have been approved by an ethical clinical research committee.

I fully and freely consent to participate in the project entitled: Title of the study. "Effects of 16 weeks of combined aerobic-strength training on the individual components of the metabolic syndrome; temporal evolution of the improvements "which has been explained to me in detail.

Volunteers signature ____________________________________

Date____

I confirm that I have explained to the volunteer (named above) the purpose and risks of the tests to be performed.

Researcher signature ____________________________________

Date ____
