# Supplementary material for: Women with metabolic syndrome show similar health benefits from high-intensity interval training than men
Source: PLoS One. 2019 Dec 10;14(12):e0225893. doi: 10.1371/journal.pone.0225893 (PMC6903716; doi:10.1371/journal.pone.0225893)
Supplement: S3 File — (PDF) [file pone.0225893.s003.pdf]

INFORME PARA EL COMITÉ ÉTICO DE INVESTIGACIÓN CLÍNICA DEL COMPLEJO HOSPITALARIO UNIVERSITARIO DE ALBACETE

Título del estudio. **"Efectos de 16 semanas de entrenamiento combinado aerobico-fuerza en los componentes individuales del Síndrome Metabólico; evolución temporal de las mejoras"**

Equipo de investigadores:

- **Dr. Ricardo Mora Rodríguez (Responsable del proyecto)**. Catedrático de Universidad, Escuela de Enfermería y Fisioterapia; Facultad de Ciencias del Deporte. Universidad de Castilla-La Mancha (UCLM)
- **Juan Fernando Ortega Fonseca.** Licenciado en Medicina y Cirugía. Especialidad en Medicina Deportiva. Universidad El Bosque (Colombia).
- **Ignacio Ara Royo.** Titular de Universidad. Facultad de Ciencias del Deporte. Universidad de Castilla-La Mancha (UCLM).
- **Vicente Martínez Vizcaino**. Catedrático de Escuela Universitaria. Escuela de Enfermería. Universidad de Castilla-La Mancha (UCLM).
- **Jorn Helge**. Profesor-Investigador senior, Dept. Biomedical Sciences. University of Copenhagen.

Precedentes del estudio

Síndrome metabolico

El término "síndrome metabólico" identifica múltiples factores de riesgo para el desarrollo de la enfermedad cardiovascular e incluye la coexistencia de adiposidad central, dislipemia, hipertension arterial y trastornos en el metabolismo de los carbohidratos (Reaven 1988). El síndrome metabólico es llamado también síndrome de resistencia a la insulina, ya que la resistencia a la insulina y la hiperinsulinemia compensatoria son considerados como sus componentes principales (Reaven 1995). El perímetro de cintura se ha establecido como el criterio de inclusión principal de acuerdo a la definición establecida por la Federación Internacional de Diabetes (Alberti et al. 2006). La prevalencia de los componentes del síndrome metabólico en las sociedades occidentales es superior al 20% en la población adulta (Hildrum et al. 2007). En referencia a la población española, se ha estimado que el síndrome metabólico es padecido por el 10% de la población en edad laboral. (Sánchez-Chaparro et al. 2008). La prevalencia del síndrome metabólico aumenta con la edad y con el incremento de peso. Dado que la población española está envejeciendo (INE 2006), y que existe una tendencia entre la gente de entre 30-50 años a ganar grasa corporal, se espera que el síndrome metabólico incremente su prevalencia en nuestro país en un futuro próximo.

El síndrome metabólico aumenta el riesgo de sufrir enfermedad cardiovascular (particularmente enfermedad coronaria), diabetes tipo 2 y la mortalidad relacionada don dichos padecimientos (Gami et al. 2007; Wilson et al. 2005). En los Estados Unidos se ha estimado que pronto el síndrome metabólico sobrepasará al consumo de tabaco como el principal factor de riesgo para el desarrollo de enfermedad cardiovascular (Deen 2004). Las adaptaciones

fisiológicas que se derivan del entrenamiento físico tienen el potencial de disminuir la diseminación de este síndrome entre la población. Por lo tanto, consideramos que desde el punto de vista social es de gran importancia la cronología en la que aparecen los efectos derivados del ejercicio. Esta información podría aumentar la efectividad de la prescripción de programas de ejercicio en el ámbito clínico (entrenamiento supervisado) y extra-clínico (centros de acondicionamiento físico).

Etiología del síndrome metabólico y efectos del entrenamiento

En 1992 la American Heart Association (Asociación Americana del Corazón) incluyó a la inactividad física como uno de los principales factores para el desarrollo de enfermedad coronaria, con una importancia similar a la de otros factores de riesgo como la hipercolesterolemia, la hipertensión arterial y el tabaquismo. A diferencia de los otros factores de riesgo, la inactividad física es especialmente relevante, teniendo en cuenta que existe un importante porcentaje de población que no realiza actividad física en comparación con la que consume tabaco (27%) o tiene hipercolesterolemia (29%). Entre la población española mayor de 16 años, el 80% trabaja mientras están sentados o de pie (inactividad física) y el 61% declara no participar en actividad física durante su tiempo libre (INE 2006). De otro lado, la pato-fisiología del síndrome metabólico sugiere que el ejercicio físico regular puede ser un potente estimulo para reducir la prevalencia del síndrome metabólico. Algunos de las más relevantes adaptaciones producidas por el entrenamiento se oponen a los componentes del síndrome metabólico.

El entrenamiento físico de adecuada intensidad y duración produce adaptaciones metabólicas que provocan el incremento de la oxidación lipídica y que resultan en una reducción de la grasa corporal, mejoría en el perfil lipídico, el metabolismo de la glucosa y la salud cardiovascular. Que sepamos, actualmente no se conoce completamente si el síndrome metabólico produce algún trastorno en el sistema molecular responsable de las adaptaciones derivadas del entrenamiento. La hipótesis que proponemos en cuanto al proceso temporal en el que ocurren los acontecimientos que genera el síndrome metabólico se expone a continuación. La obesidad abdominal provoca un incremento de los lípidos circulantes en sangre que mediante mecanismos y procesos aún desconocidos genera la resistencia a la insulina (Lillioja et al. 1985). A continuación, la resistencia a la insulina genera hiperglicemia, la cual en combinación con un perfil lipídico desfavorable (niveles aumentados de triglicéridos y lipoproteínas de baja densidad-LDL) conduce a la enfermedad cardiovascular. La enfermedad cardiovascular tiene probablemente su origen a nivel endotelial como consecuencia de una disfunción celular (i.e. estrés oxidativo) que resulta en un daño vascular y en la formación de la placa de ateroma (Lopez-Candales 2001).

Efectos del ejercicio sobre la resistencia a la insulina

Subyacente al síndrome metabólico se encuentra la resistencia a la insulina, que habitualmente se encuentra ligado al incremento en la adiposidad abdominal (Reaven 2006). Las bases moleculares de los mecanismos que producen la resistencia a la insulina no están del todo comprendidas. Las anormalidades en los receptores y/o procesos post-receptores han sido previamente identificadas y analizadas, así como las diferentes respuestas entre los diversos tejidos corporales dependiendo de su pérdida de sensibilidad a la acción de la insulina (Kashyap and De Fronzo 2007). Aunque casi el 50% de la etiología de la resistencia a la insulina se puede atribuir a la herencia, el restante 50% está relacionado con el nivel de adiposidad y los acidos grasos circulantes en la sangre (Lillioja et al. 1985). Se ha identificado una fuerte relación entre el

nivel de entrenamiento y la sensibilidad a la insulina. El ejercicio de forma aguda incrementa la sensibilidad a la insulina para inducir la captación de glucosa por parte del músculo. Las mejoras producidas por el ejercicio físico de forma aguda parece que se relacionan con cambios en la señalización de la insulina en respuesta a la contracción muscular, como puede ser el incremento de la traslocación de los transportadores de glucosa (GLUT4) a la superficie de la célula (Ren et al. 1994).

Sin embargo, los efectos de una sola sesión de ejercicio (60 minutos de pedaleo continuo) en la acción de la insulina son de corta duración (hasta las 48h (Mikines et al. 1988b)) lo cual explica la recomendación de ejercitarse de forma frecuente (i.e. cada dos días). A largo plazo, el entrenamiento modifica la utilización de sustratos energéticos incrementando la oxidación lipídica que puede desembocar en la pérdida de masa grasa. La reducción de la grasa corporal es claramente un mecanismo a través del cual el ejercicio mejora la sensibilidad a la insulina a lo largo del tiempo. El entrenamiento también aumenta el contenido de GLUT4, la actividad de la glucógeno sintetasa, la actividad enzimática mitocondrial y su densidad en el musculo, mejora la función endotelial y altera el tipo de fibras musculares (Venables and Jeukendrup 2008).

Efectos del ejercicio sobre la hipertensión

La obesidad es un factor de riesgo independiente para el desarrollo de hipertensión arterial. El entrenamiento aeróbico puede reducir las cifras de presión arterial sistólica y diastólica (i.e. 14%) especialmente en pacientes con hipertensión moderada o límite. Los mecanismos propuestos como responsables de este efecto incluyen una reducida actividad nerviosa simpática, una menor resistencia periférica al paso del flujo sanguíneo, y modificando la función renal, en particular aumentando la excreción de sodio (McArdle et al. 1996). No obstante, las reducciones en la presión arterial en relación al entrenamiento no siempre se producen en el largo plazo. Aún así, incluso si el ejercicio no es efectivo para la normalización de la presión arterial, genera beneficios para la salud que superan la falta de un efecto hipotensor. Como prueba de ello, el incremento de la mortalidad asociada a la hipertensión se elimina cuando la aptitud física está incrementada (McArdle et al. 1996).

Recientes estudios han informado que el entrenamiento entre el 40-70% del VO_{2max} tiene el mismo o incluso mejor efecto que el entrenamiento de alta intensidad, en lo referente a la reducción en las cifras de presión arterial. Parece que el entrenamiento físico moderado puede provocar las mismas reducciones en la presión arterial que el ejercicio de alta intensidad pero sin los posibles riesgos asociados con la práctica de ejercicio de alta intensidad (i.e. fatiga, infarto agudo del miocardio, lesiones musculo-esqueléticas). Con respecto al curso clínico de la relación entre ejercicio y presión arterial, la mayoría de los estudios han mostrado que las cifras de presión arterial se reducen de forma temprana (entre las 3 semanas y los 3 meses) de haber comenzado el entrenamiento. También se ha encontrado que la reducción de la presión arterial diastólica se relaciona con la duración del entrenamiento mientras que la reducción de la presión diastólica no. Por tanto, parece que la presión arterial puede ser reducida de forma temprana al inicio del entrenamiento aeróbico; prolongando dicho entrenamiento más de 3 meses podría resultar en mayores descensos. (Position stand 1993).

Efectos del ejercicio en la hiperlipidemia

El colesterol y los triglicéridos son los dos tipos más comunes de lípidos asociados con la enfermedad coronaria y para ser transportadas se combinan con unas proteínas formando las lipoproteínas. Se cree que durante el transporte de las lipoproteínas de baja densidad (LDL;

compuestas 45% de colesterol y 10% triglicéridos) y de las lipoproteínas de muy baja densidad (VLDL; 20% colesterol y 70% triglicéridos) se libera colesterol que participa en el proceso de formación de las placas de ateroma y la consiguiente ateroesclerosis. Mientras que las lipoproteínas de alta densidad (HDL; 18% colesterol y 2% triglicéridos) promueven la captación de colesterol desde los tejidos periféricos (incluyendo las paredes arteriales) hacia el hígado para la síntesis de bilis. Existen evidencias que muestran una asociación causal entre la reducción de las HDL y el incremento en el riesgo de padecer enfermedad coronaria (Caspersen 1987). El ejercicio físico periódico tiene un leve efecto en los niveles de LDL en comparación con el generado por otras intervenciones que resultan en cambios en la grasa corporal, como la reducción en la grasa total de la dieta o del colesterol.

Sin embargo, los niveles de HDL incrementan en mujeres y hombres sedentarios de todas las edades cuando inician ejercicio aeróbico de moderada y/o vigorosa intensidad (Durstine and Haskell 1994). Los cambios favorables en el perfil lipídico asociados con el ejercicio aeróbico se producen independientemente de los cambios en la masa corporal. Esta es una de las bases para las recientes recomendaciones de los programas de ejercicio para personas con sobrepeso, beneficiosos aunque no generen pérdida de peso (i.e. en forma pero con exceso de peso – “fit but fat” (Blair and Brodney 1999)). Parece que estos cambios favorables en el perfil lipídico con el ejercicio físico regular se relacionan con la mayor capacidad para reducir el nivel de los triglicéridos en la sangre (Sady et al. 1988). Los valores absolutos de HDL parece que están en parte genéticamente determinados. Por último, los programas que incluyen entrenamiento de fuerza parece que tienen poco o ningún efecto sobre las lipoproteínas en sangre. Debemos mantener siempre presente que el ejercicio aumenta el volumen plasmático lo cual produce una reducción de la concentración de HDL y otras lipoproteínas, por tal motivo el cociente de colesterol total / HDL-c parece un índice más preciso para evaluar los efectos del entrenamiento en el perfil lipídico.

Efectos del ejercicio o la dieta en la grasa abdominal.

Normalmente, las personas obesas pierden peso y grasa más rápidamente que las personas con peso normal. Un programa de entrenamiento aeróbico de suficiente duración e intensidad reduce la grasa corporal y mantiene la masa libre de grasa en personas con obesidad abdominal (Zuti and Holding 1976). Adicionalmente, los programas que incluyen ejercicio con menor demanda energética como el entrenamiento de fuerza ayudan al mantenimiento o incluso el aumento del tejido libre de grasa (i.e. músculo), aún ante la presencia de pérdida de peso (Ballor et al. 1988). Dado que la masa libre de grasa es metabólicamente más activa que la grasa, su conservación contribuye al mantenimiento de un mayor metabolismo energético basal, y una mayor oxidación lipídica durante el reposo (Tremblay et al. 1986). La duración del ejercicio más que la intensidad determina la pérdida de peso. La pérdida de grasa corporal se maximiza cuando se realiza ejercicio físico durante 45 minutos en comparación con sesiones de 15 y 30 minutos caminando y corriendo (Milesis et al. 1976).

La dieta hipocalórica y el ejercicio son las dos estrategias más usadas para reducir el peso. De qué forma la “dieta mas ejercicio” es más efectiva para perder peso que “solo dieta” a largo plazo, no está del todo establecido (Wu et al. 2009). Un meta-análisis que incluía solo estudios clínicos aleatorizados concluyó que la adición de ejercicio físico a otras intervenciones diseñadas para reducir peso, mejoraba diversas variables relacionadas con la salud como el perfil lipídico, la presión arterial, la sensibilidad a la insulina y el bienestar psíquico, independientemente del efecto sobre la grasa corporal (Kiernan et al. 2001). Menos se conoce sobre los efectos del entrenamiento en la reducción de los componentes del síndrome

metabólico. En uno de los estudios que incluía una intervención en pacientes con síndrome metabólico (Katzmarzyk et al. 2003) se demostró que a pesar de no producirse una pérdida significativa de peso, el 31% de los participantes dejaron de ser clasificados con síndrome metabólico después de 22 semanas de entrenamiento.

La oxidación máxima de grasa como método para evaluar los efectos del ejercicio sobre el síndrome metabólico

El término "fitness" metabólico se reconoce actualmente en la literatura científica. A pesar de no estar bien definido aún, se asocia con cambios positivos en el perfil lipídico, la presión arterial y la sensibilidad a la insulina (Tremblay et al. 1999); cuanto más cercano a los valores fisiológicos normales de un individuo sano, mejor es el fitness metabólico. Una nueva variable que se está midiendo para el estudio del fitness metabólico es la capacidad para oxidar grasa durante el ejercicio (Helge et al. 2008). Esta variable ha surgido al observar que durante un periodo de entrenamiento a moderada intensidad (ejercicio para la salud) algunas veces no se producen incrementos del consumo máximo de oxigeno (Bouchard et al. 1992). Este es el caso del estudio de Helge y colaboradores (Helge et al. 2008) en donde el consumo máximo de oxigeno no cambió después de 33 días entrenando esquí de fondo mientras que su capacidad máxima para oxidar grasa aumentó notablemente..

Por tanto, la oxidación máxima de grasa durante el ejercicio y como ésta se desplaza hacia intensidades más bajas con el entrenamiento da una idea de la flexibilidad metabólica (i.e. adaptabilidad) del sistema ante la influencia del entrenamiento físico. Este índice ha sido estudiado con éxito en trabajos que incluían obesos y diabéticos Tipo 2 (Ara et al. 2011; Larsen et al. 2009). No está todavía del todo claro si los sujetos con alguna alteración desde el punto de vista metabólico (i.e. obesos y/o pacientes con síndrome metabólico) tienen una capacidad reducida para oxidar grasas durante el ejercicio (Ara et al. 2011; Larsen et al. 2009) y si el entrenamiento podría normalizar la capacidad de oxidar grasas durante el ejercicio en esta población. En el presente proyecto, utilizaremos por primera vez la oxidación máxima de grasas durante el ejercicio en pacientes con síndrome metabólico como índice de las adaptaciones metabólicas derivadas de un programa de ejercicio.

Duración y tipo de ejercicio para el tratamiento del síndrome metabólico

Esreconocido que el ejercicio aeróbico puede ser beneficioso para tratar el síndrome metabólico en hombres y mujeres, alcanzando en algunos estudios hasta un 35% de éxito terapéutico (Katzmarzyk et al. 2003), utilizando un entrenamiento de 20 semanas de duración. No obstante, la duración óptima de un programa de ejercicio para alcanzar beneficios en cada uno de los componentes del síndrome metabólico no ha sido todavía establecida y este es uno de los objetivos centrales del presente estudio. De acuerdo con Durstine y colaboradores (Durstine et al. 2002) no existe prácticamente ningún cambio en los niveles de HDL-c relacionados con el ejercicio en un programa más corto de 12 semanas pero cuando dicho programa se extiende más allá de este tiempo, los incrementos en el HDL son más probables.

A partir de un meta-análisis donde se analizan los efectos del ejercicio en relación a la pérdida de peso (Boule et al. 2001), se puede concluir que los programas de ejercicio diseñados para conseguir una pérdida significativa de peso deben tener una duración mayor de 12 semanas. Uno de los estudio incluidos (Dunstan et al. 1998) en el mencionado meta-análisis reportó cambios en el área bajo la curva de la insulina después de 8 semanas de entrenamiento de pesas en circuito, sin cambios en los niveles de glucemia e insulinemia en ayunas o hemoglobina glucosilada (i.e., HbA1c). En el año 2010 un estudio publicó que un entrenamiento

de 12 semanas (120 minutos de ejercicio a la semana), en pacientes con síndrome metabólico no produjo reducción en el peso corporal y como consecuencia, los pacientes no mejoraron su metabolismo de la glucosa (Stensvold et al. 2010). No obstante, los participantes redujeron su circunferencia de cintura y mejoraron su función endotelial medida por la dilatación referente al flujo. De otro lado, otros estudios no han podido documentar efectos en la función endotelial a las 24 semanas de una modificación en el estilo de vida que incluía ejercicio en pacientes con síndrome metabólico (Aizawa et al. 2009).

En resumen, de los datos disponibles procedentes de estudios previos, no está del todo claro qué tipo de ejercicio (entrenamiento aeróbico o entrenamiento de fuerza) y cuál debe ser la duración de un programa de entrenamiento que provoque un efecto en los componentes del síndrome metabólico. Parece que una régimen con una duración superior a las 12 semanas y con una frecuencia superior a los 120 minutos a la semana es necesario para que algunos delos componentes del síndrome metabólico se vean modificados. Más aun, parece que la combinación de un programa de entrenamiento combinado (aeróbico y fuerza) es tan efectivo como únicamente el entrenamiento aeróbico (Stensvold et al. 2010). Teniendo en cuenta la información previamente citada, surge el diseño del presente estudio que se detallará en la sección de métodos.

Referencias

Aizawa K, Shoemaker JK, Overend TJ, Petrella RJ (2009) Metabolic syndrome, endothelial function and lifestyle modification. Diab Vasc Dis Res 6: 181-189

Alberti KG, Zimmet P, Shaw J (2006) Metabolic syndrome-a new world-wide definition. A consensus statement from the International Diabetes Federation. Diabet Med 23: 469-480

Ara I, Larsen S, Stallknecht B, Guerra B, Morales-Alamo D, Andersen JL, Ponce-Gonzalez JG, Guadalupe-Grau A, Galbo H, Calbet JAL, Helge JW (2011) Normal mitochondrial function and increased fat oxidation capacity in leg and arm muscles in obese humans. International Journal of Obesity , 35: 99-108

Ballor DL, Katch VL, Becque MD, Marks CR (1988) Resistance weight training during caloric restriction enhances lean body weight maintenance. Am J Clin Nutr 47: 19-25

Bergstrom J (1962) Muscle electrolytes in man. Scand J Clin Lab Invest [Suppl] 14: 1-110

Blair SN, Brodney S (1999) Effects of physical inactivity and obesity on morbidity and mortality: current evidence and research issues. Med Sci Sports Exerc 31: S646-662

Bouchard C, Dionne FT, Simoneau JA, Boulay MR (1992) Genetics of aerobic and anaerobic performances. Exerc Sport Sci Rev 20: 27-58

Boule NG, Haddad E, Kenny GP, Wells GA, Sigal RJ (2001) Effects of exercise on glycemic control and body mass in type 2 diabetes mellitus: a meta-analysis of controlled clinical trials. JAMA 286: 1218-1227

Caspersen CJ (1987) Physical inactivity and coronary heart disease. Physician and Sportsmedicine 15: 43-44

Deen D (2004) Metabolic Syndrome: Time for Action. Am Fam Physician 69: 2887-2888

Dunstan DW, Puddey IB, Beilin LJ, Burke V, Morton AR, Stanton KG (1998) Effects of a short-term circuit weight training program on glycaemic control in NIDDM. Diabetes Res Clin Pract 40: 53-61

Durstine JL, Grandjean PW, Cox CA, Thompson PD (2002) Lipids, lipoproteins, exercise. J Cardiopulm Rehabil 22: 385-398

Durstine JL, Haskell WL (1994) Effects of exercise training on plasma lipids and lipoproteins. Exer Sport Sci Rev 22: 447

Frayn KN (1983) Calculation of substrate oxidation rates in vivo from gas exchange. J Appl Physiol 55: 628-634

Frayn KN, Maycock PF (1980) Skeletal muscle triaglycerol in the rat: methods for sampling and measurement, and studies of biological variability. J Lipid Res 21: 139-144

Gami AS, Witt BJ, Howard DE, Erwin PJ, Gami LA, Somers VK, Montori VM (2007) Metabolic syndrome and risk of incident cardiovascular events and death: a systematic review and meta-analysis of longitudinal studies. J Am Coll Cardiol 49: 403-414

Helge JW, Damsgaard R, Overgaard K, Andersen JL, Donsmark M, Dyrskog SE, Hermansen K, Saltin B, Daugaard JR (2008) Low-intensity training dissociates metabolic from aerobic fitness. Scand J Med Sci Sports 18: 86-94
Hildrum B, Mykletun A, Hole T, Midthjell K, Dahl A (2007) Age-specific prevalence of the metabolic syndrome defined by the International Diabetes Federation and the National Cholesterol Education Program: the Norwegian HUNT 2 study. BMC Public Health 7: 220
Horowitz JF, Mora-Rodriguez R, Byerley LO, Coyle EF (1997) Lipolytic suppression following carbohydrate ingestion limits fat oxidation during exercise. Am J Physiol 273: E768-775
Horowitz JF, Mora-Rodriguez R, Byerley LO, Coyle EF (1999) Substrate metabolism when subjects are fed carbohydrate during exercise. Am J Physiol 276: E828-835.
Hwang LC, Bai CH, Chen CJ, Chien KL (2007) Gender difference on the development of metabolic syndrome: a population-based study in Taiwan. Eur J Epidemiol 22: 899-906
INE (2006) Encuesta Nacional de Salud; Instituto Nacional de Estadística.
Jackson AS, Pollock ML (1978) Generalized equations for predicting body density of men. Br J Nutr 40: 497-504
Jackson AS, Pollock ML, Ward A (1980) Generalized equations for predicting body density of women. Med Sci Sports Exerc 12: 175-181
Kashyap SR, De Fronzo RA (2007) The insulin resistance syndrome: physiological considerations. Diab Vasc Dis Res 4: 13-19
Katzmarzyk PT, Leon AS, Wilmore JH, Skinner JS, Rao DC, Rankinen T, Bouchard C (2003) Targeting the metabolic syndrome with exercise: evidence from the HERITAGE Family Study. Med Sci Sports Exerc 35: 1703-1709
Kiernan M, King AC, Stefanick ML, Killen JD (2001) Men gain additional psychological benefits by adding exercise to a weightloss program. Obes Res 9: 770-777
Larsen S, Ara I, Rabøl R, Andersen JL, Boushel R, Dela F, Helge JW (2009) Are substrate use during exercise and mitocondrial respiratory capacity decreased in arm and leg muscle in type 2 diabetes? Diabetologia 52: 1400-1408
Lillioja S, Bogardus C, Mott DM, Kennedy AL, Knowler WC, Howard BV (1985) Relationship between insulin-mediated glucose disposal and lipid metabolism in man. J Clin Invest 75: 1106-1115
Lopez-Candales A (2001) Metabolic syndrome X: a comprehensive review of the pathophysiology and recommended therapy. J Med 32: 283-300
McArdle WD, Katch FI, Katch VL (1996) Physical activity, health and aging. In: Balado D (ed) Exercise Physiology; energy, nutrition and human performance. Williams and Wilkins, Baltimore, pp. 635-655
Mikines K, Sonne B, Farrell P, Tronier B, Galbo H (1988a) Effect of physical exercise on sensitivity and responsiveness to insulin in humans. Am J Physiol 254: E248-E259
Mikines KJ, Sonne B, Farrell PA, Tronier B, Galbo H (1988b) Effect of physical exercise on sensitivity and responsiveness to insulin in humans. Am J Physiol 254: E248-259
Milesis CA, Pollock ML, Bah MD, Ayres JJ, Ward A, Linnerud AC (1976) Effects of different durations of physical training on cardiorespiratory function, body composition, and serum lipids. Res Q 47: 716-725
Paniagua JA, de la Sacristana AG, Romero I, Vidal-Puig A, Latre JM, Sanchez E, Perez-Martinez P, Lopez-Miranda J, Perez-Jimenez F (2007) Monounsaturated Fat-Rich Diet Prevents Central Body Fat Distribution and Decreases Postprandial Adiponectin Expression Induced by a Carbohydrate-Rich Diet in Insulin-Resistant Subjects. Diabetes Care %R 102337/dc06-2220 30: 1717-1723
Passonneau JV, Lauderdale VR (1974) A comparison of three methods of glycogen measurement in tissues. Anal Biochem 60: 405-412
Perseghin G, Price TB, Petersen KF, Roden M, Cline GW, Gerow K, Rothman DL, Shulman GI (1996) Increased Glucose Transport-Phosphorylation and Muscle Glycogen Synthesis after Exercise Training in Insulin-Resistant Subjects. N Engl J Med 335: 1357-1362
Position stand (1993) Physical activity, physical fitness, and hypertension. Med Sci Sports Exerc 25: i-x
Reaven G (2006) The metabolic syndrome: is this diagnosis necessary? Am J Clin Nutr 83: 1237-1247
Reaven GM (1988) Role of insulin resistance in human disease. Diabetes 1595-1607
Reaven GM (1995) Characteristics of metabolic syndrome. Endocrinol Metab 2: 37-42
Ren JM, Semenkovic CF, Gulve EA, Gao J, Holloszy JO (1994) Exercise induces rapid increases in GLUT4 expression, glucose transport capacity, and insulin-stimulated glycogen storage in muscle J Biol Chem 269: 14396-14401
Sady SP, Cullinane EM, Saritelli A, Bernier D, Thompson PD (1988) Elevated high-density lipoprotein cholesterol in endurance athletes is related to enhanced plasma triglyceride clearance. Metabolism 37: 568-572
Sánchez-Chaparro M, Calvo-Bonacho E, González-Quintela A, Fernández-Labandera C, Cabrera M, Sáinz J, Fernández-Meseguer A, Banegas J, Ruilope L, Valdivielso P, Román-García J, Group ICRAIS (2008) Occupation-related differences in the prevalence of metabolic syndrome. Diabetes Care 31: 1884-1885

Stensvold D, Tjønna AE, Skaug EA, Aspenes S, Stølren T, Wisløff U, Slørdahl SA (2010) Strength training versus aerobic interval training to modify risk factors of metabolic syndrome. J Appl Physiol 108: 804-810
Tremblay A, Doucet E, Imbeault P, Mauriège P, Després JP, Richard D (1999) Metabolic fitness in active reduced-obese individuals. Obes Res 7: 556-563
Tremblay A, Fontaine E, Poehlman ET, Mitchell D, Perron L, Bouchard C (1986) The effect of exercise-training on resting metabolic rate in lean and moderately obese individuals. Int J Obes 10: 511-517
Tura A, Sbrignadello S, Succurro E, Groop L, Sesti G, Pacini G An empirical index of insulin sensitivity from short IVGTT: validation against the minimal model and glucose clamp indices in patients with different clinical characteristics. Diabetologia 53: 144-152
Venables MC, Jeukendrup AE (2008) Endurance training and obesity: effect on substrate metabolism and insulin sensitivity. Med Sci Sports Exerc 40: 495-502
Wilson PW, D'Agostino RB, Parise H, Sullivan L, Meigs J (2005) Metabolic syndrome as a precursor of cardiovascular disease and type 2 diabetes mellitus. Circulation 112: 3066-3072
Wu T, Gao X, Chen M, van Dam RM (2009) Long-term effectiveness of diet-plus-exercise interventions vs. diet-only interventions for weight loss: a meta-analysis. Obes Rev 10: 313-323
Zuti WB, Holding LA (1976) Comparing diet and exercise as weight reduction tools. Physician and Sportsmedicine 4: 49-53

FINALIDAD DEL PROYECTO, JUSTIFICACIÓN.
Pretendemos que los resultados de este estudio sean de aplicación a políticas de salud regional y nacional para prevenir el síndrome metabólico, claramente asociado con morbi-moralidad por enfermedad cardiovascular y diabetes mellitus. Los resultados pueden ser aplicados para prevenir o mitigar el desarrollo de la resistencia a la insulina en adultos con sobrepeso a través de cambios conductuales como es incorporar ejercicio físico dentro del estilo de vida.

Objetivos
El objetivo principal es determinar el efecto del ejercicio continuado y estructurado (entrenamiento físico) en los cuatro componentes (obesidad abdominal, dislipidemia, resistencia a la insulina e hipertensión) del síndrome metabólico.

Los objetivos específicos son:

1. Identificar en una escala temporal durante 4 meses, los efectos del entrenamiento físico en la reversión de cada uno de los componentes del síndrome metabólico: i) obesidad central (abdominal), ii) dislipidemia aterogénica, iii) trastorno en el metabolismo de los carbohidratos y iv) disfunción vascular.

2. Evaluar la tasa de reaparición (recidiva) de cada uno de los componentes del síndrome metabólico hasta 6 meses después de haber suspendido el programa de ejercicio supervisado.

3. Evaluar si el síndrome metabólico se constituye como un factor que limita o retrasa algunas de las adaptaciones al entrenamiento con ejercicio observadas en un grupo de personas pareado por edad, género, actividad física, composición corporal y aptitud física cardiorespiratoria. (i.e., evaluadas según el consumo de oxígeno máximo VO_{2max}).

4. Determinar si la pérdida de peso lograda a través de 4 meses por medio de ejercicio supervisado es más efectiva en el tratamiento del síndrome metabólico, que la obtenida mediante restricción calórica sin ejercicio.

5. Determinar las adaptaciones que provocan 4 meses de intervencion basada en ejercicio en el fitness metabolico (i.e. oxidacion maxima de grasas)

Las hipótesis son:

- Nuestra principal hipótesis es que teniendo en cuenta que en la mayoría de las personas los diferentes componentes del síndrome metabólico (obesidad abdominal, dislipidemia, resistencia a la insulna e hipertensión) aparecen de forma ordenada (Hwang et al. 2007)), de la misma manera desaparecerán ordenadamente en relación a las adaptaciones derivadas del entrenamiento físico.

- Asimismo la progresion de la reaparición de los componentes será ordenada y esto nos dará pistas sobre cuales son los componentes mas modificables para poder incidir sobre estos.

- Adicionalmente hipotetizamos que para una pérdida de peso similar, conseguida por una restricción calórica o secundaria a un aumento en el gasto energético mediante ejercicio, este último será más efectivo en el tratamiento del síndrome metabólico.

METODOS

Tipo de investigación

Estudio experimental (aleatorizado)

Estrategia de reclutamiento

Los participantes de la presente investigación se reclutarán a través de avisos en los medios de comunicación locales (periódicos y radio), a través de los cuales se solicitarán voluntarios con sobrepeso según el índice de masa corporal (IMC<25). De este gran grupo de posibles participantes que respondan a los anuncios, se reclutarán 60 hombres y mujeres sedentarios con edades entre los 25 y 65 años que tengan criterios para Síndrome Metabólico (SinMet). Adicionalmente se reclutarán 60 personas sedentarias pero que no cumplan criterios para SinMet (Controles) quienes serán pareados con los participantes del grupo SinMet por género, edad, perímetro de cintura, composición corporal y aptitud física cardiorespiratoria (evaluada mediante una prueba de ejercicio incremental y la estimación del consumo de oxígeno pico VO_{2pico})

Criterios de inclusión y exclusión

El síndrome metabólico será definido de acuerdo a los criterios publicados por la Federación Internacional de Diabetes: perímetro de cintura >94 cm para hombres y >80 para mujeres de etnia caucásica, más dos de los siguientes 3 factores: cifras altas de presión arterial (>130 mmHg para la sistólica y >85 para la diastólica), glucemia alta en ayunas (>100 mg/dl), dislipidemia: (triglicéridos >150 mg/dl, HDL <40 mg/dl) (Alberti, Zimmet et al. 2006). Los participantes que se encuentre en tratamiento para hipertensión o que reciban medicamentos hipoglucemiantes o hipolipemiantes se considerarán como poseedores del respectivo factor de inclusión. Todos los sujetos deberán ser físicamente inactivos, al menos durante el año anterior. Adicionalmente no deberán tener diagnóstico de alguna patología endocrina diferente a intolerancia a los carbohidratos y sus niveles hormonales (excepto la insulina) deberán encontrarse en rangos de normalidad clínica. Otros criterios de exclusión serán: cirugía reciente, enfermedad cardiovascular (especialmente enfermedad coronaria, enfermedad valvular cardiaca, insuficiencia cardiaca, arritmias ventriculares complejas, enfermedades con compromiso renal, hepático, respiratorio o neuromuscular. Los participantes serán informados de manera verbal y escrita de los riesgos y beneficios referentes a la participación en el estudio y brindarán su consentimiento escrito. Los participantes mantendrán supervisión por sus médicos de familia quienes ajustarán sus tratamientos de acuerdo a las necesidades particulares de cada individuo; dichas modificaciones serán informadas al grupo investigador.

Razones para no excluir mujeres en el estudio

No existen suficientes estudios que hayan evaluado las diferencias entre sexos en relación al desarrollo y el tratamiento del síndrome metabólico. A pesar de las similar prevalencia entre géneros cuando se estudian individuos de la misma edad, los hombres normalmente muestran un mayor porcentaje de hiperglucemia, hipertensión e hipertrigliceridemia. En contraste, las mujeres, normalmente muestran una mayor prevalencia de obesidad central y bajas concentraciones de HDL-c (Bo, Gentile et al. 2005). Más aún, parece que las mujeres tienden a aislar el primer componente antes y presentan el síndrome metabólico más tarde que el hombre (Hwang, Bai et al. 2007). De la misma manera que existen diferencias entre hombres y mujeres en el desarrollo de síndrome metabólico, la escala temporal en la que el ejercicio puede corregir los defectos asociados a los componentes del síndrome metabólico también

pueden diferir entre hombres y mujeres. Datos del estudio Fels (Remsberg, Rogers et al. 2007) muestran que el incremento total en la práctica deportiva y la actividad física redujeron los componentes del síndrome metabólico en hombres pero no tan claramente en mujeres. Sin embargo, Katzmarzyk y colsl. argumentan lo contrario, (Katzmarzyk, Leon et al. 2003). Creemos que no existen datos suficientes para concluir que las mujeres con síndrome metabólico responderán de forma diferentes al entrenamiento que sus coetáneos varones. Por esta razón hemos decidido incluir participantes de ambos géneros. Este hecho nos permitirá analizar posteriormente si existen diferencias de género como consecuencia de los programas de entrenamiento.

Agrupamiento de participantes y cálculo del tamaño muestral.

Los participantes se estratificarán por edad y medicación recibida. De acuerdo a los criterios de inclusión previamente descritos serán asignados al grupo de síndrome metabólico o grupo control. Cada uno de los dos grupos será aleatoriamente asignado a una de dos intervenciones: Entrenamiento físico o Restricción calórica con sedentarismo. Como resultado de lo anterior existirán 4 grupos identificados con sus respectivos acrónimos (i.e., MetSyn-Ex; MetSyn-Cr; Con-Ex; Con-Cr (tabla 1.)). Hemos incluido los grupos de restricción calórica en un intento de alcanzar la pérdida de peso que consigan los sujetos de los grupos de ejercicio en el grupo de participantes que permanecerán sedentarios, ya que la pérdida de peso se constituye como un factor de confusión en el análisis.

Cada uno de los grupos (MetSyn-Ex; MetSyn-Cr; Con-Ex; Con-Cr) estará compuesto por 30 sujetos teniendo en cuenta que el análisis de potencia de un estudio en el que se usaron programas de ejercicio similares (3 meses de duración) en sujetos similares (Stensvold, Tjønna et al. 2010) indica que para observar efectos significativos en los componentes del síndrome metabólico era necesario 27 participantes en cada grupo con lo que proponemos reclutar 30 para contar con un 10% de falta de adherencia al programa de ejercicio o dieta (Vincent 1999).

Tabla 1. Grupos experimentales

GRUPO CONTROL (n=60)		GRUPO“METSYN” (n=60)	
ENTRENAMIENTO FÍSICO	RESTRICCIÓN CALÓRICA + SEDENTARISMO	ENTRENAMIENTO FÍSICO	RESTRICCIÓN CALÓRICA + SEDENTARISMO
**Con-Ex** (n=30)	**Con-Cr** (n=30)	**MetSyn-Ex** (n=30)	**MetSyn-Cr** (n=30)

Intervención con entrenamiento físico

El programa de entrenamiento consistirá en 3 sesiones semanales de ejercicio aeróbico interválico (EIA) y 1 sesión semanal de entrenamiento de fuerza (EF). EIA consistirá en un trabajo sobre ciclo-ergómetro (o carrera en un tapiz rodante) que incluya un periodo de 10 min de calentamiento al 70% de la FC_{pico} seguida de 5 intervalos de 4 minutos al 90-95% de su FC_{pico} intercalados con periodos de 3 minutos de recuperación activa al 70% de la FC_{pico}, con un enfriamiento de 10 minutos, alcanzando un total de 55 min de duración por entrenamiento.

Tendiendo en cuenta los efectos del ritmo circadiano sobre la frecuencia cardiaca, los entrenamientos se llevaran a cabo a la misma hora del día y la carga será ajustada diariamente para provocar la FC indicada. Como consecuencia de las adaptaciones producidas por el ejercicio la carga de entrenamiento deberá ser ajustada para llegar a frecuencias cardiacas similares con el transcurrir del tiempo (Lucía, Hoyos et al. 2000) y de esta manera cumplir con el principio de sobrecarga para el entrenamiento físico.

Las sesiones de EF consistirán en sesiones semanales de fortalecimiento enfocadas en miembros superiores, tronco y miembros inferiores. Los participantes ejecutarán un breve calentamiento llevando a a cabo ejercicios de fortalecimiento de la zona abdominal y dorso durante 10 min. Durante la primera semana cada ejercicio se ejecutará al 60% de la repetición máxima individual (i.e. 1-RM) y se programarán tres series de 8 a 12 repeticiones en cada sesión. A partir de la segunda semana y hasta el final del estudio se mantendrán el volumen pero la intensidad se aumentará hasta el 80% de 1-RM. Cada mes, 1-RM será evaluada y las cargas re-ajustadas. El circuito incluirá 3 estaciones para el tren superior (prensa en banca, prensa de hombros militar y remo en posición sentado) y 3 ejercicios para el tren inferior (Sentadilla, extensión de piernas y curl de piernas sentado). El número de repeticiones multiplicado por el peso será recogido y almacenado para la evaluación de las adaptaciones del entrenamiento. El tiempo total de las sesiones de entrenamiento de fuera será de aproximadamente 50 min. El tiempo total de entrenamiento a la semana será de 4 sesiones de ejercicio (3 de EIA y 1 de EF) que sumarán aproximadamente 210 min de actividad física moderada – vigorosa.

Control nutricional

Durante la semana previa al inicio de las intervenciones, todos los participantes llevarán un registro escrito de su peso matutino (completamente desnudos) y de todos los alimentos consumidos con sus respectivos pesos. La energía total y la ingesta de macronutrientes será evaluado a través del registro individual de cada participante utilizando un software adaptado sobre los hábitos alimenticios de los españoles (Programa de Cálculo Nutriconal CESNID V 1.0 – Centre d`Ensenyament Superior de Nutrició I Dietètica adscrito al a Universitat de Barcelona). Con estos datos se establecerá la ingesta calórica habitual que:

a) Se mantendrá durante los 4 meses de intervención en los grupos de ejercicio y
b) Será reducida en una cantidad similar al gasto energético inducido por la intervención con ejercicio en los participantes del grupo restricción calórica y sedentarismo.

Se pretende que el gasto calórico de cada sesión de ejercicio se encuentre entre 525-875 kcal o 2100-3500 kcal semanales y que genere una pérdida de peso entre 0.25-0.35 kg por semana, para un total de 4-5.6 kg durante toda la intervención. Para ajustar la pérdida de peso en el grupo de restricción calórica, los participantes reducirán su ingesta energética en 300-500 kcal/día (2100-3500 kcal/semana). Con el propósito de alcanzar los objetivos de dieta y peso corporal, los participantes serán supervisados semanalmente. Todos los participantes del estudio mantendrán un registro de su ingesta y de su peso corporal durante dos días a la semana (uno laborables y uno de fin de semana) y presentarán al grupo de investigación los datos para ser analizados y realizar los ajustes necesarios.

Procedimiento de evaluación

Los sujetos deberán ser evaluados durante dos días consecutivos en el laboratorio en ocho ocasiones diferentes:

a) antes del inicio del entrenamiento-dieta (día 1),
b) después de la primera semana de entrenamiento (+ 1 sem)
c) después de 1 mes de intervención (+ 1 m)
d) después de 2 meses de intervención (+ 2 m)
e) después de 3 meses de la intervención (+ 3 m)
f) después de 4 meses de intervención (+ 4 m)
g) 1 mes posterior a la terminación de la intervención (para evaluar recaída)
h) 6 meses después de terminar la intervención (para evaluar recaída)

Durante el primer día de evaluación los participantes realizaran una batería de test que se les administrara por la mañana, en ayunas siendo necesario que las 24 horas anteriores a la evaluación no hayan realizado ningún tipo de entrenamiento físico ni hayan ingerido alcohol y/o café. Durante el segundo día y también en ayunas, se evaluara el fitness metabólico de cada sujeto.

DIA 1 de TEST

Absorciometría de emisión Dual-de rayos X (DEXA)

La composición corporal del cuerpo se llevara a cabo mediante DEXA (Hologic Discovery-W, Hologic Corp., Waltham, MA, USA) de la forma que descrita en otras publicaciones (Calbet, Moysi et al. 1998). La masa magra (g), la masa grasa (g) y el contenido mineral óseo (g) se calcularán a partir de una prueba de cuerpo completo. El análisis de cuerpo completo se complementará con una evaluación regional de la composición corporal en extremidades y tronco. A través de este análisis la grasa y la masa magra regional se pueden obtener con un coeficiente de variación inferior al 5%

Medidas antropométrica y presión arterial en reposo

El peso y la talla serán evaluadas utilizando una bascula estándar y un estadiómetro que permite medir con una precisión de 0.1 cm y kg respectivamente (Seca, Vogel & Halke Hamburg, Germany). El índice de masa corporal (IMC; en kg/m^2) será calculado utilizando la talla y el peso del sujeto. La circunferencia de cintura (ubicando en el plano horizontal el punto medio entre el reborde costal y las crestas iliacas) será medida utilizando una cinta de medir de plástico con precisión de 0.1 cm. Para la medición de la presión arterial se acomodará a los participantes en posición supina durante 15 min. A continuación se determinará la presión arterial sistólica (PAS) y la diastólica (PAD) con la ayuda de un esfigmomanómetro aneoroide (Heine Gamma 4. Germany). La media de las ultimas 2-3 mediciones será utilizada como valor de la PAS y la PAD.

Metabolismo basal en reposo (RMR)

El consumo de oxigeno pulmonar (VO_2) y de dióxido carbónico (VCO_2) serán medidos durante el ejercicio y en reposo (metabolismo basal en reposo) utilizando un sistema automático en linea (Oxycon Pro; Jaeger, Würzburg, Germany). Antes de cada test se llevará a cabo una calibración del volumen y una calibración de los gases utilizando gases de concentración conocida. El metabolismo basal en reposo (RMR) será evaluado mediante calorimetría indirecta. Los participantes deberán estar en ayunas (12h) y estar en reposo en posición supina en una

cama confortable con su cabeza en un sistema tipo tienda de Plexiglas durante 35 min. El RMR se llevara a cabo de forma computarizada y utilizando un circuito abierto con un sistema tipo tienda ventilado utilizando los datos de los últimos 15 minutos para permitir una aclimatación. La habitación deberá estar en calma y con la luz ligeramente apagada. La mujeres que no sean amenorreicas serán evaluadas siempre en el mismo momento del ciclo menstrual.

Test de tolerancia a la glucosa intravenoso

Para evaluar las mejoras en la sensibilidad a la insulina utilizaremos un test de toleración a la glucosa intravenoso de 60 minutos de duración (Tura, Sbrignadello et al. 2010). Los participantes llegarán al laboratorio después de 12 horas de ayunas y con 48h de restricción de ejercicio asegurando haber tomado una dieta rica en hidratos el día anterior. Después de al menos 10 minutos de reposo en posición supina, se insertará un catéter intravenoso (Becton Dickinson S.A. Madrid, España) en la vena antecubital. A continuación, se extraerá una muestra basal, seguida de una infusión manual de 3 min de una dosis de 0.5g por kilogramos de peso de una solución del 30% de glucosa (Grifols, Barcelona, España). En los sujetos con pesos por encima de 70 kg, 35 g de glucosa serán infundidos como máximo de acuerdo a las recomendaciones del Grupo ICARUS (McCulloch, Bingley et al. 1993). Después de terminar la infusión se extraerán cinco mililitros de sangre en los momentos 0, 2, 4, 6, 8, 10, 20, 30, 40, 50 y 60-min. Para el cálculo de la respuesta aguda de la insulina, el área bajo la curva de las primeras seis muestras se tendrán en cuenta. El índice de sensibilidad a la insulina (CS_I) será calculado siguiendo las indicaciones de Tura y cols.

$$CS_I = \alpha * [K_G / (\Delta AUC_{INS} / T)]$$

Donde α es un factor escalar (0.276), K_G es la tasa de desaparición de la glucosa utilizando una pendiente logarítmica de la glucosa, ΔAUC_{INS} es el área bajo la curva de la concentración de insulina sobre la concentración basal y T es el intervalo de tiempo entre las muestras del minuto 10 y 50.

Análisis de química sanguínea

Después de un decúbito supino de 15 minutos y previo a la ejecución del IVGTT se extraerán 10 ml de sangre venosa para el análisis de metabolitos. El plasma tratado con Citrato y EDTA se centrifugará a 3000 rpm durante 10 min a 4°C.. Se guardarán alícuotas de plasma a -80°C para ser analizadas posteriormente. Las concentraciones séricas de Triglicéridos, glucosa, HDL-C, colesterol total, hemoglobina glicada (HbA1c), TNF-alfa, IL-6, leptina, proteína C reactiva y adiponectina serán medidas usando procedimientos estándar. Los niveles séricos de adiponectina, leptina, IL-6, IL-8, HbA1c, Proteína C reactiva y TNF-alfa se medirán por ELISA de alta sensibilidad específica para humanos. Para la medición de triglicéridos, colesterol total, HDL-C, glucosa se usarán análisis colorimétricos en un lector de placas (Versamax, Molecular Devices, USA) usando ensayos debidamente validados. Los Triglicéridos:serán medidos usando un método de glicerol fosfato oxidasa/peroxidasa (BioSystems, España) con un coeficiente de variación (CV) de 1.7%. El Colesterol total será medido usando un reactivo de colesterol oxidasa/peroxidasa (BioSystems, España) con un CV de 1,0%. La fracción HDL-C:se medirá por el método de Fosfotungstato/Mg-Colesterol oxidasa/peroxidasa (BioSystems, España) con un CV de 3,2%. La glucosa será medida usando como reactivo glucosa oxidasa (Thermo scientific, EE.UU.) con un CV de3,0%. La insulin será medida usando un metalo-inmunoanálisis de carbonilo (CMIA; Architect i2000SR Abbott Lab). Los ácidos grasos libres se medirán con el kit

de reactivos (HR series NEFA, WAKO, EE.UU) con un CV de 4,1%. Para los inmunoanálisis se usarán los siguientes ensayos de alta sensibilidad específicos para humanos: Adiponectina (Linco Research, EE.UU.) con un CV de 3.9%. Leptina (R&D Systems, EE.UU) con un CV de 3.2%. IL-6 (Quantikine HS600; R&D Systems Europa) con un CV de 5.5% . TNF-alfa (Quantikine HSTA00C; R&D Systems Europa) con un CV de 3.5%. La Proteina C reactiva y la HbA1c se medirán usando kits de alta sensibilidad debidamente validados (Gentaur Molecular Products, Bélgica y Kalon Biological Limited, Reino Unido; respectivamente).

Día 2 de pruebas.

Economía del movimiento, consumo máximo de O_2 y máxima oxidación de grasas.

El pico en el consumo de O_2 ($VO_{2\ pico}$) será medido durante un protocolo de ejercicio en rampa, que se individualizará y ejecutará en un cicloergómetro para el que se usará un sistema abierto de calorimetría indirecta (Oxycon Pro; Jaeger, Würzburg, Germany). Después de un calentamiento de 10-min at 50 vatios, la carga se ajustará a otro nivel submáximo constante por otros 10 minutos. La captación de O_2 en $ml \cdot kg^{-1} \cdot min^{-1}$ y la Frecuencia cardiaca (FC) en latidos por minuto (lpm) durante la carga submáxima se usarán para calcular la economía del movimiento y las adaptaciones cardiovasculares al entrenamiento. Los participantes ejecutarán 3-4 cargas adicionales de 3 minutos de duración para determinar la oxidación de grasas y la intensidad (% del VO2pico) en la que se oxida la máxima cantidad de grasas (FatMax en inglés). Completado este procedimiento, la carga se incrementará 25 vatios por minuto hasta la fatiga. La media de las tres medidas más altas será usada para establecer el $VO_{2\ pico}$. La FC se medirá durante la prueba (Polar electroRS400, Finlandia), y la maxima FC alcanzada se anotará como (FC_{pico}).

Prueba de fuerza 1 RM

La fuerza máxima será determinada por 1-RM en los 6 ejercicios en los cuales entrenarán los participantes. 1-RM se definirá como la máxima carga que el participante es capaz de levantar ejecutando un rango de movimiento completo. Teniendo en cuenta que este test es fatigante y por el esfuerzo máximo aumenta el riesgo de lesion, usaremos un método de extrapolación para el cálculo de 1-RM usando cargas submáximas y midiendo las velocidades de movilización usando un transductor de señales(Sanchez-Medina, Perez et al. 2010). Consideramos que la evaluación de la fuerza máxima es importante, ya que un estudio reciente de uno de los investigadores de este grupo sugiere que la fuerza muscular está más asociada a una adecuada condición cardio-metabólica que a la aptitud física *per se* (Solera Martínez, López Martínez et al. 2011).

Calidad de vida

La calidad de vida relacionada con la salud (HRQL por sus siglas en inglés) será medida usando versiones validadas de la traducción al español del cuestionario de salud SF-36 (Martínez-González, López-Fontana et al. 2005). El cuestionario será administrado una semana antes de la intervención y durante la última semana de esta. Con esta herramienta se exploran 8 dimensiones de la salud usando escalas de nueve elementos Únicamente se usarán en este estudio las dimensiones que exploran la salud física (funcionamiento físico, limitaciones de rol, salud general y transiciones de salud).

Tabla 2. Secuencia de pruebas experimentales

Antes de la intervención		Pruebas para medir adaptaciones al ejercicio.				Pruebas para medir recaídas	
24-48 h	1 semana	1 mes	2 meses	3 meses	4 meses	1 mes después de terminar la intervención	6 meses después de terminar la intervención

En las ocho ocasiones señaladas, durante dos días consecutivos, mediremos en todos los participantes tanto los 5 componentes del síndrome metabólico (obesidad central medida por el perímetro de cintura, cifras altas de presión arterial, glucosa alta en ayunas, hiper-trigliceridemia, HDL bajo) como las adaptaciones fisiológicas al entrenamiento (masa libre de grasa, gasto energético de reposo, VO_{2max}, economía del movimiento, máxima oxidación de grasa y ganancias en fuerza). Las medidas adicionales serán adicionadas como se describe en la Tabla 4 con el propósito de expandir las conclusiones del estudio.

Tabla 3: Variables principales.

Criterios principales	Medidas
1) Obesidad central (abdominal)	Perímetro de cintura
2) Dislipidemia aterogénica	TG elevados y HDL disminuidos
3) Alteración en el metabolismo de los carbohidratos	Glucosa alta en ayuno
4) Trastorno de la función vascular	Cifras altas de presión arterial

Tabla 4. Variables adicionales

Criterios principales	Criterios adicionales	Medida
1) Obesidad central (abdominal)	Distribución anormal de la grasa	- Cantidad y distribución de la grasa corporal (DEXA)<br><br>-IMC y grasa subcutánea por pliegues de grasa<br><br>- Biomarcadores del tejido adiposo: leptina y adiponectina
2) Dislipidemia aterogénica		- ApoB<br>- Ácidos grasos libres
3) Alteración en el metabolismo de los	Disglucemia	- Test de tolerancia intravenoso a la glucosa

carbohidratos		(IVGTT)<br>- HOMA-IR
4) Trastorno de la función vascular	Estado proinflamatorio<br>Hemodinamia durante el ejercicio.	- Proteína C reactiva elevada<br>- Incremento en citoquinas pro-inflamatorias (eg TNF-alpha, IL-6)<br>- Reducción en las concentraciones de adiponectina<br>- Pulso de oxígeno (VO_2/FC)<br>- Resistencias periféricas totales (PA/Q)
5) Flexibilidad metabólica		- Máxima oxidación de grasas en el ejercicio<br>- Oxidación de grasa en reposo<br>- Umbral anaeróbico ventilatorio
6) Aptitud física		- Resistencia cardiorespiratoria (VO_2pico)<br>- Masa libre de grasa (DEXA)<br>- Fuerza de miembros superiores e inferiores
7) Cambios hormonales inducidos por estrés crónico (Bjorntorp 2001)		- Concentración plasmática de cortisol

Mediciones realizadas en biopsias musculares

Se obtendrán especímenes de tejido muscular (i.e., 20-50 mg) provenientes del vasto lateral del cuádriceps femoral, usando la técnica de biopsia con aguja.(Bergstrom 1962). Teniendo en cuenta que este es el procedimiento más invasivo del estudio y que puede limitar el interés de los participantes, solo pretenderemos recolectar muestras en 8 participantes de cada grupo (32 en total). Las muestras se obtendrán en el día anterior al inicio de la intervención y al día siguiente de finalizar la misma (4 meses de diferencia). En el tejido extraído se medirá la concentración de triglicéridos intramusculares luego de la extracción de grasa con una solución 2:1 de cloroformo y metanol y KOH al 4% en un fluorómetro (Frayn and Maycock 1980). Se analizará la composición de los triglicéridos intramusculares identificando el perfil de ácidos grasos: (C17:0 (heptadecanóico-CoA), C16:0 (palmitil-CoA), C16:1 (palmitoleoil-CoA), C18:0 (esteariol-CoA), C18:1 (oleoiol-CoA), and C18:2 (linoleiol-CoA)). Adicionalmente mediremos las concentraciones de glucógeno luego de obtener homogenización del tejido y conseguir la hidrólisis del glucógeno usando un ensayo convencional (Passonneau and Lauderdale 1974).

El grupo de investigación está igualmente interesado en medir los efectos del ejercicio en la señalización de los efectos de la insulina en pacientes con síndrome metabólico. Hipotetizamos que la acumulación tisular de intermediarios del metabolismo incompleto de las

grasas (i.e., diacilglicerol y acil co-enzima A) activa factores pro-inflamatorios (JNK, PKC, IκK/NFβκ) que alteran las señales interactuando con el receptor (IRS-1, Akt) (Schenk and Horowitz 2007). Algunos de los elementos mencionados previamente serán medidos usando técnica "Western blott".

En resumen, los lisados de células completas serán preparado como se ha descrito previamente (Schenk, Harber et al. 2009). Las muestras (25 μg) se separarán usando 8% SDS-PAGE y serán transferidas a un baño de hielo a 200 mA. Después de bloquear las membranas se realizarán pruebas para JNK fosforilado (p-JNK; no. 9251, Cell Signaling, EE.UU.), JNK-1 (sc-474; Santa Cruz Biotechnology, EE.UU.) y IκB-β (nos 9242 and 9248; Cell Signaling EE.UU). Las membranas serán posteriormente incubadas en el anticuerpo apropiado durante 60 inutos y analizadas usando quimio-luminiscencia aumentada (Amersham Biosciences, Piscataway, EE.UU). Las bandas serán cuantificadas por densitometríay (Fluor Chem SP, Alpha Innotech, EE.UU). Las densidades de las bandas para cada sujeto serán analizadas y expresadas en relación a un estándar (músculo humano) que se correrá en duplicado en todos los geles.

Análisis estadístico

Los resultados se presentarán como medias ± SD. El cambio en la media de cada grupo se presentarán como error estimado de la media (EEM) y estará soportado por los intervalos de confianza del 95% (IC 95%). Las diferencias entre grupos se considerarán como significativas cuando el IC 95% no incluya el valor de cero. (Gardner and Altman 1986). Para evaluar las diferencias entre grupos se usará un análisis de covarianza (ANCOVA), donde el tiempo y el tratamiento asignado serán los factores y los valores iniciales de las variables analizadas, covariantes (Vickers and Altman 2001). Los valores de $P < 0,05$ se considerarán como significativos. Todo el análisis estadístico se realizará usando el software SPSS (versión 19.0).

Referencias

Aizawa, K., J. K. Shoemaker, et al. (2009). "Metabolic syndrome, endothelial function and lifestyle modification." Diab Vasc Dis Res. **6**(3): 181-9.

Alberti, K. G., P. Zimmet, et al. (2006). "Metabolic syndrome-a new world-wide definition. A consensus statement from the International Diabetes Federation." Diabet Med **23**: 469-480.

Ara, I., S. Larsen, et al. (2011). "Normal mitochondrial function and increased fat oxidation capacity in leg and arm muscles in obese humans." International Journal of Obesity , **35**: 99-108.

Ballor, D. L., V. L. Katch, et al. (1988). "Resistance weight training during caloric restriction enhances lean body weight maintenance." Am J Clin Nutr **47**(1): 19-25.

Bergstrom, J. (1962). " Muscle electrolytes in man." Scand J. Clin. Lab Invest. **14**: 1-110.

Bjorntorp, P. (2001). "Heart and soul: stress and the metabolic syndrome." Scand Cardiovasc J **35**: 172-7.

Blair, S. N. and S. Brodney (1999). "Effects of physical inactivity and obesity on morbidity and mortality: current evidence and research issues." Med Sci Sports Exerc. **31**(11): S646-62.

Bo, S., L. Gentile, et al. (2005). "The metabolic syndrome and high C-reactive protein: prevalence and differences by sex in a southern-European population-based cohort." Diabetes Metab Res Rev **21**(6): 515-24.

Bouchard, C., F. T. Dionne, et al. (1992). "Genetics of aerobic and anaerobic performances." Exerc Sport Sci Rev **20**: 27-58.

Boule, N. G., E. Haddad, et al. (2001). "Effects of exercise on glycemic control and body mass in type 2 diabetes mellitus: a meta-analysis of controlled clinical trials." JAMA **286**: 1218-1227.

Calbet, J. A., J. S. Moysi, et al. (1998). "Bone mineral content and density in professional tennis players." Calcif Tissue Int **62**: 491-496.

Caspersen, C. J. (1987). "Physical inactivity and coronary heart disease." Physician and Sportsmedicine **15**(11): 43-44.
Deen, D. (2004). "Metabolic Syndrome: Time for Action." Am Fam Physician **69**(2875-82): 2887-8.
Del Coso, J., N. Hamouti, et al. (2010). "Aerobic fitness determines whole-body fat oxidation rate during exercise in the heat." Appl Physiol Nutr Metab. **35**(6): 741-8.
Dunstan, D. W., I. B. Puddey, et al. (1998). "Effects of a short-term circuit weight training program on glycaemic control in NIDDM." Diabetes Res Clin Pract **40**: 53-61.
Durstine, J. L., P. W. Grandjean, et al. (2002). "Lipids, lipoproteins, exercise." J Cardiopulm Rehabil **22**: 385-398.
Durstine, J. L. and W. L. Haskell (1994). "Effects of exercise training on plasma lipids and lipoproteins." Exer Sport Sci Rev **22**: 447.
Frayn, K. N. and P. F. Maycock (1980). "Skeletal muscle triaglycerol in the rat: methods for sampling and measurement, and studies of biological variability." J Lipid Res **21**(1): 139-144.
Gami, A. S., B. J. Witt, et al. (2007). "Metabolic syndrome and risk of incident cardiovascular events and death: a systematic review and meta-analysis of longitudinal studies." J Am Coll Cardiol **49**: 403-414.
Gardner, M. J. and D. G. Altman (1986). "Confidence intervals rather than P values: estimation rather than hypothesis testing." Br Med J **292**: 746-750.
Helge, J. W., R. Damsgaard, et al. (2008). "Low-intensity training dissociates metabolic from aerobic fitness." Scand J Med Sci Sports **18**(1): 86-94.
Hildrum, B., A. Mykletun, et al. (2007). "Age-specific prevalence of the metabolic syndrome defined by the International Diabetes Federation and the National Cholesterol Education Program: the Norwegian HUNT 2 study." BMC Public Health **7**: 220.
Hwang, L. C., C. H. Bai, et al. (2007). "Gender difference on the development of metabolic syndrome: a population-based study in Taiwan." Eur J Epidemiol **22**(12): 899-906.
INE. (2006). "Encuesta Nacional de Salud; Instituto Nacional de Estadística." from http://www.ine.es/jaxi/tabla.do.
Kashyap, S. R. and R. A. De Fronzo (2007). "The insulin resistance syndrome: physiological considerations." Diab Vasc Dis Res **4**: 13-19.
Katzmarzyk, P. T., A. S. Leon, et al. (2003). "Targeting the metabolic syndrome with exercise: evidence from the HERITAGE Family Study." Med Sci Sports Exerc **35**: 1703-1709.
Kiernan, M., A. C. King, et al. (2001). "Men gain additional psychological benefits by adding exercise to a weightloss program." Obes Res **9**: 770-777.
Larsen, S., I. Ara, et al. (2009). "Are substrate use during exercise and mitocondrial respiratory capacity decreased in arm and leg muscle in type 2 diabetes?" Diabetologia **52**(7): 1400-8.
Lillioja, S., C. Bogardus, et al. (1985). "Relationship between insulin-mediated glucose disposal and lipid metabolism in man." J Clin Invest **75**(4): 1106-15.
Lopez-Candales, A. (2001). "Metabolic syndrome X: a comprehensive review of the pathophysiology and recommended therapy." J Med **32**: 283-300.
Lucía, A., J. Hoyos, et al. (2000). "Heart rate and performance parameters in elite cyclists: a longitudinal study." Med Sci Sports Exerc. **32**(10): 1777-82.
Martínez-González, M. A., C. López-Fontana, et al. (2005). "Validation of the Spanish version of the physical activity questionnaire used in the Nurses' Health Study and the Health Professionals' Follow-up Study." Public Health Nutr **8**: 920-7.
Martínez-Vizcaíno, V., M. S. Martínez, et al. (2010). "Validity of a single-factor model underlying the metabolic syndrome in children: a confirmatory factor analysis." Diabetes Care **33**(6): 1370-2.
McArdle, W. D., F. I. Katch, et al. (1996). Physical activity, health and aging. Exercise Physiology; energy, nutrition and human performance. D. Balado. Baltimore, Williams and Wilkins**:** 635-655.
McCulloch, D. K., P. Bingley, et al. (1993). "Comparison of bolus and infusion protocols for determining acute insulin response to intravenous glucose in normal humans." Diabetes care. **16**: 911-15.
Mikines, K. J., B. Sonne, et al. (1988). "Effect of physical exercise on sensitivity and responsiveness to insulin in humans." Am J Physiol. **254**: E248-59.
Milesis, C. A., M. L. Pollock, et al. (1976). "Effects of different durations of physical training on cardiorespiratory function, body composition, and serum lipids." Res Q. **47**(4): 716-25.
Mora-Rodriguez, R., J. Coso, et al. (2010). Changes in Markers for Cardiovascular and Metabolic Disease Risk Evident after Only 1-2 weeks of a High Saturated Fat Diet in Overweight adults. Diabetes.
Passonneau, J. V. and V. R. Lauderdale (1974). "A comparison of three methods of glycogen measurement in tissues." Anal. Biochem. **60**: 405-412.
Position stand (1993). "Physical activity, physical fitness, and hypertension." Med Sci Sports Exerc. **25**(10): i-x.

Reaven, G. (2006). "The metabolic syndrome: is this diagnosis necessary?" Am J Clin Nutr **83**: 1237-1247.
Reaven, G. M. (1988). "Role of insulin resistance in human disease." Diabetes **1595-1607**(37).
Reaven, G. M. (1995). "Characteristics of metabolic syndrome." Endocrinol Metab **2**(Suppl B): 37-42.
Remsberg, K. E., N. L. Rogers, et al. (2007). "Sex differences in young adulthood metabolic syndrome and physical activity: the Fels longitudinal study." Am J Hum Biol **19**(4): 544-50.
Ren, J. M., C. F. Semenkovic, et al. (1994). "Exercise induces rapid increases in GLUT4 expression, glucose transport capacity, and insulin-stimulated glycogen storage in muscle " J Biol Chem **269**: 14396-14401.
Sady, S. P., E. M. Cullinane, et al. (1988). "Elevated high-density lipoprotein cholesterol in endurance athletes is related to enhanced plasma triglyceride clearance." Metabolism **37**(6): 568-72.
Sánchez-Chaparro, M., E. Calvo-Bonacho, et al. (2008). "Occupation-related differences in the prevalence of metabolic syndrome." Diabetes Care **31**(9): 1884-5.
Sanchez-Medina, L., C. E. Perez, et al. (2010). "Importance of the propulsive phase in strength assessment. ." Int J Sports Med **31**: 123-129.
Schenk, S., M. P. Harber, et al. (2009). "Improved insulin sensitivity after weight loss and exercise training is mediated by a reduction in plasma fatty acid mobilization, not enhanced oxidative capacity." J Physiol **587**(20): 4949-4961.
Schenk, S. and J. F. Horowitz (2007). "Acute exercise increases triglyceride synthesis in skeletal muscle and prevents fatty acid-induced insulin resistance." J Clin Invest **117**(6): 1690-8.
Solera Martínez, M., S. López Martínez, et al. (2011). "Validez de un modelo de único factor en el síndrom metabólico en adultos." Rev Española de Cardiologia.
Stensvold, D., A. E. Tjønna, et al. (2010). "Strength training versus aerobic interval training to modify risk factors of metabolic syndrome." J Appl Physiol **108**: 804-810.
Tremblay, A., E. Doucet, et al. (1999). "Metabolic fitness in active reduced-obese individuals." Obes Res. **7**(6): 556-63.
Tremblay, A., E. Fontaine, et al. (1986). "The effect of exercise-training on resting metabolic rate in lean and moderately obese individuals." Int J Obes **10**(6): 511-7.
Tura, A., S. Sbrignadello, et al. (2010). "An empirical index of insulin sensitivity from short IVGTT: validation against the minimal model and glucose clamp indices in patients with different clinical characteristics." Diabetologia **53**: 144-152.
Venables, M. C. and A. E. Jeukendrup (2008). "Endurance training and obesity: effect on substrate metabolism and insulin sensitivity." Med Sci Sports Exerc **40**: 495-502.
Vickers, A. J. and D. G. Altman (2001). "Statistics notes: analysing controlled trials with baseline and follow up measurements." Br Med J **323**: 1123-1124.
Vincent, W. J. (1999). Statistics in kinesiology. Champaign, IL, Human Kinetics.
Wilson, P. W., R. B. D'Agostino, et al. (2005). "Metabolic syndrome as a precursor of cardiovascular disease and type 2 diabetes mellitus." Circulation **112**(20): 3066-72.
Wu, T., X. Gao, et al. (2009). "Long-term effectiveness of diet-plus-exercise interventions vs. diet-only interventions for weight loss: a meta-analysis." Obes Rev. **10**(3): 313-23.
Zuti, W. B. and L. A. Holding (1976). "Comparing diet and exercise as weight reduction tools." Physician and Sportsmedicine **4**: 49-53.

HOJA DE INFORMACION Y CONSENTIMIENTO INFORMADO DEL PARTICIPANTE

Título del estudio. **"Efectos de 16 semanas de entrenamiento combinado aerobico-fuerza en los componentes individuales del Síndrome Metabólico; evolución temporal de las mejoras"**

Equipo de investigadores:

- **Dr. Ricardo Mora Rodríguez (Responsable del proyecto)**. Catedrático de Universidad, Escuela de Enfermería y Fisioterapia; Facultad de Ciencias del Deporte. Universidad de Castilla-La Mancha (UCLM)
- **Juan Fernando Ortega Fonseca.** Licenciado en Medicina y Cirugía. Especialidad en Medicina Deportiva. Universidad El Bosque (Colombia).
- **Ignacio Ara Royo.** Titular de Universidad. Facultad de Ciencias del Deporte. Universidad de Castilla-La Mancha (UCLM).
- **Vicente Martínez Vizcaino**. Catedrático de Escuela Universitaria. Escuela de Enfermería. Universidad de Castilla-La Mancha (UCLM).
- **Jorn Helge**. Profesor-Investigador senior, Dept. Biomedical Sciences. University of Copenhagen.

Objetivo. El sobrepeso conduce con los años a la obesidad abdominal y esta al padecimiento de trastornos metabólicos (hipercolesteremia, diabetes) que hacen que se eleve el riesgo de padecer una enfermedad cardiovascular. El objetivo de este estudio es contrarrestar este cuadro clínico (síndrome metabólico) a través de la realización de un programa de ejercicio físico de 4 meses de duración que nosotros les administraremos de manera totalmente gratuita. Queremos conocer las consecuencias del ejercicio físico en los factores asociados al sobrepeso y obesidad (nivel alto de lípidos en sangre, hipertensión y pre-diabetes) y compararlo con una dieta donde se reducen las calorías.

Requisitos para participar. Usted y otros 119 adultos de entre 25 y 65 años participarán en este estudio. Todos ustedes tendrán sobrepeso y no participarán en actividades deportivas. La mitad de los participantes además de tener sobrepeso tendrán al menos 3 de los siguientes 5 factores; i) presión arterial alta, ii) glucosa en sangre alta en ayunas, iii) triglicéridos altos en sangre, iv) HDL colesterol bajo en sangre, v) obesidad abdominal. Si usted tiene 3 de estos 5 factores estará dentro del grupo denominado "síndrome metabólico". Los participantes que tengan sobrepeso solamente serán el "grupo control". Este grupo no deberán tener ninguna patología ni estar medicándose durante la duración del estudio (excepto contraceptivos orales). Si usted es mujer, no deberá estar embarazada.

Los participantes del grupo "síndrome metabólico" no deberán tener ninguna otra patología endocrina diferente a intolerancia a los carbohidratos o hipelipidemia. Sus niveles hormonales (excepto la insulina) deberán encontrarse en rangos de normalidad clínica. Las personas que se encuentre en tratamiento para hipertensión o que reciban medicamentos hipoglucemiantes o hipolipemiantes podrán también participar. En estos participantes, se solicitará la opinión de su médico de familia antes de enrolarles en el estudio. Durante el estudio su médico le podrá ajustará el tratamiento de acuerdo a las necesidades particulares de cada individuo y a su grado de evolución durante el estudio. El equipo investigador solicitará al medico información sobre el cambio en la dosis de su medicación para poder interpretar los resultados.

Otros criterios de exclusión serán: cirugía reciente, enfermedad cardiovascular (especialmente enfermedad coronaria, enfermedad valvular cardiaca, insuficiencia cardiaca, arritmias ventriculares complejas), enfermedades renales, hepáticas, respiratorias o neuromusculares.

Grupos en el estudio. Dentro de cada grupo ("control" y "síndrome metabólico") a usted se le asignará de manera aleatoria uno de los siguientes 2 tratamientos: Entrenamiento físico durante 4 meses o, reducción de las calorías en la dieta durante 4 meses. Por lo tanto, usted pasará a pertenecer a uno de los 4 grupos experimentales que se resumen en la tabla de debajo:

GRUPO CONTROL (sobrepeso solo)		GRUPO SÍNDROME METABÓLICO (sobrepeso más 3 de 5 factores)	
ENTRENAMIENTO FÍSICO	DIETA	ENTRENAMIENTO FÍSICO	DIETA

Exploración médica y prueba de ejercicio antes de comenzar el estudio. Antes de comenzar el estudio se le realizará un historial medico con exploración, y se le pedirá que se someta a una prueba de esfuerzo pedaleando en un ciclo-ergómetro. En reposo y durante el ejercicio medimos la respuesta de su corazón utilizando un electrocardiógrafo y las presiones arteriales para descartar anomalías cardiacas o de su sistema cardiovascular. Además, durante la prueba se recogerá parte del aire que espira para calcular el consumo de oxígeno y su capacidad aeróbica máxima. Todos estos procedimientos serán realizados por el médico especialista en medicina del deporte del equipo investigador. Es posible que al final de la prueba usted se sienta fatigado/a. En raras ocasiones se puede experimentar tras el esfuerzo, mareos, desarreglos gastrointestinales e incluso (en personas con una patología cardiaca no diagnosticada) muerte súbita. Si durante esta prueba o en las otras de este estudio usted nota mareos, dificultad en la respiración, o dolor repentino nos lo debe comunicar inmediatamente.

Grupos de Dieta: Si usted consiente a participar en este estudio, y le toca ser parte del grupo de dieta, se le someterá a una dieta donde se reducirá la cantidad de calorías que ingiere en unas 300-500 por día. Esta reducción no es drástica, pero la dificultad entraña en el compromiso de usted para mantenerla durante 4 meses. Tras un análisis de su dieta habitual por un experto en dietética y nutrición se le dará las instrucciones para modificar su dieta sin que haya pérdida de los nutrientes necesarios. Se le pedirá que todos los días mantenga un registro de la comida que ingiere y de su peso corporal nada mas levantarse. El equipo de investigadores le suministrará básculas para pesar alimentos y su peso corporal así como un programa informático de recuento calórico. Cada 4 días nos traerá los datos recogidos (calorías ingeridas y pesos corporales) para ser analizados y realizar los ajustes necesarios. En dichos días se le medirá la grasa corporal (bioimpedancia) así como la tensión arterial.

Grupo Entrenamiento Físico: El ejercicio será realizado bajo nuestra supervisión en una bicicleta estática, tapiz rodante o maquinas similares. El programa de entrenamiento consistirá en 3 sesiones semanales de ejercicio aeróbico interválico y 1 sesión semanal de entrenamiento de fuerza. El ejercicio aeróbico consistirá en un trabajo continuado de 55 min de duración por entrenamiento. Las sesiones de entrenamiento de la fuerza consistirán en sesiones semanales de fortalecimiento enfocadas en miembros superiores, tronco y miembros inferiores. La carga de trabajo se incrementará según se vayan produciendo las adaptaciones al entrenamiento. En cada sesión de ejercicio, se le medirá la frecuencia cardiaca y su sensación subjetiva del esfuerzo. Cada 2 semanas se le medirá la presión arterial y mediante el análisis de su respiración calcularemos cuanta grasa oxida durante el ejercicio en la sesión de ejercicio aeróbico.

Campus tecnológico "Antigua Fábrica de Armas" Avda. de Carlos III s/n 45071 – TOLEDO
Telefono: 925.26.88.00 Ext: 5510 Fax: 925.26.88.46 Email: ricardo.mora@uclm.es

Mediciones a las que se someterá durante el experimento

Se le solicitará que acuda al laboratorio por la mañana (7-9 am), sin haber desayunado ni haber entrenado el dia anterior en las siguientes ocasiones:

a) antes del inicio del entrenamiento-dieta (día 1)
b) después de la primera semana de entrenamiento (+ 1 sem)
c) después de 1 mes de intervención (+ 1 m)
d) después de 2 meses de intervención (+ 2 m)
e) después de 3 meses de la intervención (+ 3 m)
f) después de 4 meses de intervención (+ 4 m)
g) 1 mes posterior a la terminación de la intervención (para evaluar recaída)
h) 6 meses después de terminar la intervención (para evaluar recaída)

<u>Estimación de la composición corporal.</u> Se pesará desnudo/a y se le tallará. A continuación se medirá el grosor de los pliegues de ciertas partes de su piel (dorso del brazo, muslo, etc), para la estimación del porcentaje de grasa corporal. También se le calculará la cantidad de grasa corporal con un aparato que hace circular por su cuerpo una débil corriente eléctrica que no sentirá (bioimpedancia eléctrica).

<u>Variables cardiovasculares y metabolismo de reposo.</u> Tras estas mediciones, se tumbará en una camilla, se le pondrá una banda elástica alrededor del pecho para medir su frecuencia cardiaca y una máscara alrededor de su boca y nariz para recoger el aire espirado. Dicha máscara le permite respirar normalmente. Este aire se analizará durante 15 minutos para calcular su metabolismo basal (calorías que gasta en reposo).

<u>Test intravenoso de tolerancia a la glucosa.</u> Tras estas mediciones de reposo se le colocará una vía flexible en una vena del antebrazo por la que se le extraerá una muestra de sangre (unos 5 mililitros). Después se le inyectará en unos 4 minutos 35 gramos de glucosa disuelta esterilizada. A partir de entonces, se recogerá una muestra de sangre a los 1, 2, 4, 6, 8, 10, 12, 14, 20, 30, 40 y 60 minutos. En estas muestras analizaremos la concentración de glucosa y de insulina en su sangre.

<u>Perfil lipídico</u>. En la muestra de sangre que tomamos antes de inyectar la glucosa se medirá la concentración de triglicéridos, colesterol total y sus fracciones y el tipo de ácidos grasos en su sangre (saturados vs mono-poliinsaturados). También, analizaremos en su sangre parámetros de daño en el hígado como son las transaminasas y la proteína C reactiva.

<u>Obtención de biopsia muscular</u>. Seguidamente se le extraerá una pequeña muestra del músculo del muslo. Tras la limpieza y desinfección de la piel se le aplicará un anestésico de acción local similar al que usan los dentistas y se extraerá una pequeña porción de tejido muscular (aproximadamente del tamaño de 2-3 granos de arroz) a través de una incisión en la piel del muslo. Tras la extracción la herida se cerrará con unas tiritas de aproximación (sin puntos) y un vendaje compresor que deberá llevar durante al menos 48 horas.

<u>Riesgos y molestias</u>. Existe el riesgo de desarrollar un pequeño hematoma (derrame) en el lugar donde estaba la vía en el brazo. Este riesgo se reduce si se aplica presión durante 10 minutos tras extraer la vía. Tanto en la punción del brazo como en las de la piel del muslo existe el riesgo de infección, que se minimiza con los procedimientos estériles que seguimos. Algunas personas se marean durante las

punciones en la piel del brazo o muslo pero los riesgos de accidente por mareo se reducen al realizar dicha punción con usted tumbado/a en la camilla. Por ultimo, algunas personas comentan una cierta pérdida de sensibilidad en el área cercana a la biopsia muscular (2-3 cm), pero esta pérdida de sensibilidad en la piel es temporal y remite en 2-3 semanas.

HOJA DE CONSENTIMIENTO INFORMADO

NOMBRE Y APELLIDOS ____

Fecha Nacimiento _____________ DNI ____________________________

Dirección ____

Teléfono de contacto _____________________________

NOMBRE INVESTIGADOR PRINCIPAL *RICARDO MORA RODRÍGUEZ*

Laboratorio de Fisiología del Ejercicio. Universidad de Castilla La Mancha. Toledo

1. He leído la hoja de información del proyecto y he tenido la posibilidad de discutir los detalles con el investigador principal y preguntarle cualquier tipo de dudas. El responsable del proyecto me ha explicado el propósito de las pruebas que van a realizarme y he entendido completamente todo lo que se me ha explicado.
2. Estoy de acuerdo en tomar parte de este estudio y entiendo que soy completamente libre para abandonarlo en cualquier momento que desee o negarme a la realización de alguno de los procedimientos de medición.
3. Entiendo que las pruebas realizadas son parte de un proyecto de investigación que no me aportará ningún lucro personal sino que la participación es voluntaria. Los resultados de este estudio pretenden promover el conocimiento en Ciencias Biomédicas y entiendo que los procedimientos descritos han sido aprobado por un comité ético de investigación clínica.

Consiento total y libremente a participar en el proyecto titulado: Título del estudio. **"Efectos de 16 semanas de entrenamiento combinado aerobico-fuerza en los componentes individuales del síndrome metabólico; evolución temporal de las mejoras"** el cual me ha sido detalladamente explicado.

Firma del voluntario ______________________________________

Lugar y Fecha____

Confirmo que he explicado al voluntario (arriba nombrado) el propósito y riesgos de las pruebas que van a realizársele.

Firma del Investigador ____________________________________

Lugar y Fecha ____
